# Two New Neo-debromoaplysiatoxins—A Pair of Stereoisomers Exhibiting Potent Kv1.5 Ion Channel Inhibition Activities

**DOI:** 10.3390/md17120652

**Published:** 2019-11-21

**Authors:** Ting-Ting Fan, Hui-Hui Zhang, Yang-Hua Tang, Fan-Zhong Zhang, Bing-Nan Han

**Affiliations:** 1 Department of Development Technology of Marine Resources, College of Life Sciences and Medicine, Zhejiang Sci-Tech University, Hangzhou 310018, China; tawnie1994@sina.com (T.-T.F.); 18895602876@sina.com (H.-H.Z.); fancyzfz@163.com (F.-Z.Z.); 2 Department of Pharmacy, Graduate School, Hunan University of Chinese Medicine, Changsha 410208, China; tangyanghua@126.com

**Keywords:** marine cyanobacterium, *Lyngbya* sp., aplysiatoxin, stereoisomers, Kv1.5 inhibitory activity

## Abstract

A pair of stereoisomers possessing novel structures with 6/6/5 fused-ring systems, neo-debromoaplysiatoxin E (**1**) and neo-debromoaplysiatoxin F (**2**), were isolated from the marine cyanobacterium *Lyngbya* sp. Their structures were elucidated using various spectroscopic techniques including high resolution electrospray ionization mass spectroscopy (HRESIMS) and nuclear magnetic resonance (NMR). The absolute stereochemistry was determined by calculated electronic circular dichroism (ECD) and gauge-independent atomic orbital (GIAO) NMR shift calculation followed by DP4+ analysis. Significantly, this is the first report on aplysiatoxin derivatives with different absolute configurations at C9–C12 (**1**: 9*S*, 10*R*, 11*S*, 12*S*; **2**: 9*R*, 10*S*, 11*R*, 12*R*). Compounds **1** and **2** exhibited potent blocking activities against Kv1.5 with IC_50_ values of 1.22 ± 0.22 μM and 2.85 ± 0.29 μM, respectively.

## 1. Introduction

Aplysiatoxins (ATXs) are a class of biologically active dermatotoxins with anti-proliferative activity, tumor-promoting properties, pro-inflammatory actions, antileukemia activity, and antiviral activity [1,2,3,4,5,6]. It was first discovered in the digestive gland of the *Stylocheilus longicauda* [7,8]. Further research found that the toxin actually came from a cyanobacteria, which is a "prey" of the sea hares [4,9]. Recently, a series of ATX derivatives with Kv1.5 ion channel inhibitory activities have been discovered during our previous studies [10,11,12]. Due to the presence of some unstable functional groups such as hemiacetal and ketal, etc., ATXs easily underwent structural rearrangement to form a new structure. According to the structural characteristics, aplysiatoxins were to be divided into three categories: one with 6/12/6, 6/10/6, or 6/6/6 ABC tricyclic ring systems (e.g., Debromoaplysiatoxin, neo-debromoaplysiatoxin A, neo-debromoaplysiatoxin B) [7,10,13], the other with an AB spirobicyclic ring system (e.g., Oscillatoxin D and 30-methyloscillatoxin D) [14,15], and the third type with acyclic structures such as Nhatrangins [16].

**Figure 1:**
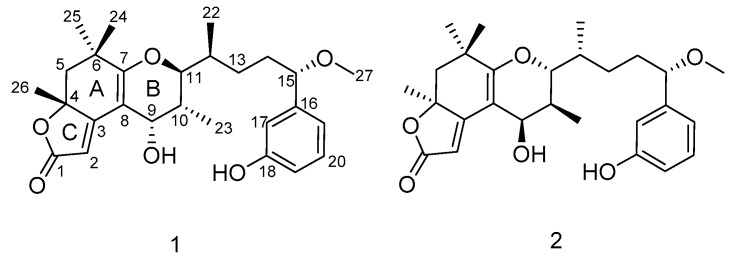
Structures of neo-debromoaplysiatoxin E (**1**) and neo-debromoaplysiatoxin F (**2**).

The Kv1.5 channel is considered to be a key target for the treatment of atrial tachyarrhythmias with minimal side effects [17,18,19]. At present, several Kv1.5 ion channel inhibitors have been discovered, and these include oxabispidines, benzamides, and phosphine oxides, etc. [20,21,22]. Among them are AVE0118 and AZD7009, which are in clinical phase II [23,24]. Vernakalent is currently the only Kv1.5 channel blocker on the market for clinical treatment of atrial fibrillation [25]. In addition, acacetin is considered to be one of the few natural products with selective Kv1.5 ion channel inhibitory activity [26,27]. In search of potent Kv1.5 inhibitors, our team isolated two new aplysiatoxin derivatives, neo-debromoaplysiatoxin E **(1**) and neo-debromoaplysiatoxin F (**2**) (Figure 1), a pair of stereoisomers with a new ABC tricyclic ring system (6/6/5 fused-ring system), from the marine cyanobacterium collected off the coast of Hainan Island, China. According to the 16S rRNA sequence analysis, this cyanobacterium constitutes a stable clade comprising the *Lyngbya* species (Supplementary Materials) [10]. Herein, we report the isolation, structure elucidation, and Kv1.5 blocking activities of compounds **1** and **2**. The absolute configurations of these compounds were established by a combination of calculated electronic circular dichroism (ECD) and DP4+ probability analysis based on NMR shift calculation. We report, for the first time, on aplysiatoxin derivatives with different absolute configurations at C9–C12 (**1**: 9*S*, 10*R*, 11*S*, 12*S*; **2**: 9*R*, 10*S*, 11*R*, 12*R*), and the exhibition of potent potassium channel Kv1.5 blocking activities (IC_50_ = 1.22 ± 0.22 μM and 2.85 ± 0.29 μM, respectively).

## 2. Results and Discussion

### 2.1. Structure Elucidation of the New Compounds

Neo-debromoaplysiatoxin E (**1**) was obtained as a colorless solid. The molecular formula of C_27_H_36_O_6_ with 10 degrees of unsaturation was deduced from HRESIMS data (*m/z* 457.2589 [M + H]^+^, calcd. 457.2585). The ^13^C, DEPT (distortionless enhancement by polarization transfer), and HSQC (heteronuclear single quantum coherence) NMR spectra of **1** indicated the presence of one carbonyl carbon (*δ*_C_ 176.3), five sp^2^ quaternary carbons (*δ*_C_ 174. 2, 167.4, 105.7, 144.9 and 158.8), two aliphatic quaternary carbons (*δ*_C_ 84.9 and 37.5), six methyl groups (one methoxy), three methylenes, five sp^3^-hybridized methines (three oxygenated), and five sp^2^-hybridized methines (*δ*_C_ 103.1, 114.3, 115.6, 130.4, and 119.2). Detailed ^1^H and ^13^C NMR analysis indicated the presence of a phenol ring substitution at *meta*-position. These functional groups required seven of the 10 degrees of unsaturation, so compound **1** was proposed to be tricyclic.

**Figure 2:**
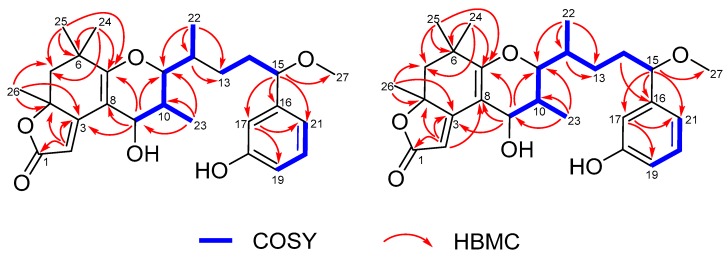
Key COSY and HMBC correlations of **1** and **2.**

The ^1^H-^1^H COSY (homonuclear chemical shift correlation spectroscopy) spectrum (Figure 2) indicated the presence of three independent spin systems: H-9/H-10/H-11/H_3_-23/H-12/H_3_-22, H_2_-13/H_2_-14/H-15, and H-19/H-20/H-21. The HMBC (heteronuclear multiple bond correlation) correlations from H_3_-26 to C-3, C-4, and C-5, from H_3_-25 to C-5, C-6, and C-7, from H_3_-24 to C-5, C-6, and C-7, and from H-9 to C-3, C-7, and C-8 established the structure of ring A and suggested the linkage of ring A to the spin system of H-9/H-10/H-11/H-12/H_3_-22. The HMBC correlations from H-2 to C-1, C-3, and C-4 confirmed the connection from C-1 to C-4. Based on its molecular formula and the chemical shifts of C-1 (*δ*_C_ 176.3) and C-4 (*δ*_C_ 84.9), it could be deduced that a five-membered lactone ring was formed (ring C). Considering the degrees of unsaturation, C-7 and C-11 were connected via an oxygen, which led to the determination of ring B. The HMBC correlations from H_3_-22 to C-11, C-12, and C-13, from H-15 to C-16, C-17, C-21, and C-27, and from H-17 to C-19 and C-21 connected the three independent spin systems, which were confirmed by ^1^H-^1^H COSY spectra. Accordingly, the planar structure of **1** was assigned (Figure 2).

Together with **1**, neo-debromoaplysiatoxin F (**2**) was also obtained as a colorless solid from the same fraction with different retention time (**2**: *t*_R_ 30.7 min; **1**: *t*_R_ 41.1 min). The HRESIMS data indicated a molecular formula of C_27_H_36_O_6_ for neo-debromoaplysiatoxin F (**2**), identical to that of **1**. The overall appearance of the NMR spectrum of **2** revealed close structural similarity between **1** and **2**, indicating the presence of the same 6/6/5 fused-ring system in both compounds. In addition, the planar structure of **2** elucidated by COSY and HMBC correlations (Figure 2) were the same as that of **1**, which implied that compounds **1** and **2** were a pair of stereoisomers. 

**Figure 3:**
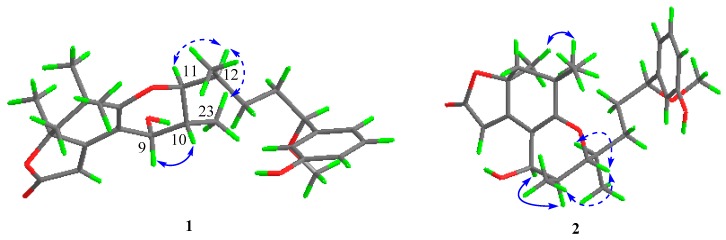
Key NOESY correlations of **1** and **2** (solid lines: *α*-orientation; dashed lines: *β*-orientation).

**Figure 4:**
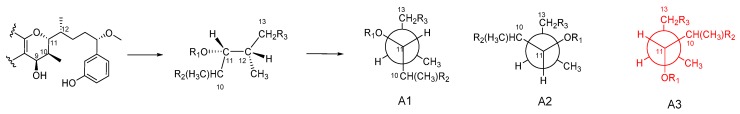
Newman projection analysis of the energy-equivalent isomers.

The relative configuration of **1** was determined by the NOESY (nuclear overhauser effect spectroscopy) spectrum and vicinal coupling constants. As shown in Figure 3, the key NOESY correlations of H-9 with H-10 implicated their relative syn relationships, which was consistent with the small couplings of H-9 to H-10 (*J*_9,10_=3.6 Hz). The cis relationship of H-12 with H_3_-23 was deduced from the NOESY correlation between H-12 and H_3_-23. Based on the NOESY correlation, we performed a Newman projection analysis of the energy-equivalent isomers using homonuclear coupling constant information and steric hindrance [28] (Figure 4). The ^1^H-^1^H coupling constant of 1.79 Hz between H-11 and H-12 suggested a *gauche* relationship between these two protons, consequently ruling out model A1. The three large groups (–OR_1_, –CH_2_R_2_, –CH(CH_3_)R_2_) were spatially close in model A2, causing a large steric hindrance, thus model A2 was also ruled out. The remaining model A3 fulfilled all criteria and, however, confirmed that the H-11 and H-12 were oriented in the same plane. The NOESY spectrum of **2** exhibited correlations between H-9/H-10, H_3_-23/H-11, and H-11/H12, revealing the same orientation of these protons as **1**. 

**Figure 5:**
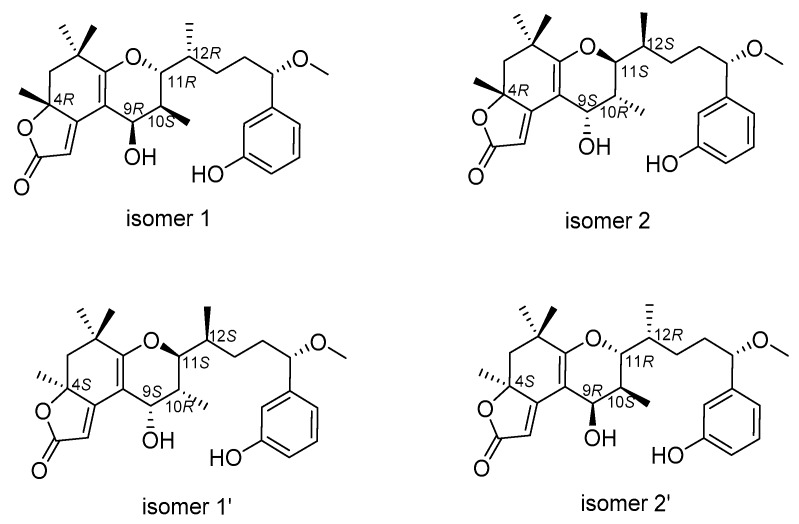
Possible configurations for **1** and **2**.

**Figure 6:**
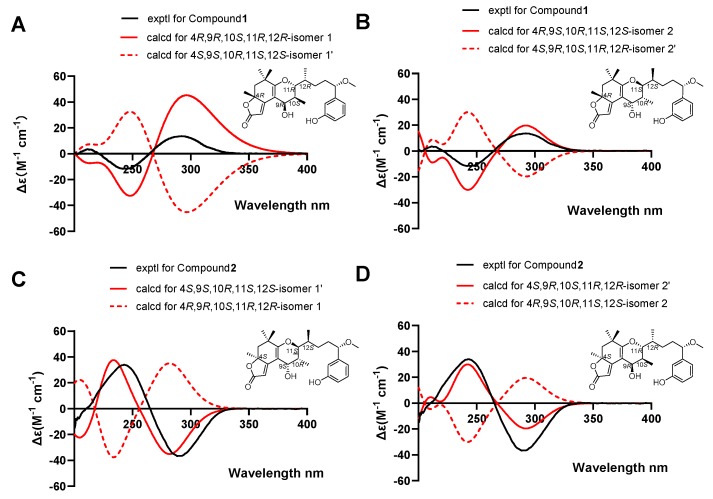
Electronic circular dichroism (ECD) spectra showing experimental and calculated analysis of isomers 1–4. (**A**) Calculated ECD spectra for isomer 1 was consistent with the experimental ECD spectra for compound **1**. (**B**) Calculated ECD spectra for isomer 2 was consistent with the experimental ECD spectra for compound **1**. (**C**) Calculated ECD spectra for isomer 1’ was consistent with the experimental ECD spectra for compound **2**. (**D**) Calculated ECD spectra for isomer 2’ was consistent with the experimental ECD spectra for compound **2**.

However, an additional chiral center, C-4, challenged us in two different manners while determining its relative configuration. First, the configuration of C-4 was difficult to be spatially related to the other four centers because of the remote distance. Secondly, it was difficult handling with the limited NOESY signal between H-4 and H-9. Therefore, we were prevented from establishing the relative configurations between them and determining the absolute configurations directly. Theoretically, there were four possible combinations for **1** and **2** regarding the absolute configurations at C4 and C9–C12, which were 4*R*, 9*R*, 10*S*, 11*R*, 12*R* (isomer 1), 4*S*, 9*S*, 10*R*, 11*S*, 12*S* (isomer 1’), 4*R*, 9*S*, 10*R*, 11*S*, 12*S* (isomer 2) and 4*S*, 9*R*, 10*S*, 11*R*, 12*R* (isomer 2’); among them, isomer 1 and isomer 1’ as well as isomer 2 and isomer 2’ possessed opposite configurations at C4 and C9–C12, respectively (Figure 5). The absolute configurations of C-4 for **1** and **2** was established by calculated electronic ECD. The positive and negative cotton effects at 245 nm and 292 nm observed in the experimental ECD spectrum of compound **1** were consistent with calculated ECD spectrum of isomer 1 and isomer 2 (Figure 6), suggesting the absolute configurations of **1** at C-4 was to be designated *R*, with C9–C12 not affecting the cotton effects. Furthermore, the calculated ECD spectrum of isomer 1’ and isomer 2’ were well matched in the experimental ECD spectrum of compound **2**, and thus the absolute configuration of **2** at C-4 was assigned as *S*. The above comparison of ECD spectra revealed two possible absolute configurations at C4 and C9–C12 for compounds **1** and **2** and they were as follows: **1** (4*R*, **9*R***, **10*S***, **11*R***, **12*R***/4*R*, **9*S***, **10*R***, **11*S***, **12*S***) and **2** (4*S*, **9*S***, **10*R***, **11*S***, **12*S***/4*S*, **9*R***, **10*S***, **11*R***, **12*R***). In order to confirm the absolute configurations at C9–C12 of compounds **1** and **2**, the improved probability DP4+ method was carried out.

**Figure 7:**
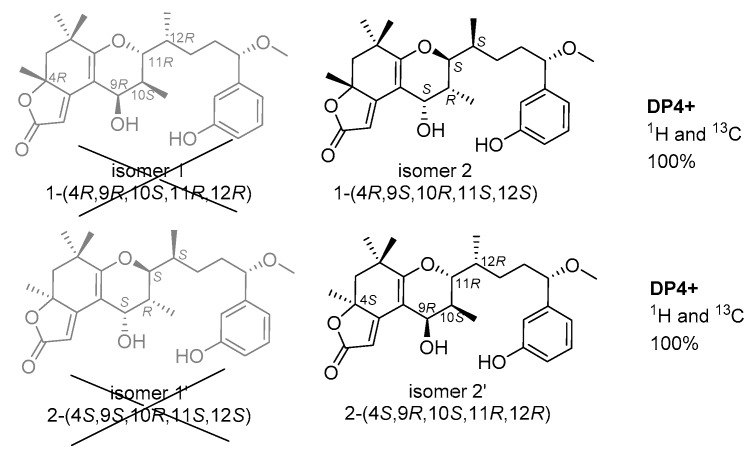
Density functional theory (DFT) studies and statistical DP4+ parameters found for **1** and **2**.

Gauge-independent atomic orbital (GIAO) calculations of ^1^H and ^13^C NMR chemical shifts were accomplished by density functional theory (DFT) at the MPWLPW91-SCRF (methanol)/6-311+g (d, p) level with the polarizable continuum model (PCM) [29]. The experimental and calculated data were analyzed by the improved probability DP4+ [30,31,32], suggesting that the most probable structure for **1** was isomer 2 (4*R*, 9*S*, 10*R*, 11*S*, 12*S*) with a probability of 100% based on ^1^H NMR, 100% based on ^13^C NMR, and 100% when carbon and proton shifts were included. The most probable structure for **2** was isomer 2’ (4*S*, 9*R*, 10*S*, 11*R*, 12*R*) with a probability of 99.98% based on ^1^H NMR, 100% based on ^13^C NMR, and 100% when carbon and proton shifts were included (Figure 7). Furthermore, from their structural similarities and hypothetical biosynthesis (Figure S19), it is likely that these two compounds had a common biosynthetic origin with aplysiatoxins as reported previously at the side chain, and thus the stereochemistry of C-15 was proposed to remain as *S* [8,9,10]. Therefore, the absolute configurations of **1** and **2** were assigned as 4*R*, 9*S*, 10*R*, 11*S*, 12*S*, 15*S* and 4*S*, 9*R*, 10*S*, 11*R*, 12*R*, 15*S*, respectively.

For all of the aplysiatoxin derivatives previously reported, the absolute configurations of C9–C12 are *R*, *S*, *R*, *R*. However, in this article, the altered absolute configurations at C9–C12 (*S*, *R*, *S*, *S*) of compound **1** is first reported. Through structural analysis, a plausible biosynthetic pathway is postulated in Figure S19 [33]. We speculated that the different absolute configurations of C9–C12 was not necessarily through structural rearrangement; it may start with different biosynthetic precursors possessing opposite chirality at C9–C12, followed by aldol reaction, nucleophilic addition, dehydration reaction, and reduction to form the final stereoisomers.

### 2.2. Biological Activities of the Isolated Compounds

**Figure 8:**
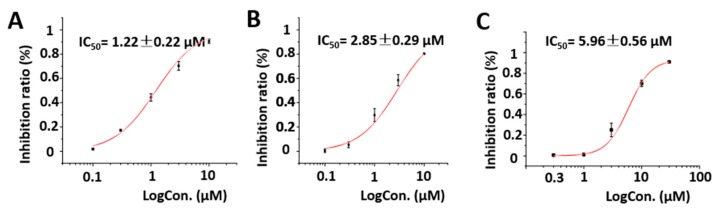
Dose–response study of **1** and **2** with Kv1.5 expression in chinese hamster ovary cells (CHO) at holding potential (HP) of -80 mV. Data points represent mean ± SEM of 3–5 measurements. Solid curve fits to the Hill equation. (**A**) Inhibitory effect of **1** showed an IC_50_ value of 1.22 ± 0.22 μM. (**B**) Inhibitory effect of **2** showed an IC_50_ value of 2.85 ± 0.29 μM. (**C**) Inhibitory effect of acacetin showed an IC_50_ value of 5.96 ± 0.56 μM.

The Kv1.5 (ultra-fast-delay rectifier potassium channel) mediation of ultra-rapid delayed rectifier K^+^ current (IKur) is the main current in the repolarization process of cardiomyocyte action potentials. This current is only expressed in human atrial muscle and provides a promising target for precision therapeutic drugs aimed at atrial fibrillation [17,18,19]. Additionally, our previous research has highlighted the potential of aplysiatoxins as ion channel blockers, specifically the selective blocking of Kv1.5. [10,11,12]. Following this, a dose-response study for neo-debromoaplysiatoxin E (**1**), neo-debromoaplysiatoxin F (**2**), and positive control acacetin were undertaken to evaluate their inhibitory activities against Kv1.5. Compounds **1** and **2** showed potent inhibitory activities against Kv1.5, with IC_50_ values of 1.22 ± 0.22 μM and 2.85 ± 0.29 μM, respectively, and the inhibitory activity was much higher than the positive control acacetin (IC_50_ = 5.96 ± 0.56 μM) (Figure 8). 

### 2.3. Molecular Docking Analysis of the Isolated Compounds

**Figure 9:**
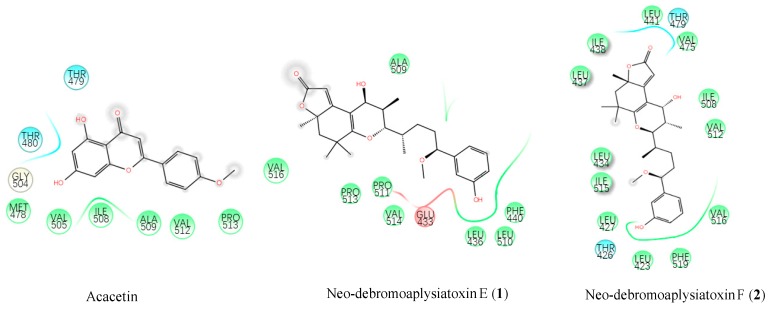
Ligand interaction map of acacetin, compound **1**, and compound **2.** Interacting proteins are shown in green and cyan. The red amino acids are negatively charged, the cyan amino acids are positively charged, and the yellow and green amino acids are hydrophobic.

We performed molecular docking analyses to complement our knowledge on the interaction between compounds **1** and **2** and the Kv1.5 channel. The Kv1.5 3D homology model was constructed based on the crystal structure of Kv1.2 voltage-gated potassium ion channels, both of which shared 90% similarity. According to the binding site for the known Kv1.5 channel blocker, acacetin (positive control), amino acids 480–512 in the Kv1.5 S6 domain was chosen as the binding region [27]. The docking results showed that compound **1** and acacetin had similar hydrophobic interaction with Ala509 and Pro513, while compound **2** interacted with Thr479, Val512, and Val516 (Figure 9). 

In addition, we tried to analyze the correlation between the docking results and the ion channel activity. The docking results showed that neo-debromoaplysiatoxin E (**1**) and neo-debromoaplysiatoxin F (**2**) had strong binding affinities of −34.337 kcal mol^−1^ and −38.494 kcal mol^−1^, respectively, but acacetin had relatively weaker binding at −26.206 kcal mol^−1^. However, the Kv1.5 homology model was formed on the basis of the Kv1.2 protein crystal, so in this report, we believe the absolute value of the binding energy may not precisely reflect the experimental activity results, and furthermore, molecular docking was only used to determine that these two compounds (neo-debromoaplysiatoxin E and neo-debromoaplysiatoxin F) had a similar hydrophobic interactions within the same docking region of the positive control compound acacetin.

## 3. Experimental Section

### 3.1. General Experimental Procedures

Optical rotation data were recorded on a Jasco P-2000 polarimeter (Jasco, Hachioji-shi, Tokyo, Japan). UV and IR (KBr) spectra were obtained on a UV/EV300 spectrometer (Thermo scientic, Waltham, MA, USA) and a Nicolet 6700 instrument (Thermo scientic, Waltham, MA, USA), respectively. ^1^H and ^13^C NMR spectra were acquired on an Agilent 600 MHz spectrometer (Agilent Technologies, Santa Clara, CA, USA). with CD_3_OD (*δ*_H_ 4.87 and *δ*_C_ 49.00) as the solvent and internal standard. HRESIMS data were collected with a Waters Q-Tof micro YA019 mass spectrometer (Waters, Milford, MA, USA). A Waters 1525 series instrument (Waters, Milford, MA, USA) equipped with Waters XBridge Prep C-18 column (5 μm, 10 mm × 250 mm, Waters, Milford, MA, USA) and a 2998 photodiode array detector (Waters, Milford, MA, USA) was used for the high-performance liquid chromatography (HPLC) analysis. Silica gel 60 (200–300 mesh; Yantai, China) and octadecylsilyl (ODS) (50 μm, YMC, Kyoto, Japan) were used for the column chromatography. Analytical thin-layer chromatography was carried out on a silica gel 60 F254 plates (Merck, Darmstadt, Germany).

### 3.2. Materials

The cyanobacterium *Lyngbya* sp. was collected from the harbor of Sanya, Hainan province, China, in November 2016. The sample was identified by Prof. Bing-Nan Han (Zhejiang Sci-Tech University, Zhejiang, China), and the specimen was frozen after transport to the laboratory. A voucher specimen (voucher number: BNH-201606; genebank accession numbers: MH636576) has been well deposited in Zhejiang Sci-Tech University.

### 3.3. Extraction and Isolation

The thawed cyanobacterium *Lyngbya* sp. (150 g, dry weight) was thoroughly extracted five times with 1 L MeOH/CH_2_Cl_2_ (2:1, v/v). The extract was suspended with 1 L MeOH/H_2_O (9:1, v/v) and extracted three times with CH_2_Cl_2_ to obtain 20 g crude. Silica gel VLC (vacuum liquid chromatography) was performed using gradients PE/EtOAc (5:1, 2:1, 1:1, 1:2, 1:5, 0:1, v/v) to yield seven fractions (Fr.A–G). Afterwards, Fr. D (800 mg) was separated by reverse-phase ODS column chromatography (10−100% MeCN/H_2_O, 180 min, flow rate 20 mL/min, UV detection at 190 nm) to obtain 21 components Fr. D1–D21. Subsequently, the subfractions FR.D.12, and FR.D.13 were purified by preparative HPLC (Waters SunFire Prep C18, 45.0% MeCN/H_2_O, 8.0 mL/min, UV detection at 190 nm) to afford neo-debromoaplysiatoxin E (3.1 mg, *t*_R_ 41.1 min) and neo-debromoaplysiatoxin F (3.8 mg, *t*_R_ 30.7 min). During the whole process, no acid/base reagents was added. The collected samples were immediately transferred to reasonable temperature rotary evaporation (less than 40 °C), protected from light, and without leaving it overnight. Meanwhile, an aprotic solvent acetonitrile was used as a mobile phase for the liquid chromatography. The HPLC integral analysis showed that the purity of Neo-debromoaplysiatoxin E was 94.45%, and the purity of neo-debromoaplysiatoxin F was 90.09%.

Neo-debromoaplysiatoxin E (**1**): Colorless solid; [α${]}_{D}^{25}$ +79.2 (0.07, MeOH); UV (MeOH) *λ*_max_ (log *ε*) 204 (4.04), 294 (4.08) nm; ECD (MeOH): *λ*_max_ (Δ*ε*) 214(3.3), 244.5(–11.5), 295(13.4); IR (KBr) *υ*_max_ 3415, 2927, 1653, 1630, 1618, 1460, 1450, 1400, 1384, 1337 cm^−1^; ^1^H and ^13^C NMR data, see Table 1; HRESIMS *m/z* 457.2589 [M + H]^+^ (calcd. for C_27_H_37_O_6_, 457.2585).

Neo-debromoaplysiatoxin F (**2**): Colorless solid; [α${]}_{D}^{25}$ –46.2 (0.07, MeOH); UV (MeOH) *λ*_max_ (log *ε*) 203 (3.97), 293 (4.09) nm; ECD (MeOH): *λ*_max_ (Δ*ε*) 243 (34.0), 288.5(–36.7); IR (KBr) *υ*_max_ 3450, 2933, 1698, 1618, 1460, 1402, 1384, 1337 cm^−1^; ^1^H and ^13^C NMR data, see Table 1; HRESIMS *m/z* 457.2579 [M + H]^+^ (calcd. for C_27_H_37_O_6_, 457.2585).

### 3.4. ECD Calculations

ECD calculations were carried out as described previously [10]. Using the Merck molecular force field (MMFF) in the Spartan 10 to search for conformations, the conformers with Boltzmann-population of over 5% were chosen to be optimized at the B3LYP/6-31 + g (d, p) level by the conductor polarizable continuum model (CPCM) (MeOH as the solvent). The theoretical calculation of ECD was conducted in MeOH using time-dependent density functional theory (TD-DFT) at the B3LYP/6-31+g (d, p) level for all conformers of the compounds [34]. Rotatory strengths for a total of 30 excited states were calculated. pecDis 1.6 (University of Würzburg, Würzburg, Germany) and GraphPad Prism 5 (University of California San Diego, San Diego, CA, USA) were used to generate ECD spectra.

### 3.5. NMR Calculations

Monte Carlo conformational searches were carried out by means of the Spartan’s 10 software (Spartan Software, San Francisco, CA, USA) using Merck molecular force field (MMFF). The conformers with a Boltzmann-population of over 1% were chosen for NMR calculations, and then the conformers were initially optimized at B3LYP/6-31g (d, p) level in gas. Meanwhile, gauge-independent atomic orbital (GIAO) calculations of ^1^H and ^13^C NMR chemical shifts were accomplished by density functional theory (DFT) at the mPWLPW91-SCRF (methanol)/6-311+g (d,p) level with the PCM solvent continuum model in Gaussian 09 software (Gaussian, Wallingford, CT, USA) [29]. The calculated NMR data of the lowest energy conformers were averaged according to the Boltzmann distribution theory and their relative Gibbs free energy. The ^1^H and ^13^C NMR chemical shifts for tetramethyl silane (TMS) were calculated by the same protocol and used as reference. The experimental and calculated data were analyzed by the improved probability DP4+ method for isomeric compounds [30,31,32]. A significantly higher DP4+ probability score of a compound suggested the correctness of its configuration.

### 3.6. Measurement of Ion Channel Inhibition Activity

The Kv1.5 ion channel was stably expressed by CHO cells [35,36], and the blocking activities of the compounds against Kv1.5 was recorded by whole cell patch clamp technique. CHO cells were grown to a density of 80%, digested with trypsin and transferred to 35 mm petri dish. The cells were cultured in dulbecco’s modified eagle medium/nutrient mixture F-12 (DMEM/F 12) (10% fetal bovine serum (FBS) + penicillin/streptomycin (P/S)) medium in an incubator containing 5% CO_2_ at 37 °C for 24 hours. Subsequently, the cells were transferred to a tank perfused with extracellular fluid (NaCl, 137 nM; KCl, 4 nM; CaCl_2_, 1.8 nM; MgCl_2_, 1 mM; hepesfreeacid (HEPES), 10 mM; glucose 10 mM; PH 7.4 (titrated by NaOH)). Intracellular fluid (KAspartate, 130 mM; MgCl_2_, 5 mM; EGTA, 5 mM; Hepes, 10 mM; Tris-ATP, 4 mM; PH 7.2 (titrated by KOH)) stored in a −80 °C refrigerator, was used to fill the electrode after melting. Compounds were dissolved in dimethyl sulfoxide (DMSO) and then added into the extracellular fluid. The cells were clamped at −80 mV and then depolarized to 20 mV with a square wave lasting for 2 seconds to obtain Kv1.5 current. The program was repeated every 20 seconds. After stabilization, the extracellular fluid containing compounds at different concentration were perfused and the intensity of the block was calculated. Data collection and analysis were conducted on pCLAMP 10 (Molecular Devices, Union City, CA, USA).

### 3.7. Molecular Modeling and Docking

The Prime 3.3 (Schrodinger LLC, New York, NY, USA) module was used for homology modelling in the modeling software Schrodinger suite 2015. The Kv1.2 channel 2.9 A° crystal structure (PDB ID: 2A79) was used as a template, and a homology modeling program was applied to generate a 3D homology model of the open state Kv1.5 channel [12,27]. The stereochemistry of the hybrid modeled structure was verified by PROCHECK (http://nihserver.mbi.ucla.edu/SAVES). The final predictive model was structurally optimized using the Amber 12.0 (Schrodinger LLC, New York, NY, USA) molecular dynamics simulation package. The ligand structures were prepared by LigPrep, and the Kv1.5 channel was also prepared by protein preparation wizard (Schrodinger LLC, 2010, New York, NY, USA) (Maestro 11.0 version) to minimize the energy of the generated protein. The protein was subjected to hydrogenation, dehydration, and hydrogen bond optimization by optimized potential for liquid simulations (OPLS). A grid file was then generated using Glide Grid, and automated docking of acacetin (positive control) and compounds **1** and **2** were performed using the Glide program. Molecular mechanics generalized born surface area (MM-GBSA) was used to calculate the binding free energy between ligand and protein. We used the ligand interaction module in the Maestro 10.2 software (Schrodinger LLC, New York, NY, USA) to show protein–ligand interaction. 

## 4. Conclusions

In summary, a pair of stereoisomers, neo-debromoaplysiatoxin E (**1**) and F (**2**), were isolated from the marine cyanobacterium *Lyngbya* sp. This is the first report on aplysiatoxin derivatives with different absolute configurations at C9–C12 (**1**: 9*S*, 10*R*, 11*S*, 12*S*; **2**: 9*R*, 10*S*, 11*R*, 12*R*) exhibiting potent potassium channel Kv1.5 blocking activities (IC_50_ = 1.22 ± 0.22 μM and 2.85 ± 0.29 μM, respectively). It has been reported that activation of protein kinase C (PKC) may cause inhibition of Kv1.5 ion channels [37], while some of the aplysiatoxins can activate PKC kinase [10,11,12,38]. The classical structures of ATXs (debromoaplysiatoxin, neo-debromoaplysiatoxin A) have a PKC kinase recognition region and a conformational control region [39], and may possess the PKC activation as well as Kv1.5 inhibition activities. However, some of the aplysiatoxins with distorted structures such as neo-debromoaplysiatoxin B and oscillatoxins, which are without these regions, do not possess PKC activation but retain Kv1.5 inhibition activities [10,11,12]. Thus, it was speculated that aplysiatoxin derivatives may inhibit Kv1.5 channel currents by two mechanisms: one was inhibiting Kv1.5 currents by activating PKC kinase, and the other was direct blocking. Our previous research has highlighted the potential of aplysiatoxins as ion channel blockers, specifically the selective blocking of Kv1.5 [10,11,12]. Therefore, the studies in search for the real mechanism of the differences is still ongoing.

## Figures and Tables

**Table 1:** ^1^H and ^13^C NMR data of **1** and **2** (*δ* in ppm).

Pos.	1		2	
	*^a^ δ*_H_ (*J* in Hz)	*^b^ δ*_C_	*^a^ δ*_H_ (*J* in Hz)	*^b^ δ*_C_
1		176.3, C		176.4, C
2	5.57, S	103.1, CH	5.66, S	104.7, CH
3		174.2, C		174.5, C
4		84.9, C		84.7, C
5	a 2.16, d (13.0) b 1.71, d (13.0)	47.1, CH_2_	a 2.14, d (13.0) b 1.78, d (13.0)	47.5, CH_2_
6		37.5, C		37.7, C
7		167.4, C		167.1, C
8		105.7, C		104.7, C
9	4.26, d (3.6)	64.5, CH	4.05, d (3.1)	66.8, CH
10	1.75, m	36.1, CH	1.88, m	35.6, CH
11	3.87, dd (11.1, 2.0)	79.0, CH	3.95, dd (11.4, 2.1)	79.4, CH
12	1.79, m	34.3, CH	1.75, m	34.1, CH
13	a 1.54, m b 1.46, m	31.4, CH_2_	a 1.56, m b 1.47, m	31.4, CH_2_
14	a 1.86, m b 1.73, m	36.7, CH_2_	a 1.83, m b 1.73, m	36.7, CH_2_
15	4.08, t (6.7)	85.7, CH	4.07, t (6.7)	85.7, CH
16		144.9, C		145.9, C
17	6.74, t (2.0)	114.3, CH	6.73, t (2.0)	114.3, CH
18		158.8, C		158.8, C
19	6.71, ddd (8.0, 2.5, 1.0)	115.6, CH	6.70, ddd (8.0, 2.5, 1.2)	115.6, CH
20	7.16, t (7.7)	130.4, CH	7.15, t (7.8)	130.4, CH
21	6.76, dt (7.7, 1.3)	119.2, CH	6.75, dt (7.7, 1.2)	119.1, CH
22	0.90, d (6.8)	13.0, CH_3_	0.89, d (6.8)	13.1, CH_3_
23	1.03, d (7.0)	11.8, CH_3_	0.99, d (6.9)	12.3, CH_3_
24	1.15, s	32.0, CH_3_	1.10, s	32.1, CH_3_
25	1.23, s	26.8, CH_3_	1.29, s	26.4, CH_3_
26	1.56, s	28.0, CH_3_	1.52, s	28.3, CH_3_
15-OCH_3_	3.21, s	56.8, CH_3_	3.21, s	56.8, CH_3_

*^a^* Measured at 600 MHz in MeOH-*d*_4_. *^b^* Measured at 15 MHz in MeOH-*d*_4_.

## Supplementary Materials

The following are available online at https://www.mdpi.com/1660-3397/17/12/652/s1, 1D and 2D NMR spectra, ECDs and DP4+ probability of compounds **1** and **2**.
