# Supplementary material for: Two New Neo-debromoaplysiatoxins—A Pair of Stereoisomers Exhibiting Potent Kv1.5 Ion Channel Inhibition Activities
Source: Mar Drugs. 2019 Nov 21;17(12):652. doi: 10.3390/md17120652 (PMC6950415; doi:10.3390/md17120652)

Supporting information

Two New Neo-debromoaplysiatoxins—A Pair of Stereoisomers Exhibiting Potent Kv1.5 Ion Channel Inhibition Activities

Ting-Ting Fan [1], Hui-Hui Zhang [1], Yang-Hua Tang [2], Fan-Zhong Zhang [1] and Bing-Nan Han [1,*]

[1] Department of Development Technology of Marine Resources, College of Life Sciences and Medicine, Zhejiang Sci-Tech University, Hangzhou 310018, China; tawnie1994@sina.com (T.-T.F.); 18895602876@sina.com (H.-H.Z.); fancyzfz@163.com (F.-Z.Z.)

[2] Department of Pharmacy, Graduate School, Hunan University of Chinese Medicine, Changsha 410208, China; tangyanghua@126.com (Y.-H.T.)

* Correspondence: hanbingnan@zstu.edu.cn; Tel: +86-571-8684-3303

# contents

1. Experimental details......3
1.1 Morphological and molecular identification of cyanobacterium......3
1.2 Bioassays......5
1.2.1 Ion channel experiment......5
1.3 Computational details......6
1.3.1 Computational methods for ECD of Neo-debromoaplysiatoxin E (1) and Neo-debromoaplysiatoxin F (2)......6
1.3.2 Methods for NMR calculation of Neo-debromoaplysiatoxin E (1) and Neo-debromoaplysiatoxin F (2)......42
2. Figures......80
Figure S1. ^{1}H NMR spectrum of compound **1** in MeOH-d_4. 80
Figure S2. ^{13}C NMR spectrum of compound **1** in MeOH-d_4. 81
Figure S3. DEPT spectrum of compound **1** in MeOH-d_4......81
Figure S4. HSQC spectrum of compound **1** in MeOH-d_4......82
Figure S5. ^{1}H-^{1}H COSY spectrum of compound **1** in MeOH-d_4. 82
Figure S6. HMBC spectrum of compound **1** in MeOH-d_4......83
Figure S7. NOESY spectrum of compound **1** in MeOH-d_4......83
Figure S8. HRESIMS spectrum of compound **1** in MeOH-d_4. 84
Figure S9. UV spectrum of Neo-debromoaplysiatoxin E (**1**) in MeOH......84
Figure S10. ^{1}H NMR spectrum of compound **2** in MeOH-d_4. 85
Figure S11. ^{13}C NMR spectrum of compound **2** in MeOH-d_4......85
Figure S12. DEPT spectrum of compound **2** in MeOH-d_4......86
Figure S13. HSQC spectrum of compound **2** in MeOH-d_4......86
Figure S14. ^{1}H-^{1}H COSY spectrum of compound **2** in MeOH-d_4. 87
Figure S15. HMBC spectrum of compound **2** in MeOH-d_4......87
Figure S16. NOESY spectrum of compound **2** in MeOH-d_4......88
Figure S17. HRESIMS spectrum of compound **2** in MeOH-d_4. 88
Figure S18. UV spectrum of Neo-debromoaplysiatoxin F (**2**) in MeOH. 89
Figure S19. Plausible Biosynthetic Pathway of **1** and **2**......90

1. Experimental details

1.1 Morphological and molecular identification of cyanobacterium

The cyanobacteria strains used in this study were collected from Harbor of Hainan Sanya, China, Named as cyanobacterium HN. Colonies of cyanobacteria HN appeared as dark red, brown, or black tufts ranging from 15 to 25 cm in length and grew attached to sea rock and surface of the sea.

Filament width, cell width, and cell length of cyanobacteria HN were measured on the compound light microscope (Zeiss, Oberkochen, Germany) with a 20x objective and 10x ocular lens with a calibrated optical micrometer. Filaments were long, of indeterminate length, 55-65 μm wide, formed by a uniseriate row of discoid cells encased in a firm, colorless, hyaline sheath which, when old, became yellowed and distinctly lamellated. Cells were discoid, 6-8 μm long, 30-40μm broad, with rounded end cells without calyptra. Cell contents were finely granular without prominent granular inclusions (Fig. S1 (A)).

16S rDNA was used to characterize the identity of cyanobacterium samples. Total cyanobacterium genomic DNA from lyophilized samples was extracted by using TianGen Plant Genomic DNA Kit (TIANGEN Biotech Co., Ltd., Beijing, China) according to the manufacturer's instructions. Three PCR primer sets, CYA106F (5′-TACGGCTACCTTGTTAACGCGTGA-3′) / 781R (5′-GACTACTGGGGTATC-TAATCCCATT-3′), 27F (5′-AGAGTTTGATCCTGGCTCAG-3′) /809R (5′-GC-TTCGGCACGGCTCGGGTCGATA-3′) and MSR2F (5′-CGGTAATACGGGG- GAGGCAA-3′) /2R(5′-CCAACATCTCACGACACGAG-3′), were used for amplifying 16S rDNA. PCR reactions were performed in a BIO RAD Cycler C1000, according to the following profile: 5 min at 95 °C and 35 cycles of 30 s at 95°C, 1 min at 58°C for CYA106F/781R, 30s at 50°C for27F/809R or MSR2F/2R, and 1 min at 72°C, followed by 10 min at 72°C. The products were analyzed by electrophoresis in 0.7% (w/v) agarose gels electrophoresis. 16S rDNA sequences of other cyanobacterial taxa were acquired from NCBI GenBank and EzBioCloud databases and aligned by using MUSCLE implemented in MEGA7.0, and the phylogenetic tree were reconstructed by MrBayes.

Cyanobacterium HN held the highest 16S rRNA gene similarity with *Lyngbya sp.*

CENA128^{T} with the value of 99%, revealing that cyanobacterium HN might belong to *Lyngbya sp.*. The phylogenetic trees based on the 16S rRNA gene sequences, reconstructed with the Bayesian MCMC methods, showed that cyanobacterium HN fell into the clade comprising *Lyngbya* species and formed a stable clade with *Lyngbya sp.* CENA128^{T} (Fig. S1(B)). According to these results, cyanobacteria HN belonged to *Lyngbya sp.*

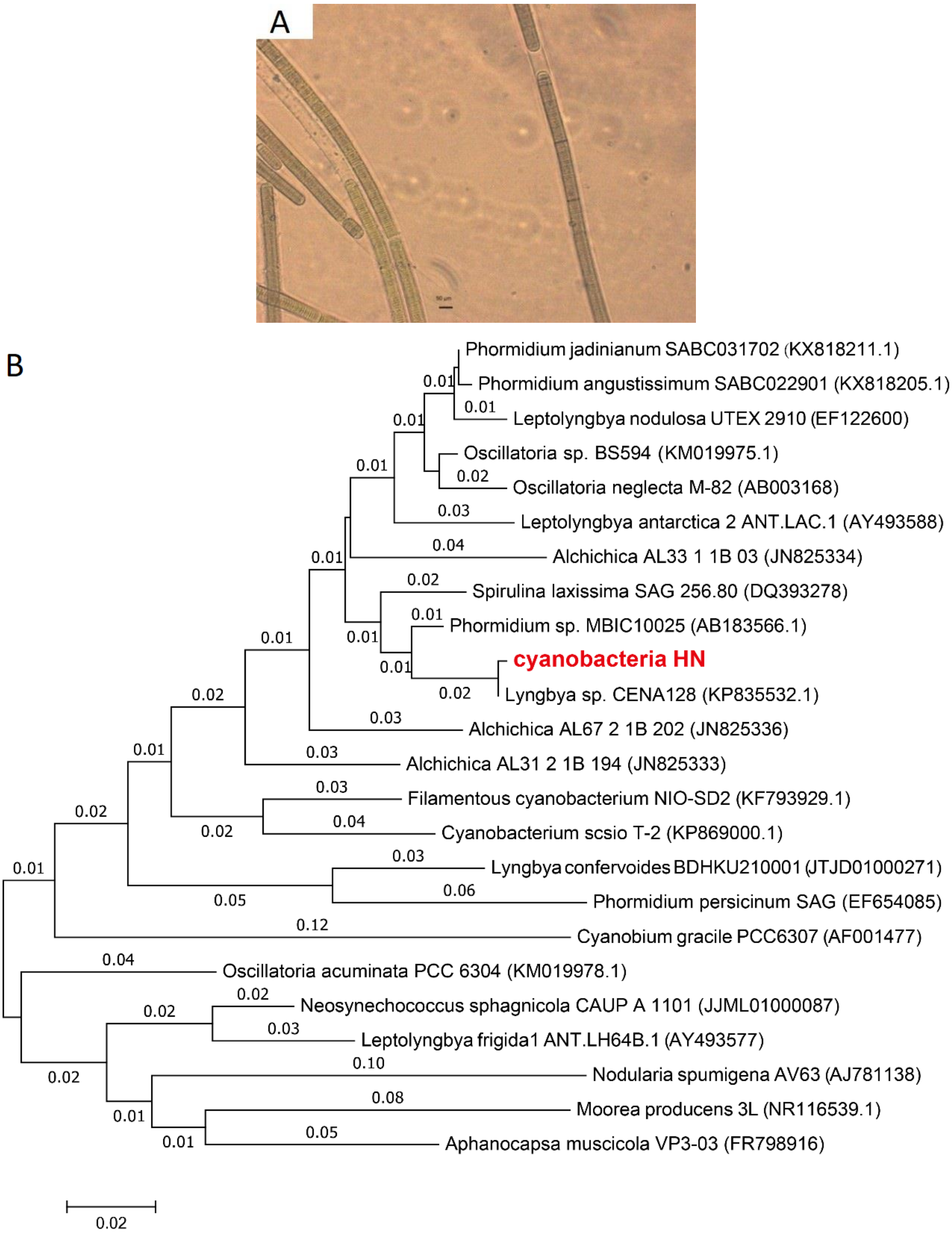

Fig. S1.1.1 (A) *Lyngbya* sp. collected in Harbor of Hainan. The specie, as identified based on morphological features, is shown in light micrographs. (B) Bayesian phylogenetic tree of *Lyngbya*

sp. HN and its close relatives. Bootstrap was set as 20,000 replicates. Bar, 0.02 substitutions per nucleotide position.

1.2 Bioassays

1.2.1 Ion channel experiment

Cell preparation. One day before the experiment, the density of 60%-80% CHO cells (Sigma Chemical Co., St. Louis, MO, USA) was digested with trypsin and placed in DMEM medium without P / S, added After 10% FBS, it was cultured overnight in an incubator.

Electrophysiology. The cells were transferred to a perfusion tank and perfused with extracellular fluid. The intracellular fluid (mM) was: K Aspartate, 130; $MgCl_2$, 5; EGTA 5; HEPES, 10; Tris-ATP 4; pH 7.2 (KOH titration). The intracellular fluid was stored in small portions in a refrigerator at -80°C and thawed on the day of the experiment. The electrodes were filled with intracellular fluid and drawn with PC-10 (Narishige, Tokyo, Japan). Whole-cell patch clamp recording, noise is filtered using one-fifth of the sampling frequency. The cells were clamped at –80 mV and then depolarized to 20 mV with a square wave lasting 2 seconds to obtain Kv1.5 current. This procedure is repeated every 20 seconds. After it was stabilized, compound **1**, compound **2** and acacetin were perfused, and when the reaction was stabilized, the strength of the blocking was calculated.

Data analysis and statistics. Data acquisition and analysis were carried out using pCLAMP 10 (Molecular Devices, Union City, CA). Data fitting and statistical analyses were performed using ORIGIN 8.0 (GraphPadSoftware Inc., San Diego, CA). IC_{50} value was determined by fitting the data points to the equation. Where IC_{50} is the concentration at which half-maximal currents were inhibited, all the data were presented as mean ±SEM.

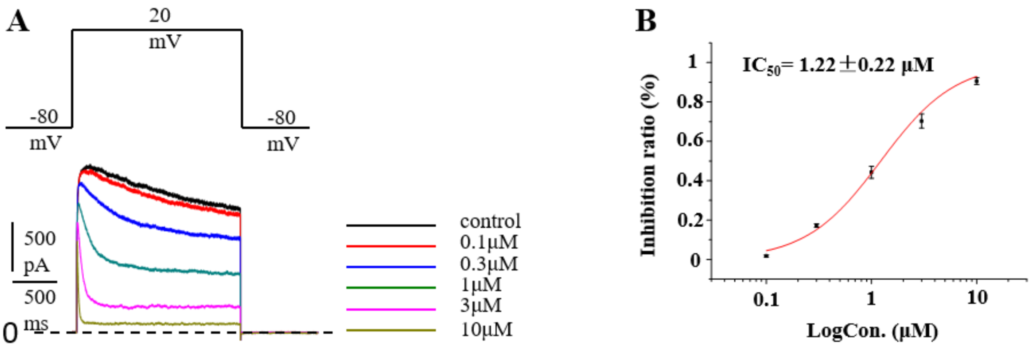

Fig. S1.2.1.1 (A) Kv1.5 traces elicited by 5 s pulses from -80 to +20 mV and tail currents recorded at -80 mV in the absence and presence of 0.1 μM, 0.3 μM, 1 μM, 3 μM, 10 μM and 30 μM neo-debromoaplysiatoxin E (1). (B) Percent blocked-Concentration curves. The abscissa represents the

concentration, and the ordinate represents the percentage of Kv1.5 current that is blocked at different concentrations of neo-debromoaplysiatoxin E (1). Data points represent mean ±SEM of 3 to 5 measurements, and inhibitory effect showed IC_{50} value of 2.85±0.29 μM.

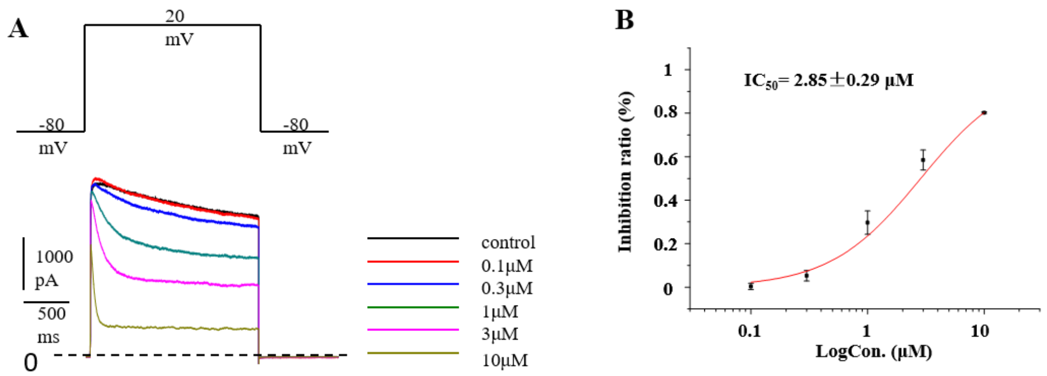

Fig. S1.2.1.2. (A) Kv1.5 traces elicited by 5 s pulses from -80 to +20 mV and tail currents recorded at -80 mV in the absence and presence of 0.1 μM, 0.3 μM, 1 μM, 3 μM, 10 μM and 30 μM neo-debromoaplysiatoxin F (2). (B) Percent blocked-Concentration curves. The abscissa represents the concentration, and the ordinate represents the percentage of Kv1.5 current that is blocked at different concentrations of neo-debromoaplysiatoxin F (2). Data points represent mean ±SEM of 3 to 5 measurements, and inhibitory effect showed IC_{50} value of 2.85±0.29 μM.

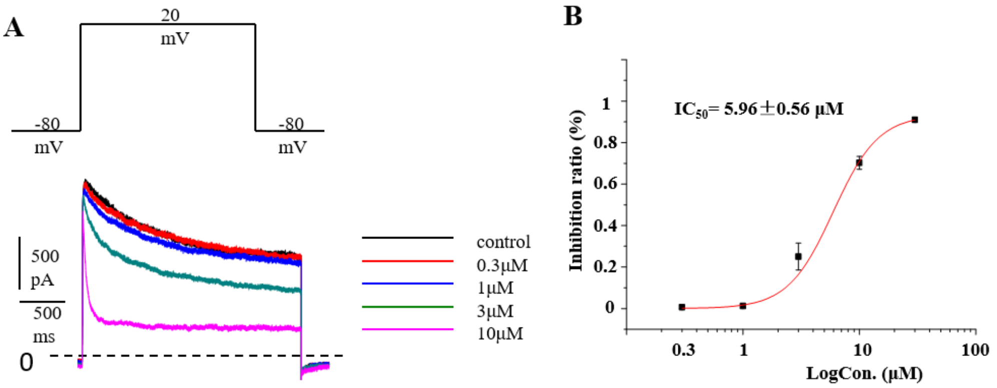

Fig. S1.2.1.3. (A) Kv1.5 traces elicited by 5 s pulses from -80 to +20 mV and tail currents recorded at -80 mV in the absence and presence of 0.1 μM, 0.3 μM, 1 μM, 3 μM, 10 μM and 30 μM acacetin. (B) Percent blocked-Concentration curves. The abscissa represents the concentration, and the ordinate represents the percentage of Kv1.5 current that is blocked at different concentrations of acacetin . Data points represent mean ±SEM of 3 to 5 measurements, and inhibitory effect showed IC_{50} value of 5.96±0.56 μM.

1.3 Computational details

1.3.1 Computational methods for ECD of Neo-debromoaplysiatoxin E (1) and Neo-debromoaplysiatoxin F (2)

Monte Carlo conformational searches were carried out by means of the Spartan's 10 software (Spartan Software, San Francisco, CA, USA) using Merck Molecular Force Field (MMFF). The

conformers with Boltzmann-population of over 5% were chosen for ECD calculations, and then the conformers were initially optimized at B3LYP/6-31+g (d, p) level in MeOH using the CPCM polarizable conductor calculation model. The theoretical calculation of ECD was conducted in MeOH using Time-dependent Density functional theory (TD-DFT) at the B3LYP/6-31+g (d, p) level for all conformers of **isomer 1, isomer 1'**, **isomer 2, isomer 2'**. Rotatory strengths for a total of 30 excited states were calculated. ECD spectra were generated using the program SpecDis 1.6 (University of Würzburg, Würzburg, Germany) and GraphPad Prism 5 (University of California San Diego, San Diego, CA, USA) from dipole-length rotational strengths by applying Gaussian band shapes with sigma = 0.3 eV.

Table S1.3.1.1.a. Gibbs free energies[a] and equilibrium populations[b] of low-energy conformers of **isomer 1**.

Conformers	In MeOH	
	ΔG	P (%)/100
**Isomer 1.-1**	0.00	0.388
**Isomer 1.-2**	0.72	0.115
**Isomer 1.-3**	1.09	0.062
**Isomer 1.-4**	1.21	0.050
**Isomer 1.-5**	1.21	0.050

[a]B3LYP/6-31+G (d,p), in kcal/mol. [b]From ΔG values at 298.15K.

Table S1.3.1.1.b. Cartesian coordinates for the low-energy reoptimized MMFF conformers of **isomer 1** at B3LYP/6-311+G (d,p) level of theory in CH_3OH.

**isomer 1.-1**		Standard Orientation (Ångstroms)			
Center number	Atomic number	Atomic Type	X	Y	Z
1.	6.	0.	1.990680	2.592915	-0.580915
2.	6.	0.	1.829990	2.946698	0.765501
3.	6.	0.	2.331808	2.105315	1.761579
4.	6.	0.	2.978336	0.912551	1.438862
5.	6.	0.	3.128594	0.546849	0.092135
6.	6.	0.	2.647693	1.396200	-0.905209
7.	6.	0.	3.768964	-0.784969	-0.281477
8.	8.	0.	1.539941	3.359536	-1.609134
9.	6.	0.	2.925145	-2.007460	0.125187
10.	8.	0.	5.031825	-0.980057	0.351808
11.	6.	0.	6.045167	-0.100238	-0.101367
12.	6.	0.	1.471831	-1.941105	-0.362577
13.	6.	0.	0.661476	-3.248769	-0.210874

14.	6.	0.	0.873344	-3.929353	1.152718
15.	6.	0.	-0.857771	-3.105498	-0.489861
16.	6.	0.	-1.308942	-2.559461	-1.857103
17.	6.	0.	-1.208282	-1.013654	-1.924443
18.	6.	0.	-2.730650	-3.048968	-2.181673
19.	6.	0.	-1.876678	1.056209	-0.580318
20.	6.	0.	-1.293392	2.050717	-1.284048
21.	6.	0.	-1.810026	-1.086763	0.514319
22.	6.	0.	-2.228378	-0.523139	1.875507
23.	6.	0.	-2.251508	1.034453	1.864539
24.	6.	0.	-2.687494	1.666951	0.539108
25.	6.	0.	-1.462189	3.292665	-0.531393
26.	8.	0.	-0.921432	4.376331	-0.689778
27.	8.	0.	-1.507145	-2.398235	0.609773
28.	6.	0.	-4.194172	1.624467	0.255045
29.	6.	0.	-1.202667	-0.953905	2.951982
30.	6.	0.	-1.717353	-0.377870	-0.648460
31.	6.	0.	-3.601372	-1.126658	2.265921
32.	8.	0.	-2.300315	3.065617	0.530900
33.	8.	0.	-1.852884	-0.486026	-3.083828
34.	1.	0.	1.320668	3.871043	1.020898
35.	1.	0.	2.219010	2.393515	2.803458
36.	1.	0.	3.379636	0.272393	2.218353
37.	1.	0.	2.761498	1.141374	-1.955412
38.	1.	0.	3.904705	-0.799969	-1.377239
39.	1.	0.	0.825408	3.955970	-1.301508
40.	1.	0.	3.430165	-2.898399	-0.268405
41.	1.	0.	2.961372	-2.082931	1.217067
42.	1.	0.	6.958497	-0.365441	0.436480
43.	1.	0.	6.224106	-0.212737	-1.182103
44.	1.	0.	5.801984	0.950866	0.102056
45.	1.	0.	1.480733	-1.656869	-1.421076
46.	1.	0.	0.966533	-1.129822	0.170972
47.	1.	0.	1.013027	-3.950921	-0.982795
48.	1.	0.	1.913503	-4.238371	1.278907
49.	1.	0.	0.617654	-3.258359	1.976910
50.	1.	0.	0.248714	-4.824443	1.245551
51.	1.	0.	-1.270227	-4.115586	-0.391845
52.	1.	0.	-0.630892	-2.969892	-2.615256
53.	1.	0.	-0.163138	-0.726048	-2.067430
54.	1.	0.	-3.458162	-2.694697	-1.442964
55.	1.	0.	-3.042180	-2.704392	-3.170287
56.	1.	0.	-2.769013	-4.142549	-2.183812

57.	1.	0.	-0.647135	1.979936	-2.145316
58.	1.	0.	-2.878456	1.394402	2.687744
59.	1.	0.	-1.236091	1.398513	2.053091
60.	1.	0.	-4.542258	0.602119	0.093467
61.	1.	0.	-4.749141	2.058031	1.091743
62.	1.	0.	-4.410410	2.206389	-0.644479
63.	1.	0.	-0.190565	-0.632211	2.687306
64.	1.	0.	-1.464034	-0.487703	3.907854
65.	1.	0.	-1.200200	-2.036636	3.088818
66.	1.	0.	-3.544302	-2.218290	2.273290
67.	1.	0.	-4.399396	-0.835736	1.579963
68.	1.	0.	-3.881479	-0.793090	3.270371
69.	1.	0.	-2.802982	-0.455858	-2.908416

**isomer 1.-2**		Standard Orientation (Ångstroms)			
Center number	Atom number	Type	X	Y	Z
1.	6.	0.	2.005060	2.587236	-0.576060
2.	6.	0.	1.850072	2.942759	0.770653
3.	6.	0.	2.352890	2.101224	1.765818
4.	6.	0.	2.994787	0.906050	1.442517
5.	6.	0.	3.139597	0.538440	0.095936
6.	6.	0.	2.658406	1.388633	-0.900725
7.	6.	0.	3.772179	-0.796834	-0.278172
8.	8.	0.	1.551498	3.354009	-1.602944
9.	6.	0.	2.922310	-2.014434	0.130785
10.	8.	0.	5.035371	-0.999064	0.351849
11.	6.	0.	6.051810	-0.122825	-0.101082
12.	6.	0.	1.468558	-1.941567	-0.354976
13.	6.	0.	0.653677	-3.246618	-0.206005
14.	6.	0.	0.861350	-3.927022	1.158380
15.	6.	0.	-0.865376	-3.100293	-0.485572
16.	6.	0.	-1.322671	-2.554604	-1.851903
17.	6.	0.	-1.215745	-1.009606	-1.919987
18.	6.	0.	-2.751771	-3.035251	-2.156930
19.	6.	0.	-1.878285	1.061042	-0.584651
20.	6.	0.	-1.276386	2.053165	-1.274825
21.	6.	0.	-1.820007	-1.080028	0.510639
22.	6.	0.	-2.249029	-0.516601	1.867773
23.	6.	0.	-2.273772	1.040758	1.856447
24.	6.	0.	-2.696416	1.674339	0.526811
25.	6.	0.	-1.445697	3.293575	-0.522878

26.	8.	0.	-0.892394	4.373571	-0.672440
27.	8.	0.	-1.515073	-2.393121	0.610605
28.	6.	0.	-4.199469	1.637566	0.226548
29.	6.	0.	-1.234567	-0.947186	2.954647
30.	6.	0.	-1.724120	-0.372848	-0.650514
31.	6.	0.	-3.626611	-1.119664	2.242540
32.	8.	0.	-2.300574	3.072196	0.525416
33.	8.	0.	-1.966495	-0.467454	-3.011316
34.	1.	0.	1.342425	3.867813	1.026445
35.	1.	0.	2.243527	2.390418	2.807751
36.	1.	0.	3.395607	0.265008	2.221509
37.	1.	0.	2.769752	1.132524	-1.950990
38.	1.	0.	3.905481	-0.813092	-1.374458
39.	1.	0.	0.832888	3.946248	-1.292876
40.	1.	0.	3.422269	-2.908580	-0.262137
41.	1.	0.	2.959263	-2.088796	1.222653
42.	1.	0.	6.964870	-0.393330	0.434553
43.	1.	0.	6.228488	-0.233778	-1.182434
44.	1.	0.	5.813667	0.928879	0.105021
45.	1.	0.	1.477792	-1.654436	-1.413020
46.	1.	0.	0.966083	-1.129693	0.180281
47.	1.	0.	1.005249	-3.950028	-0.977056
48.	1.	0.	1.900793	-4.237116	1.287985
49.	1.	0.	0.603304	-3.255068	1.981071
50.	1.	0.	0.235329	-4.821226	1.249768
51.	1.	0.	-1.278501	-4.110485	-0.390776
52.	1.	0.	-0.648060	-2.976647	-2.609832
53.	1.	0.	-0.164329	-0.722468	-2.050830
54.	1.	0.	-3.449073	-2.719304	-1.375111
55.	1.	0.	-3.108677	-2.624608	-3.102091
56.	1.	0.	-2.781663	-4.127970	-2.214680
57.	1.	0.	-0.617138	1.983113	-2.125799
58.	1.	0.	-2.909718	1.399913	2.673105
59.	1.	0.	-1.260734	1.405558	2.056164
60.	1.	0.	-4.547142	0.616941	0.055833
61.	1.	0.	-4.761408	2.071399	1.058598
62.	1.	0.	-4.402947	2.219449	-0.675535
63.	1.	0.	-0.219874	-0.624708	2.700627
64.	1.	0.	-1.505772	-0.481945	3.908271
65.	1.	0.	-1.232553	-2.030065	3.090807
66.	1.	0.	-3.570164	-2.211339	2.251180
67.	1.	0.	-4.415041	-0.828597	1.545834
68.	1.	0.	-3.918754	-0.785278	3.243370

69.	1.	0.	-1.478552	-0.656223	-3.823846

**isomer 1.-3**		Standard Orientation (Ångstroms)			
Center number	Atom number	Type	X	Y	Z
1.	6.	0.	-7.646926	-0.372959	-0.054795
2.	6.	0.	-8.152552	0.932276	-0.055917
3.	6.	0.	-7.299181	1.992133	0.235974
4.	6.	0.	-5.951839	1.766137	0.529010
5.	6.	0.	-5.444351	0.463756	0.521151
6.	6.	0.	-6.298974	-0.605084	0.229959
7.	6.	0.	-3.964651	0.203262	0.773046
8.	8.	0.	-8.521085	-1.384564	-0.339807
9.	6.	0.	-3.172038	0.125872	-0.541497
10.	8.	0.	-3.739634	-1.024724	1.461137
11.	6.	0.	-4.099570	-0.991767	2.832057
12.	6.	0.	-1.660103	-0.011936	-0.319506
13.	6.	0.	-0.854824	-0.273770	-1.608143
14.	6.	0.	-0.977751	0.870376	-2.628832
15.	6.	0.	0.625534	-0.581539	-1.323403
16.	6.	0.	0.948422	-1.778097	-0.420496
17.	6.	0.	2.483869	-1.934651	-0.368227
18.	6.	0.	0.268055	-3.078673	-0.858712
19.	6.	0.	4.528410	-0.538633	0.307736
20.	6.	0.	5.328781	-1.419035	0.940771
21.	6.	0.	2.468623	0.570621	-0.287594
22.	6.	0.	2.969793	1.956699	0.122549
23.	6.	0.	4.406737	1.889273	0.724871
24.	6.	0.	5.278266	0.766766	0.150277
25.	6.	0.	6.556350	-0.725052	1.365984
26.	8.	0.	7.502282	-1.130238	1.996202
27.	8.	0.	1.200050	0.622587	-0.746066
28.	6.	0.	5.748089	0.992680	-1.294406
29.	6.	0.	2.017825	2.503076	1.217440
30.	6.	0.	3.156892	-0.601111	-0.146675
31.	6.	0.	2.904089	2.922222	-1.086358
32.	8.	0.	6.465700	0.596905	0.949378
33.	8.	0.	3.001824	-2.611509	-1.522153
34.	1.	0.	-9.202325	1.090497	-0.278380
35.	1.	0.	-7.690904	3.005251	0.243578
36.	1.	0.	-5.297381	2.601117	0.764725
37.	1.	0.	-5.902970	-1.618065	0.242918

38.	1.	0.	-3.562683	1.035365	1.376848
39.	1.	0.	-8.048515	-2.226788	-0.307406
40.	1.	0.	-3.551461	-0.731304	-1.113309
41.	1.	0.	-3.405626	1.021697	-1.126186
42.	1.	0.	-5.171954	-0.802257	2.972666
43.	1.	0.	-3.852994	-1.970805	3.249476
44.	1.	0.	-3.534516	-0.220402	3.376796
45.	1.	0.	-1.504004	-0.832443	0.387030
46.	1.	0.	-1.275204	0.895362	0.163580
47.	1.	0.	-1.256050	-1.180721	-2.080428
48.	1.	0.	-2.011479	0.998401	-2.960376
49.	1.	0.	-0.639291	1.818449	-2.200217
50.	1.	0.	-0.371373	0.669819	-3.518995
51.	1.	0.	1.132683	-0.740814	-2.289625
52.	1.	0.	0.621039	-1.527025	0.597194
53.	1.	0.	2.725820	-2.598712	0.470815
54.	1.	0.	0.518770	-3.326048	-1.893447
55.	1.	0.	0.611258	-3.910617	-0.235936
56.	1.	0.	-0.818864	-3.017124	-0.761593
57.	1.	0.	5.134580	-2.450283	1.200446
58.	1.	0.	4.899522	2.859655	0.601027
59.	1.	0.	4.337712	1.703789	1.801843
60.	1.	0.	4.910542	0.995796	-1.995321
61.	1.	0.	6.278315	1.946083	-1.372438
62.	1.	0.	6.433445	0.191316	-1.581519
63.	1.	0.	2.383569	3.472714	1.571043
64.	1.	0.	1.005376	2.637152	0.830141
65.	1.	0.	1.972872	1.824647	2.075106
66.	1.	0.	3.180256	3.931596	-0.765239
67.	1.	0.	1.890976	2.958247	-1.493088
68.	1.	0.	3.581264	2.631893	-1.892475
69.	1.	0.	3.204640	-1.940161	-2.186355

**isomer 1.-4**		Standard Orientation (Ångstroms)			
Center number	Atom number	Type	X	Y	Z
1.	6.	0.	-5.231067	-2.457850	0.245670
2.	6.	0.	-4.776650	-3.301851	-0.774133
3.	6.	0.	-3.985906	-2.774143	-1.790756
4.	6.	0.	-3.645726	-1.419105	-1.802452
5.	6.	0.	-4.091325	-0.577551	-0.778771
6.	6.	0.	-4.887549	-1.103447	0.244250

7.	6.	0.	-3.672702	0.886956	-0.737768
8.	8.	0.	-6.010843	-3.014858	1.220950
9.	6.	0.	-2.409122	1.084471	0.114635
10.	8.	0.	-4.683405	1.724386	-0.181758
11.	6.	0.	-5.792155	1.936624	-1.038757
12.	6.	0.	-1.889907	2.528343	0.076966
13.	6.	0.	-0.699857	2.876603	1.002894
14.	6.	0.	-1.017226	2.636684	2.488377
15.	6.	0.	0.656883	2.259425	0.625679
16.	6.	0.	1.152465	2.466114	-0.812797
17.	6.	0.	2.571840	1.884781	-0.904216
18.	6.	0.	1.120024	3.928469	-1.275449
19.	6.	0.	3.777829	-0.337818	-0.503690
20.	6.	0.	4.670599	-0.412438	-1.514283
21.	6.	0.	1.657301	0.071588	0.572287
22.	6.	0.	1.548696	-1.346368	1.137006
23.	6.	0.	2.748665	-2.234206	0.682265
24.	6.	0.	4.057135	-1.465858	0.466112
25.	6.	0.	5.461792	-1.645356	-1.359779
26.	8.	0.	6.325020	-2.108673	-2.064553
27.	8.	0.	0.592886	0.829239	0.908222
28.	6.	0.	4.733184	-0.969063	1.752615
29.	6.	0.	0.249204	-1.992870	0.594871
30.	6.	0.	2.654022	0.528899	-0.240174
31.	6.	0.	1.439302	-1.287282	2.680893
32.	8.	0.	5.020763	-2.294786	-0.215406
33.	8.	0.	3.467108	2.828176	-0.299376
34.	1.	0.	-5.056065	-4.349759	-0.754986
35.	1.	0.	-3.638631	-3.425042	-2.588063
36.	1.	0.	-3.036471	-1.017068	-2.607669
37.	1.	0.	-5.246140	-0.440885	1.028844
38.	1.	0.	-3.458808	1.215187	-1.770189
39.	1.	0.	-6.256745	-2.328964	1.855609
40.	1.	0.	-2.651772	0.786676	1.140952
41.	1.	0.	-1.645155	0.387856	-0.239828
42.	1.	0.	-6.476157	2.608189	-0.514472
43.	1.	0.	-5.484453	2.409651	-1.983594
44.	1.	0.	-6.318375	1.001646	-1.273156
45.	1.	0.	-2.720052	3.189942	0.346524
46.	1.	0.	-1.630087	2.781528	-0.959424
47.	1.	0.	-0.540396	3.957264	0.887864
48.	1.	0.	-1.948767	3.139127	2.767621
49.	1.	0.	-1.125395	1.573456	2.714664

50.	1.	0.	-0.218995	3.031245	3.126232
51.	1.	0.	1.421563	2.671625	1.299557
52.	1.	0.	0.518980	1.864595	-1.478261
53.	1.	0.	2.831044	1.769537	-1.969084
54.	1.	0.	1.682236	4.570212	-0.593246
55.	1.	0.	1.581720	4.019333	-2.263654
56.	1.	0.	0.097180	4.305810	-1.351697
57.	1.	0.	4.773898	0.223893	-2.382782
58.	1.	0.	2.894892	-3.044750	1.404276
59.	1.	0.	2.505457	-2.703816	-0.276679
60.	1.	0.	4.133263	-0.207167	2.254369
61.	1.	0.	4.894754	-1.804577	2.439389
62.	1.	0.	5.705165	-0.533982	1.505857
63.	1.	0.	0.234578	-1.995031	-0.499584
64.	1.	0.	0.188312	-3.032147	0.934312
65.	1.	0.	-0.638086	-1.465745	0.950554
66.	1.	0.	0.596138	-0.660703	2.980088
67.	1.	0.	2.340456	-0.887750	3.150928
68.	1.	0.	1.273509	-2.294707	3.075650
69.	1.	0.	4.326675	2.394961	-0.210545

**isomer 1.-5**		Standard Orientation (Ångstroms)			
Center number	Atom number	Type	X	Y	Z
1.	6.	0.	-3.325234	2.894862	-0.192937
2.	6.	0.	-4.166009	3.124375	0.902960
3.	6.	0.	-4.932236	2.075903	1.409454
4.	6.	0.	-4.865476	0.806113	0.834969
5.	6.	0.	-4.016460	0.572152	-0.254212
6.	6.	0.	-3.251344	1.623413	-0.767634
7.	6.	0.	-3.894510	-0.815768	-0.872555
8.	8.	0.	-2.552471	3.877319	-0.746076
9.	6.	0.	-2.590734	-1.530593	-0.481470
10.	8.	0.	-3.887314	-0.769067	-2.297489
11.	6.	0.	-5.127752	-0.397087	-2.871220
12.	6.	0.	-2.480680	-1.835603	1.017789
13.	6.	0.	-1.203398	-2.573422	1.488417
14.	6.	0.	-1.023325	-3.941338	0.810500
15.	6.	0.	0.099312	-1.756309	1.440673
16.	6.	0.	0.083507	-0.369624	2.100933
17.	6.	0.	1.511746	0.194839	2.039068
18.	6.	0.	-0.446718	-0.379156	3.540910

19.	6.	0.	3.308821	0.725012	0.295548
20.	6.	0.	3.854331	1.882399	0.727938
21.	6.	0.	1.568660	-0.857807	-0.239577
22.	6.	0.	2.000604	-1.002938	-1.700050
23.	6.	0.	3.198270	-0.061123	-2.035889
24.	6.	0.	4.141477	0.205477	-0.857117
25.	6.	0.	4.945239	2.264563	-0.185078
26.	8.	0.	5.653477	3.241960	-0.184285
27.	8.	0.	0.492009	-1.619491	0.043012
28.	6.	0.	5.013909	-0.989652	-0.446207
29.	6.	0.	0.810641	-0.597536	-2.605889
30.	6.	0.	2.129327	-0.014659	0.675328
31.	6.	0.	2.341603	-2.483983	-1.998598
32.	8.	0.	5.041610	1.288722	-1.167117
33.	8.	0.	2.277647	-0.443864	3.070731
34.	1.	0.	-4.224069	4.115280	1.348330
35.	1.	0.	-5.591126	2.256095	2.253962
36.	1.	0.	-5.474806	-0.002331	1.229249
37.	1.	0.	-2.610019	1.467522	-1.628612
38.	1.	0.	-4.752352	-1.422286	-0.532275
39.	1.	0.	-2.700714	4.704438	-0.269140
40.	1.	0.	-2.550034	-2.454937	-1.067271
41.	1.	0.	-1.751759	-0.911840	-0.809519
42.	1.	0.	-5.001498	-0.442493	-3.955466
43.	1.	0.	-5.931216	-1.090505	-2.577908
44.	1.	0.	-5.427551	0.621259	-2.589892
45.	1.	0.	-3.337129	-2.457878	1.313486
46.	1.	0.	-2.586157	-0.902845	1.581860
47.	1.	0.	-1.351071	-2.769260	2.558961
48.	1.	0.	-1.926501	-4.549106	0.927582
49.	1.	0.	-0.818802	-3.842096	-0.257987
50.	1.	0.	-0.189601	-4.492667	1.258487
51.	1.	0.	0.891319	-2.344997	1.925524
52.	1.	0.	-0.547204	0.289235	1.489735
53.	1.	0.	1.463774	1.277334	2.239596
54.	1.	0.	0.118105	-1.076368	4.164164
55.	1.	0.	-0.341684	0.615449	3.985505
56.	1.	0.	-1.505054	-0.648766	3.579404
57.	1.	0.	3.530802	2.531734	1.530010
58.	1.	0.	3.753771	-0.469820	-2.886677
59.	1.	0.	2.811799	0.914750	-2.347806
60.	1.	0.	4.412861	-1.806366	-0.041432
61.	1.	0.	5.579811	-1.356559	-1.307081

62.	1.	0.	5.723685	-0.671736	0.321951
63.	1.	0.	0.477786	0.421934	-2.386710
64.	1.	0.	1.120945	-0.632722	-3.655536
65.	1.	0.	-0.037850	-1.272615	-2.477942
66.	1.	0.	1.490712	-3.126841	-1.762390
67.	1.	0.	3.202053	-2.840122	-1.427867
68.	1.	0.	2.573035	-2.600555	-3.062205
69.	1.	0.	3.204637	-0.209035	2.929389

Table S1.3.1.2.a. Gibbs free energies[a] and equilibrium populations[b] of low-energy conformers of **isomer 2**.

Conformers	In MeOH	
	ΔG	P (%)/100
**isomer 2.-1**	0.00	0.146
**isomer 2.-2**	0.08	0.129
**isomer 2.-3**	0.12	0.120
**isomer 2.-4**	0.19	0.107
**isomer 2.-5**	0.31	0.086

[a]B3LYP/6-31+G (d,p), in kcal/mol. [b]From ΔG values at 298.15K.

Table S1.3.1.2.b. Cartesian coordinates for the low-energy reoptimized MMFF conformers of **isomer 2** at B3LYP/6-311+G (d,p) level of theory in CH_3OH.

**Isomer 2.-1**		Standard Orientation (Ångstroms)			
Center number	Atomic number	Atomic Type	X	Y	Z
1.	6.	0.	4.152563	-2.795227	-0.472390
2.	6.	0.	5.225298	-2.939514	0.415064
3.	6.	0.	5.794252	-1.803823	0.983701
4.	6.	0.	5.308314	-0.530066	0.677741
5.	6.	0.	4.230378	-0.385864	-0.200450
6.	6.	0.	3.655802	-1.524918	-0.775194
7.	6.	0.	3.643898	0.989128	-0.493179
8.	8.	0.	3.629277	-3.935961	-1.014708
9.	6.	0.	2.458793	1.307442	0.431169
10.	8.	0.	3.156982	1.096599	-1.830261
11.	6.	0.	4.178499	1.182509	-2.809675
12.	6.	0.	1.945620	2.743232	0.247729
13.	6.	0.	0.692641	3.154592	1.054945
14.	6.	0.	0.909687	3.067310	2.574432
15.	6.	0.	-0.620217	2.463763	0.640241
16.	6.	0.	-0.962484	2.455262	-0.854558
17.	6.	0.	-2.334539	1.772174	-1.045713
18.	6.	0.	-0.957198	3.853526	-1.483580

19.	6.	0.	-3.467724	-0.466761	-0.517451
20.	6.	0.	-4.696967	-0.356938	-1.055746
21.	6.	0.	-1.574941	0.250082	0.795453
22.	6.	0.	-1.625285	-0.969575	1.719596
23.	6.	0.	-2.831861	-1.901935	1.382426
24.	6.	0.	-3.245549	-1.900835	-0.094013
25.	6.	0.	-5.396033	-1.645924	-0.901885
26.	8.	0.	-6.518179	-1.965778	-1.208798
27.	8.	0.	-0.590773	1.104854	1.159073
28.	6.	0.	-2.276285	-2.636064	-1.031300
29.	6.	0.	-1.809930	-0.468754	3.174202
30.	6.	0.	-2.426353	0.500424	-0.238732
31.	6.	0.	-0.279755	-1.733258	1.647661
32.	8.	0.	-4.535321	-2.524502	-0.255200
33.	8.	0.	-3.436636	2.652372	-0.768585
34.	1.	0.	5.596658	-3.934506	0.635565
35.	1.	0.	6.632432	-1.912468	1.666031
36.	1.	0.	5.768008	0.349662	1.120278
37.	1.	0.	2.829620	-1.405996	-1.472711
38.	1.	0.	4.432494	1.745083	-0.334243
39.	1.	0.	2.910900	-3.697775	-1.615404
40.	1.	0.	1.666242	0.582605	0.224835
41.	1.	0.	2.778347	1.135849	1.464888
42.	1.	0.	4.820785	2.060376	-2.643194
43.	1.	0.	3.683643	1.285817	-3.778221
44.	1.	0.	4.811709	0.285542	-2.824859
45.	1.	0.	1.765353	2.905172	-0.818835
46.	1.	0.	2.750914	3.438522	0.523897
47.	1.	0.	0.521882	4.214369	0.821882
48.	1.	0.	0.047576	3.467796	3.119022
49.	1.	0.	1.788787	3.649998	2.868262
50.	1.	0.	1.059882	2.037079	2.905172
51.	1.	0.	-1.441366	2.972173	1.173769
52.	1.	0.	-0.220501	1.827600	-1.366348
53.	1.	0.	-2.448235	1.530971	-2.108986
54.	1.	0.	-1.285558	3.796363	-2.526026
55.	1.	0.	-1.650508	4.520214	-0.964385
56.	1.	0.	0.039292	4.301462	-1.473427
57.	1.	0.	-5.177038	0.527379	-1.450109
58.	1.	0.	-2.608322	-2.920332	1.718184
59.	1.	0.	-3.709273	-1.567929	1.946139
60.	1.	0.	-1.309137	-2.131238	-1.084165
61.	1.	0.	-2.701103	-2.669523	-2.037694

62.	1.	0.	-2.123970	-3.662312	-0.684900
63.	1.	0.	-0.960667	0.137301	3.496304
64.	1.	0.	-2.719961	0.132221	3.271663
65.	1.	0.	-1.899740	-1.325793	3.849754
66.	1.	0.	-0.287576	-2.560868	2.364039
67.	1.	0.	-0.088656	-2.150827	0.656644
68.	1.	0.	0.552623	-1.073116	1.901173
69.	1.	0.	-3.612081	2.608535	0.180863

**Isomer 2.-2**		Standard Orientation (Ångstroms)			
Center number	Atom number	Type	X	Y	Z
1.	6.	0.	4.218037	2.664970	-1.023312
2.	6.	0.	5.094436	3.090124	-0.021494
3.	6.	0.	5.745855	2.139729	0.767575
4.	6.	0.	5.524830	0.778842	0.571928
5.	6.	0.	4.641754	0.350798	-0.430997
6.	6.	0.	3.998928	1.297666	-1.229216
7.	6.	0.	4.371120	-1.136782	-0.622504
8.	8.	0.	3.548594	3.533447	-1.840146
9.	6.	0.	3.440717	-1.720133	0.454507
10.	8.	0.	5.566289	-1.909960	-0.552809
11.	6.	0.	6.441599	-1.721404	-1.650724
12.	6.	0.	2.012721	-1.163222	0.408599
13.	6.	0.	1.118025	-1.630261	1.575367
14.	6.	0.	0.977577	-3.160369	1.645680
15.	6.	0.	-0.269745	-0.964412	1.551856
16.	6.	0.	-0.323057	0.567035	1.593458
17.	6.	0.	-1.804249	1.005082	1.606661
18.	6.	0.	0.445595	1.168721	2.774644
19.	6.	0.	-3.882215	0.754232	0.121506
20.	6.	0.	-4.829039	1.492220	0.731290
21.	6.	0.	-2.123507	-0.893635	0.003763
22.	6.	0.	-2.854705	-1.747957	-1.034923
23.	6.	0.	-4.267560	-1.172447	-1.366204
24.	6.	0.	-4.381052	0.352067	-1.247326
25.	6.	0.	-6.034217	1.507344	-0.117918
26.	8.	0.	-7.113251	2.014047	0.068852
27.	8.	0.	-0.947232	-1.455877	0.362637
28.	6.	0.	-3.685887	1.136058	-2.369878
29.	6.	0.	-3.050484	-3.168449	-0.448496
30.	6.	0.	-2.588827	0.265046	0.551224

31.	6.	0.	-1.978012	-1.875064	-2.305677
32.	8.	0.	-5.765884	0.754163	-1.254318
33.	8.	0.	-2.407036	0.884075	2.905392
34.	1.	0.	5.270562	4.152072	0.135964
35.	1.	0.	6.432805	2.471984	1.540688
36.	1.	0.	6.043104	0.040735	1.175039
37.	1.	0.	3.323818	0.994509	-2.024226
38.	1.	0.	3.900603	-1.280789	-1.610975
39.	1.	0.	3.796096	4.438674	-1.609961
40.	1.	0.	3.441179	-2.805409	0.312080
41.	1.	0.	3.890919	-1.528793	1.437134
42.	1.	0.	5.952585	-1.976150	-2.603648
43.	1.	0.	7.289178	-2.393739	-1.498795
44.	1.	0.	6.811671	-0.689457	-1.714759
45.	1.	0.	1.536540	-1.445345	-0.539219
46.	1.	0.	2.065110	-0.069129	0.412947
47.	1.	0.	1.584628	-1.299451	2.512965
48.	1.	0.	0.577391	-3.563494	0.710763
49.	1.	0.	0.299365	-3.454261	2.454326
50.	1.	0.	1.941218	-3.639021	1.836611
51.	1.	0.	-0.841313	-1.345544	2.414653
52.	1.	0.	0.104132	0.946763	0.656085
53.	1.	0.	-1.842633	2.079487	1.392518
54.	1.	0.	0.301489	2.253313	2.802175
55.	1.	0.	0.080756	0.770624	3.725172
56.	1.	0.	1.519297	0.978416	2.699537
57.	1.	0.	-4.801751	1.931634	1.718409
58.	1.	0.	-4.569026	-1.502812	-2.366241
59.	1.	0.	-4.996131	-1.588750	-0.662570
60.	1.	0.	-4.059049	0.807450	-3.344069
61.	1.	0.	-2.602265	1.004111	-2.342246
62.	1.	0.	-3.904016	2.200916	-2.256801
63.	1.	0.	-2.091688	-3.655188	-0.258673
64.	1.	0.	-3.611458	-3.133004	0.491051
65.	1.	0.	-3.616994	-3.783534	-1.155439
66.	1.	0.	-2.460884	-2.549904	-3.019759
67.	1.	0.	-1.821730	-0.915677	-2.803614
68.	1.	0.	-0.998469	-2.288745	-2.054511
69.	1.	0.	-2.748708	-0.016434	2.984176

**Isomer 2.-3**		Standard Orientation (Ångstroms)			
Center	Atom	Type	X	Y	Z

number	number				
1.	6.	0.	5.808017	-1.832589	0.380167
2.	6.	0.	5.200611	-2.942152	-0.215490
3.	6.	0.	4.047209	-2.763026	-0.980606
4.	6.	0.	3.494085	-1.495404	-1.150020
5.	6.	0.	4.102698	-0.383174	-0.551415
6.	6.	0.	5.261023	-0.556928	0.206838
7.	6.	0.	3.478603	1.000407	-0.683769
8.	8.	0.	6.942804	-1.928170	1.137026
9.	6.	0.	2.364556	1.219527	0.351357
10.	8.	0.	2.885844	1.205008	-1.964773
11.	6.	0.	3.822654	1.368740	-3.015725
12.	6.	0.	1.825071	2.656892	0.331619
13.	6.	0.	0.620955	2.969455	1.250314
14.	6.	0.	0.927040	2.723059	2.736540
15.	6.	0.	-0.712467	2.315671	0.841383
16.	6.	0.	-1.150927	2.479389	-0.618326
17.	6.	0.	-2.532093	1.812915	-0.798235
18.	6.	0.	-1.186573	3.941317	-1.079430
19.	6.	0.	-3.622214	-0.484183	-0.472018
20.	6.	0.	-4.886771	-0.321470	-0.904959
21.	6.	0.	-1.645370	0.087155	0.788453
22.	6.	0.	-1.624466	-1.241107	1.550114
23.	6.	0.	-2.851404	-2.135561	1.185028
24.	6.	0.	-3.367544	-1.957243	-0.247853
25.	6.	0.	-5.570527	-1.627071	-0.864865
26.	8.	0.	-6.711168	-1.918385	-1.129745
27.	8.	0.	-0.644129	0.904418	1.190940
28.	6.	0.	-2.465803	-2.559758	-1.334716
29.	6.	0.	-1.701944	-0.933729	3.066612
30.	6.	0.	-2.566934	0.453071	-0.146481
31.	6.	0.	-0.286973	-1.974920	1.282770
32.	8.	0.	-4.664522	-2.569895	-0.395321
33.	8.	0.	-3.613728	2.644147	-0.344485
34.	1.	0.	5.629264	-3.933815	-0.087243
35.	1.	0.	3.580608	-3.625314	-1.448433
36.	1.	0.	2.604828	-1.352429	-1.754514
37.	1.	0.	5.759232	0.289101	0.671809
38.	1.	0.	4.267468	1.756587	-0.525538
39.	1.	0.	7.222603	-2.852343	1.171125
40.	1.	0.	1.565784	0.502885	0.141613
41.	1.	0.	2.762311	0.966433	1.340258
42.	1.	0.	4.480271	2.232883	-2.836638

43.	1.	0.	3.249133	1.544553	-3.928832
44.	1.	0.	4.448248	0.476922	-3.153199
45.	1.	0.	1.574938	2.911867	-0.702207
46.	1.	0.	2.637928	3.338060	0.620237
47.	1.	0.	0.433314	4.046230	1.141192
48.	1.	0.	0.097891	3.057032	3.369917
49.	1.	0.	1.821341	3.277328	3.039489
50.	1.	0.	1.097461	1.664735	2.946353
51.	1.	0.	-1.499457	2.748049	1.482681
52.	1.	0.	-0.444266	1.921032	-1.246989
53.	1.	0.	-2.716304	1.699201	-1.872916
54.	1.	0.	-1.592001	4.004048	-2.094009
55.	1.	0.	-1.835837	4.540649	-0.435903
56.	1.	0.	-0.190099	4.389487	-1.092227
57.	1.	0.	-5.395045	0.599933	-1.151347
58.	1.	0.	-2.604859	-3.185426	1.377581
59.	1.	0.	-3.687528	-1.880799	1.844920
60.	1.	0.	-1.503774	-2.046131	-1.388691
61.	1.	0.	-2.958587	-2.467816	-2.305835
62.	1.	0.	-2.291995	-3.620733	-1.133517
63.	1.	0.	-0.828891	-0.370374	3.402357
64.	1.	0.	-2.599963	-0.354937	3.306021
65.	1.	0.	-1.746057	-1.871779	3.629739
66.	1.	0.	-0.244344	-2.889079	1.883601
67.	1.	0.	-0.165277	-2.257059	0.234849
68.	1.	0.	0.561226	-1.346148	1.562108
69.	1.	0.	-3.733426	2.474512	0.599330

**Isomer 2.-4**		Standard Orientation (Ångstroms)			
Center number	Atom number	Type	X	Y	Z
1.	6.	0.	-5.635977	-2.201035	-0.250034
2.	6.	0.	-4.936504	-2.858727	-1.269812
3.	6.	0.	-4.037591	-2.139208	-2.050060
4.	6.	0.	-3.827288	-0.774999	-1.825919
5.	6.	0.	-4.514524	-0.119959	-0.800019
6.	6.	0.	-5.424091	-0.840704	-0.016240
7.	6.	0.	-4.271460	1.354198	-0.500807
8.	8.	0.	-6.516606	-2.943887	0.485072
9.	6.	0.	-3.361947	1.578664	0.719561
10.	8.	0.	-5.482772	2.046454	-0.205891
11.	6.	0.	-6.333721	2.221351	-1.325592

12.	6.	0.	-1.927117	1.071792	0.529755
13.	6.	0.	-1.055766	1.179417	1.798182
14.	6.	0.	-0.888964	2.629050	2.283842
15.	6.	0.	0.319495	0.509561	1.629333
16.	6.	0.	0.347661	-0.978632	1.262229
17.	6.	0.	1.821269	-1.437938	1.209145
18.	6.	0.	-0.474715	-1.855357	2.212344
19.	6.	0.	3.968448	-0.841338	-0.061734
20.	6.	0.	4.880022	-1.731004	0.374406
21.	6.	0.	2.229379	0.812541	0.184384
22.	6.	0.	3.014362	1.896188	-0.558468
23.	6.	0.	4.438053	1.402497	-0.967229
24.	6.	0.	4.537114	-0.101164	-1.251065
25.	6.	0.	6.125819	-1.543803	-0.391805
26.	8.	0.	7.191539	-2.101143	-0.295767
27.	8.	0.	1.042111	1.282463	0.631974
28.	6.	0.	3.894284	-0.548061	-2.572353
29.	6.	0.	3.191828	3.108814	0.389344
30.	6.	0.	2.658498	-0.458851	0.423275
31.	6.	0.	2.198828	2.369374	-1.787395
32.	8.	0.	5.918044	-0.512874	-1.300084
33.	8.	0.	2.372864	-1.686720	2.512400
34.	1.	0.	-5.116635	-3.915491	-1.435221
35.	1.	0.	-3.501052	-2.644225	-2.848195
36.	1.	0.	-3.132705	-0.220877	-2.451277
37.	1.	0.	-5.979070	-0.322835	0.763231
38.	1.	0.	-3.795433	1.813020	-1.384781
39.	1.	0.	-6.932649	-2.375200	1.146347
40.	1.	0.	-3.372905	2.653965	0.923961
41.	1.	0.	-3.823911	1.086867	1.585493
42.	1.	0.	-5.834359	2.790467	-2.124657
43.	1.	0.	-7.201342	2.787570	-0.978966
44.	1.	0.	-6.674344	1.263877	-1.741784
45.	1.	0.	-1.442248	1.626942	-0.283610
46.	1.	0.	-1.966922	0.025906	0.207519
47.	1.	0.	-1.555813	0.619349	2.599893
48.	1.	0.	-0.238342	2.675053	3.163990
49.	1.	0.	-1.849940	3.067401	2.564555
50.	1.	0.	-0.443917	3.256281	1.505815
51.	1.	0.	0.870922	0.632014	2.576575
52.	1.	0.	-0.046760	-1.084544	0.242955
53.	1.	0.	1.854934	-2.415092	0.713399
54.	1.	0.	-0.338295	-2.911637	1.960809

55.	1.	0.	-0.149601	-1.725714	3.248018
56.	1.	0.	-1.543111	-1.634755	2.145702
57.	1.	0.	4.800967	-2.414549	1.207890
58.	1.	0.	4.788882	1.980526	-1.829042
59.	1.	0.	5.134124	1.604670	-0.146359
60.	1.	0.	2.811449	-0.406630	-2.562411
61.	1.	0.	4.099510	-1.609170	-2.734304
62.	1.	0.	4.318587	0.017804	-3.406623
63.	1.	0.	2.229125	3.550758	0.654390
64.	1.	0.	3.702985	2.816089	1.312246
65.	1.	0.	3.800061	3.874776	-0.102968
66.	1.	0.	2.717941	3.199306	-2.277500
67.	1.	0.	2.059597	1.577416	-2.526357
68.	1.	0.	1.211452	2.720426	-1.478110
69.	1.	0.	2.725013	-0.850120	2.844157

**Isomer 2.-5**		Standard Orientation (Ångstroms)			
Center number	Atom number	Type	X	Y	Z
1.	6.	0.	4.002342	-2.809410	-0.428477
2.	6.	0.	5.132080	-2.965817	0.382313
3.	6.	0.	5.781698	-1.835286	0.877222
4.	6.	0.	5.315670	-0.557498	0.570195
5.	6.	0.	4.180068	-0.401334	-0.234612
6.	6.	0.	3.528216	-1.530816	-0.734282
7.	6.	0.	3.617569	0.984931	-0.523622
8.	8.	0.	3.317896	-3.871688	-0.951215
9.	6.	0.	2.472767	1.334459	0.439051
10.	8.	0.	3.083241	1.089901	-1.841586
11.	6.	0.	4.064559	1.106100	-2.864070
12.	6.	0.	1.965847	2.772247	0.253899
13.	6.	0.	0.712539	3.183442	1.060872
14.	6.	0.	0.929973	3.099008	2.580429
15.	6.	0.	-0.598556	2.487630	0.646853
16.	6.	0.	-0.937584	2.472550	-0.848701
17.	6.	0.	-2.305927	1.782508	-1.040634
18.	6.	0.	-0.938113	3.869185	-1.481393
19.	6.	0.	-3.423751	-0.465158	-0.515699
20.	6.	0.	-4.652752	-0.364333	-1.056450
21.	6.	0.	-1.541594	0.267495	0.803553
22.	6.	0.	-1.583536	-0.953518	1.726287
23.	6.	0.	-2.782080	-1.894868	1.385808

24.	6.	0.	-3.191550	-1.897390	-0.091846
25.	6.	0.	-5.342839	-1.658030	-0.902976
26.	8.	0.	-6.462823	-1.985485	-1.210889
27.	8.	0.	-0.566791	1.131063	1.171809
28.	6.	0.	-2.215016	-2.626073	-1.026303
29.	6.	0.	-1.775423	-0.455782	3.181004
30.	6.	0.	-2.391210	0.510470	-0.233736
31.	6.	0.	-0.231932	-1.706826	1.655952
32.	8.	0.	-4.477387	-2.530111	-0.254928
33.	8.	0.	-3.412629	2.657579	-0.763561
34.	1.	0.	5.501603	-3.961497	0.618257
35.	1.	0.	6.663051	-1.956877	1.500321
36.	1.	0.	5.833236	0.317832	0.953352
37.	1.	0.	2.659367	-1.424285	-1.374763
38.	1.	0.	4.428967	1.723945	-0.402420
39.	1.	0.	3.747504	-4.691637	-0.674348
40.	1.	0.	1.666010	0.616231	0.270825
41.	1.	0.	2.826356	1.173672	1.463575
42.	1.	0.	4.760965	1.949259	-2.739050
43.	1.	0.	3.534422	1.223338	-3.812031
44.	1.	0.	4.645518	0.174911	-2.892583
45.	1.	0.	1.783385	2.932375	-0.812437
46.	1.	0.	2.772258	3.466969	0.528113
47.	1.	0.	0.538915	4.242341	0.825951
48.	1.	0.	0.066444	3.496870	3.124763
49.	1.	0.	1.807037	3.685314	2.873330
50.	1.	0.	1.084042	2.069657	2.912167
51.	1.	0.	-1.421860	2.996350	1.176815
52.	1.	0.	-0.191692	1.847111	-1.357314
53.	1.	0.	-2.417616	1.540507	-2.103885
54.	1.	0.	-1.262262	3.807144	-2.524898
55.	1.	0.	-1.637213	4.533149	-0.966420
56.	1.	0.	0.055718	4.323000	-1.469047
57.	1.	0.	-5.138361	0.516429	-1.452029
58.	1.	0.	-2.551423	-2.911511	1.722106
59.	1.	0.	-3.663563	-1.567563	1.947247
60.	1.	0.	-1.249974	-2.117563	-1.077146
61.	1.	0.	-2.636488	-2.661947	-2.033989
62.	1.	0.	-2.057430	-3.651490	-0.680064
63.	1.	0.	-0.931687	0.156625	3.505704
64.	1.	0.	-2.690365	0.137975	3.277232
65.	1.	0.	-1.860053	-1.314256	3.855489
66.	1.	0.	-0.236714	-2.535871	2.370959

67.	1.	0.	-0.032085	-2.120743	0.665314
68.	1.	0.	0.594281	-1.041041	1.915052
69.	1.	0.	-3.591136	2.607896	0.184990

Table S1.3.1.3.a. Gibbs free energies[a] and equilibrium populations[b] of low-energy conformers of **isomer 1'**.

Conformers	In MeOH	
	ΔG	P (%)/100
**isomer 1'.-1**	0.00	0.165
**isomer 1'.-2**	0.10	0.140
**isomer 1'.-3**	0.23	0.112
**isomer 1'.-4**	0.29	0.101
**isomer 1'.-5**	0.47	0.075

[a]B3LYP/6-31+G (d,p), in kcal/mol. [b]From ΔG values at 298.15K.

Table S1.3.1.3.b. Cartesian coordinates for the low-energy reoptimized MMFF conformers of **isomer 1'** at B3LYP/6-311+G (d,p) level of theory in CH_3OH.

**Isomer 1'.-1**		Standard Orientation (Ångstroms)			
Center number	Atomic number	Atomic Type	X	Y	Z
1.	6.	0.	1.689964	2.658361	-0.381373
2.	6.	0.	1.977730	3.078289	0.923001
3.	6.	0.	2.837791	2.314185	1.713972
4.	6.	0.	3.402939	1.135717	1.228865
5.	6.	0.	3.112106	0.710260	-0.075998
6.	6.	0.	2.271523	1.483828	-0.878133
7.	6.	0.	3.672893	-0.609215	-0.592537
8.	8.	0.	0.855927	3.344518	-1.211871
9.	6.	0.	2.975652	-1.837833	0.018296
10.	8.	0.	5.054933	-0.770323	-0.281768
11.	6.	0.	5.909030	0.121938	-0.975513
12.	6.	0.	1.473706	-1.923049	-0.284186
13.	6.	0.	0.815636	-3.211889	0.264968
14.	6.	0.	1.108097	-4.405378	-0.661887
15.	6.	0.	-0.714485	-3.119141	0.528771
16.	6.	0.	-1.158993	-2.545054	1.884838
17.	6.	0.	-1.000252	-1.009202	1.905088
18.	6.	0.	-2.593634	-2.980093	2.224791
19.	6.	0.	-1.890133	1.003123	0.582226
20.	6.	0.	-1.370428	2.054347	1.254179
21.	6.	0.	-1.734404	-1.149494	-0.492325
22.	6.	0.	-2.242905	-0.631567	-1.841232
23.	6.	0.	-2.374203	0.920089	-1.840834

24.	6.	0.	-2.804257	1.527683	-0.502079
25.	6.	0.	-1.674753	3.267248	0.495125
26.	8.	0.	-1.206240	4.387581	0.602338
27.	8.	0.	-1.384124	-2.451864	-0.581315
28.	6.	0.	-4.285650	1.352973	-0.144296
29.	6.	0.	-3.584292	-1.333467	-2.173766
30.	6.	0.	-1.612415	-0.412176	0.652170
31.	6.	0.	-1.232622	-1.005951	-2.952918
32.	8.	0.	-2.544182	2.952220	-0.522420
33.	8.	0.	-1.520882	-0.431433	3.100221
34.	1.	0.	1.516816	3.982546	1.309316
35.	1.	0.	3.064390	2.646515	2.723372
36.	1.	0.	4.078357	0.549845	1.843948
37.	1.	0.	2.043303	1.184851	-1.897639
38.	1.	0.	3.540835	-0.637286	-1.688708
39.	1.	0.	0.357050	4.008968	-0.703409
40.	1.	0.	3.503030	-2.722345	-0.356302
41.	1.	0.	3.135217	-1.812617	1.103491
42.	1.	0.	5.708688	1.170659	-0.719667
43.	1.	0.	6.931809	-0.126974	-0.682742
44.	1.	0.	5.813426	0.005082	-2.066295
45.	1.	0.	1.313682	-1.885475	-1.368974
46.	1.	0.	0.990742	-1.030519	0.116987
47.	1.	0.	1.263580	-3.429622	1.245232
48.	1.	0.	0.584257	-4.286349	-1.616737
49.	1.	0.	0.782360	-5.350514	-0.214355
50.	1.	0.	2.176934	-4.492892	-0.875654
51.	1.	0.	-1.107747	-4.139457	0.466972
52.	1.	0.	-0.492605	-2.960708	2.649462
53.	1.	0.	0.067348	-0.761087	1.947078
54.	1.	0.	-2.668566	-4.071623	2.249307
55.	1.	0.	-2.883782	-2.607774	3.210561
56.	1.	0.	-3.316623	-2.614231	1.488462
57.	1.	0.	-0.685404	2.043464	2.087915
58.	1.	0.	-1.397195	1.359419	-2.068233
59.	1.	0.	-3.058095	1.227181	-2.639735
60.	1.	0.	-4.917882	1.719351	-0.957979
61.	1.	0.	-4.529510	0.306248	0.048318
62.	1.	0.	-4.510803	1.929304	0.756762
63.	1.	0.	-3.450631	-2.418260	-2.177565
64.	1.	0.	-4.373776	-1.093747	-1.458864
65.	1.	0.	-3.926400	-1.027788	-3.167817
66.	1.	0.	-0.241602	-0.594579	-2.740765

67.	1.	0.	-1.143636	-2.088403	-3.062745
68.	1.	0.	-1.572257	-0.588646	-3.906582
69.	1.	0.	-2.480625	-0.365329	3.006823

**Isomer 1'.-2**		Standard Orientation (Ångstroms)			
Center number	Atom number	Type	X	Y	Z
1.	6.	0.	1.184359	-2.587241	-0.270211
2.	6.	0.	1.339542	-2.587203	-1.660407
3.	6.	0.	2.273144	-1.725655	-2.242668
4.	6.	0.	3.040586	-0.860547	-1.464069
5.	6.	0.	2.870907	-0.838692	-0.070249
6.	6.	0.	1.954229	-1.714939	0.513459
7.	6.	0.	3.714113	0.072876	0.813772
8.	8.	0.	0.316111	-3.404838	0.386022
9.	6.	0.	3.660615	1.579231	0.500271
10.	8.	0.	5.104433	-0.251417	0.712317
11.	6.	0.	5.450182	-1.522708	1.230621
12.	6.	0.	2.306883	2.302376	0.660822
13.	6.	0.	1.336142	2.251342	-0.542966
14.	6.	0.	1.945555	2.927688	-1.783849
15.	6.	0.	-0.013666	2.929382	-0.186833
16.	6.	0.	-1.053279	2.976507	-1.316066
17.	6.	0.	-1.414787	1.522286	-1.687897
18.	6.	0.	-2.277867	3.819534	-0.930747
19.	6.	0.	-2.261743	-0.605600	-0.536637
20.	6.	0.	-2.119174	-1.535509	-1.506381
21.	6.	0.	-1.337494	1.167437	0.802750
22.	6.	0.	-1.651543	0.479375	2.132271
23.	6.	0.	-2.098020	-0.996501	1.911156
24.	6.	0.	-2.939048	-1.246618	0.653406
25.	6.	0.	-2.380414	-2.843196	-0.907763
26.	8.	0.	-2.087261	-3.952238	-1.320094
27.	8.	0.	-0.609693	2.292596	0.984573
28.	6.	0.	-4.415511	-0.843072	0.759050
29.	6.	0.	-2.727611	1.318609	2.869616
30.	6.	0.	-1.731890	0.733433	-0.431224
31.	6.	0.	-0.392480	0.448597	3.028915
32.	8.	0.	-2.915231	-2.663472	0.346063
33.	8.	0.	-2.464861	1.444551	-2.649742
34.	1.	0.	0.738257	-3.256005	-2.269949
35.	1.	0.	2.402924	-1.738845	-3.321379

36.	1.	0.	3.777001	-0.214055	-1.930560
37.	1.	0.	1.829320	-1.748044	1.592010
38.	1.	0.	3.386314	-0.076868	1.857465
39.	1.	0.	-0.269628	-3.866421	-0.240564
40.	1.	0.	4.379924	2.037268	1.187672
41.	1.	0.	4.078714	1.734214	-0.499494
42.	1.	0.	6.535376	-1.613840	1.141767
43.	1.	0.	5.172139	-1.616027	2.292287
44.	1.	0.	4.976687	-2.343222	0.675750
45.	1.	0.	2.517697	3.360476	0.871247
46.	1.	0.	1.795396	1.919324	1.549581
47.	1.	0.	1.141746	1.200563	-0.781465
48.	1.	0.	2.051206	4.009445	-1.633378
49.	1.	0.	1.343408	2.769083	-2.681882
50.	1.	0.	2.938979	2.528762	-1.997472
51.	1.	0.	0.192025	3.947411	0.164332
52.	1.	0.	-0.596799	3.445692	-2.191868
53.	1.	0.	-0.561013	1.065536	-2.203290
54.	1.	0.	-2.980459	3.871977	-1.767054
55.	1.	0.	-2.802955	3.415176	-0.060496
56.	1.	0.	-1.976833	4.843959	-0.689799
57.	1.	0.	-1.681566	-1.419989	-2.486671
58.	1.	0.	-1.206417	-1.622107	1.803181
59.	1.	0.	-2.630057	-1.350154	2.801436
60.	1.	0.	-4.873971	-1.319139	1.630413
61.	1.	0.	-4.531295	0.238929	0.849098
62.	1.	0.	-4.946290	-1.172185	-0.137842
63.	1.	0.	-3.665503	1.384426	2.314568
64.	1.	0.	-2.942672	0.872420	3.846100
65.	1.	0.	-2.362709	2.336296	3.033810
66.	1.	0.	-0.077045	1.455536	3.308829
67.	1.	0.	-0.611697	-0.110335	3.944594
68.	1.	0.	0.438555	-0.047280	2.520893
69.	1.	0.	-3.297111	1.629416	-2.193841

**Isomer 1'.-3**		Standard Orientation (Ångstroms)			
Center number	Atom number	Type	X	Y	Z
1.	6.	0.	5.672581	2.187649	-0.161524
2.	6.	0.	4.999648	2.866415	-1.185318
3.	6.	0.	4.119351	2.164045	-2.001639
4.	6.	0.	3.901677	0.796140	-1.809559

5.	6.	0.	4.562527	0.119886	-0.780068
6.	6.	0.	5.453265	0.823375	0.040106
7.	6.	0.	4.310471	-1.359506	-0.516415
8.	8.	0.	6.535675	2.914395	0.609831
9.	6.	0.	3.368736	-1.607551	0.674321
10.	8.	0.	5.513482	-2.058624	-0.203266
11.	6.	0.	6.391809	-2.215613	-1.304129
12.	6.	0.	1.939377	-1.095753	0.458281
13.	6.	0.	1.030959	-1.244542	1.696270
14.	6.	0.	0.832356	-2.711144	2.114793
15.	6.	0.	-0.329991	-0.551391	1.520987
16.	6.	0.	-0.331571	0.953734	1.216528
17.	6.	0.	-1.795480	1.419110	1.201170
18.	6.	0.	0.515779	1.780407	2.190983
19.	6.	0.	-3.963120	0.885767	-0.060411
20.	6.	0.	-4.534816	2.092749	-0.260837
21.	6.	0.	-2.206713	-0.763279	0.031474
22.	6.	0.	-2.899322	-1.713468	-0.947165
23.	6.	0.	-4.240297	-1.113742	-1.472922
24.	6.	0.	-4.947956	-0.185686	-0.478332
25.	6.	0.	-5.824032	1.900534	-0.946933
26.	8.	0.	-6.621635	2.713494	-1.346146
27.	8.	0.	-1.022516	-1.254373	0.447729
28.	6.	0.	-5.577636	-0.894859	0.729274
29.	6.	0.	-3.121827	-3.090831	-0.275197
30.	6.	0.	-2.657918	0.472303	0.395822
31.	6.	0.	-1.957778	-1.922248	-2.160196
32.	8.	0.	-6.008610	0.537587	-1.134639
33.	8.	0.	-2.234962	1.519208	2.562483
34.	1.	0.	5.185338	3.925886	-1.325457
35.	1.	0.	3.603490	2.685617	-2.802749
36.	1.	0.	3.221829	0.255626	-2.462353
37.	1.	0.	5.987710	0.288921	0.822654
38.	1.	0.	3.857811	-1.800338	-1.421514
39.	1.	0.	6.932697	2.332357	1.271193
40.	1.	0.	3.373604	-2.686949	0.856269
41.	1.	0.	3.807849	-1.134834	1.562495
42.	1.	0.	6.742870	-1.251712	-1.696201
43.	1.	0.	7.250576	-2.787911	-0.945525
44.	1.	0.	5.912402	-2.771355	-2.124586
45.	1.	0.	1.479993	-1.625145	-0.386383
46.	1.	0.	1.986594	-0.040390	0.169522
47.	1.	0.	1.515131	-0.725793	2.534834

48.	1.	0.	1.780118	-3.177706	2.395368
49.	1.	0.	0.394191	-3.296525	1.300795
50.	1.	0.	0.162304	-2.783911	2.978171
51.	1.	0.	-0.925147	-0.705151	2.431942
52.	1.	0.	0.052797	1.095693	0.198269
53.	1.	0.	-1.837322	2.414784	0.731011
54.	1.	0.	0.382901	2.848295	1.991873
55.	1.	0.	0.215917	1.601166	3.226304
56.	1.	0.	1.580271	1.554915	2.086612
57.	1.	0.	-4.130456	3.078777	-0.076507
58.	1.	0.	-4.042514	-0.513100	-2.367000
59.	1.	0.	-4.907540	-1.926759	-1.778532
60.	1.	0.	-6.268834	-1.672065	0.391301
61.	1.	0.	-4.819184	-1.347396	1.371154
62.	1.	0.	-6.140098	-0.168361	1.321500
63.	1.	0.	-2.176112	-3.494628	0.092965
64.	1.	0.	-3.533275	-3.793562	-1.006836
65.	1.	0.	-3.816855	-3.038195	0.565558
66.	1.	0.	-2.450016	-2.558849	-2.902886
67.	1.	0.	-1.714824	-0.968388	-2.638799
68.	1.	0.	-1.025403	-2.404277	-1.857514
69.	1.	0.	-3.190759	1.662754	2.548943

**Isomer 1'.-4**		Standard Orientation (Ångstroms)			
Center number	Atom number	Type	X	Y	Z
1.	6.	0.	4.295235	2.616718	-1.077473
2.	6.	0.	5.141174	3.061253	-0.058072
3.	6.	0.	5.762120	2.126634	0.773360
4.	6.	0.	5.540687	0.762563	0.602080
5.	6.	0.	4.688021	0.315135	-0.418587
6.	6.	0.	4.075861	1.245975	-1.258641
7.	6.	0.	4.416235	-1.175295	-0.584484
8.	8.	0.	3.656866	3.468940	-1.935284
9.	6.	0.	3.450811	-1.730443	0.476309
10.	8.	0.	5.605616	-1.951254	-0.461776
11.	6.	0.	6.514590	-1.789079	-1.536142
12.	6.	0.	2.026634	-1.170954	0.373840
13.	6.	0.	1.094552	-1.614605	1.520549
14.	6.	0.	0.918784	-3.141153	1.589577
15.	6.	0.	-0.276194	-0.920021	1.469546
16.	6.	0.	-0.296740	0.614789	1.502623

17.	6.	0.	-1.766382	1.060432	1.537523
18.	6.	0.	0.505156	1.214549	2.664032
19.	6.	0.	-3.875411	0.810375	0.101808
20.	6.	0.	-4.450443	2.031738	0.145725
21.	6.	0.	-2.105180	-0.815781	-0.093200
22.	6.	0.	-2.749313	-1.534608	-1.279885
23.	6.	0.	-4.070489	-0.833441	-1.723758
24.	6.	0.	-4.830211	-0.143189	-0.585091
25.	6.	0.	-5.705236	1.995060	-0.624232
26.	8.	0.	-6.492792	2.875876	-0.870589
27.	8.	0.	-0.931720	-1.380479	0.251170
28.	6.	0.	-5.508865	-1.097392	0.408657
29.	6.	0.	-2.989013	-3.022892	-0.925495
30.	6.	0.	-2.586194	0.309256	0.512151
31.	6.	0.	-1.757257	-1.482578	-2.469607
32.	8.	0.	-5.867080	0.707193	-1.115914
33.	8.	0.	-2.253550	0.853712	2.870657
34.	1.	0.	5.317526	4.125820	0.080400
35.	1.	0.	6.425604	2.473804	1.560310
36.	1.	0.	6.035616	0.036275	1.238231
37.	1.	0.	3.424886	0.927298	-2.067559
38.	1.	0.	3.975425	-1.339786	-1.583407
39.	1.	0.	3.900787	4.378192	-1.717558
40.	1.	0.	3.451878	-2.818809	0.359355
41.	1.	0.	3.870425	-1.517481	1.467911
42.	1.	0.	6.052626	-2.058758	-2.498445
43.	1.	0.	6.893559	-0.760884	-1.608616
44.	1.	0.	7.352689	-2.463715	-1.346313
45.	1.	0.	1.580626	-1.468999	-0.583662
46.	1.	0.	2.081940	-0.077074	0.359083
47.	1.	0.	1.547788	-1.292564	2.467833
48.	1.	0.	0.223321	-3.418373	2.389160
49.	1.	0.	1.868696	-3.642278	1.792076
50.	1.	0.	0.521427	-3.534659	0.649171
51.	1.	0.	-0.887030	-1.278759	2.309733
52.	1.	0.	0.118537	0.980651	0.554935
53.	1.	0.	-1.807307	2.135147	1.297860
54.	1.	0.	0.365717	2.299696	2.695359
55.	1.	0.	0.170647	0.814439	3.624258
56.	1.	0.	1.575481	1.021453	2.553861
57.	1.	0.	-4.065083	2.953200	0.560621
58.	1.	0.	-3.835833	-0.053133	-2.455251
59.	1.	0.	-4.715776	-1.559433	-2.229655

60.	1.	0.	-4.778118	-1.680994	0.972336
61.	1.	0.	-6.104822	-0.516410	1.117287
62.	1.	0.	-6.176805	-1.781075	-0.122930
63.	1.	0.	-2.055324	-3.497764	-0.616146
64.	1.	0.	-3.368175	-3.553425	-1.804753
65.	1.	0.	-3.714858	-3.149841	-0.119405
66.	1.	0.	-1.502720	-0.449279	-2.725453
67.	1.	0.	-0.834204	-2.018406	-2.237835
68.	1.	0.	-2.214135	-1.946896	-3.349897
69.	1.	0.	-3.211328	0.982646	2.852309

**Isomer 1'.-5**		Standard Orientation (Ångstroms)			
Center number	Atom number	Type	X	Y	Z
1.	6.	0.	1.710622	2.654422	-0.383567
2.	6.	0.	2.004909	3.072369	0.920421
3.	6.	0.	2.861329	2.302140	1.709365
4.	6.	0.	3.416515	1.118977	1.223591
5.	6.	0.	3.119325	0.695476	-0.080643
6.	6.	0.	2.282601	1.475035	-0.880686
7.	6.	0.	3.670731	-0.627235	-0.599191
8.	8.	0.	0.882622	3.347068	-1.212515
9.	6.	0.	2.965472	-1.852247	0.009423
10.	8.	0.	5.051791	-0.797844	-0.288368
11.	6.	0.	5.911840	0.088150	-0.983067
12.	6.	0.	1.463484	-1.930481	-0.294939
13.	6.	0.	0.799245	-3.215521	0.256390
14.	6.	0.	1.082727	-4.410332	-0.671597
15.	6.	0.	-0.729613	-3.114778	0.524322
16.	6.	0.	-1.173288	-2.538340	1.880323
17.	6.	0.	-1.004385	-1.004034	1.899228
18.	6.	0.	-2.614059	-2.961021	2.209707
19.	6.	0.	-1.888062	1.013584	0.588164
20.	6.	0.	-1.345397	2.063580	1.241877
21.	6.	0.	-1.748373	-1.139210	-0.485790
22.	6.	0.	-2.266865	-0.621531	-1.830296
23.	6.	0.	-2.395975	0.929898	-1.829946
24.	6.	0.	-2.809870	1.540821	-0.487263
25.	6.	0.	-1.649962	3.274554	0.483072
26.	8.	0.	-1.167022	4.391393	0.577324
27.	8.	0.	-1.399957	-2.444546	-0.580081
28.	6.	0.	-4.287322	1.375620	-0.111530

29.	6.	0.	-3.613909	-1.319656	-2.147425
30.	6.	0.	-1.620156	-0.403472	0.656859
31.	6.	0.	-1.268800	-0.999283	-2.951625
32.	8.	0.	-2.539246	2.964470	-0.516845
33.	8.	0.	-1.627430	-0.408972	3.039538
34.	1.	0.	1.551966	3.980622	1.306610
35.	1.	0.	3.095280	2.635019	2.717123
36.	1.	0.	4.091529	0.529516	1.835850
37.	1.	0.	2.048943	1.177190	-1.899238
38.	1.	0.	3.538531	-0.652758	-1.695287
39.	1.	0.	0.376338	4.004918	-0.701231
40.	1.	0.	3.488729	-2.739494	-0.364494
41.	1.	0.	3.124089	-1.828526	1.094967
42.	1.	0.	5.717793	1.138451	-0.728894
43.	1.	0.	6.932927	-0.166633	-0.689435
44.	1.	0.	5.815977	-0.029810	-2.073650
45.	1.	0.	1.304604	-1.894435	-1.379900
46.	1.	0.	0.983485	-1.034870	0.103117
47.	1.	0.	1.249640	-3.436438	1.235190
48.	1.	0.	0.753861	-5.353966	-0.223224
49.	1.	0.	2.150329	-4.502997	-0.889635
50.	1.	0.	0.555605	-4.288139	-1.624152
51.	1.	0.	-1.127914	-4.133515	0.467245
52.	1.	0.	-0.508059	-2.966459	2.642429
53.	1.	0.	0.069516	-0.759864	1.922397
54.	1.	0.	-2.944474	-2.504084	3.143837
55.	1.	0.	-3.310016	-2.652009	1.424635
56.	1.	0.	-2.678948	-4.049359	2.308678
57.	1.	0.	-0.649905	2.054040	2.066338
58.	1.	0.	-1.420772	1.366994	-2.069445
59.	1.	0.	-3.088379	1.237153	-2.621462
60.	1.	0.	-4.532808	0.331398	0.090694
61.	1.	0.	-4.496916	1.953829	0.791596
62.	1.	0.	-4.926620	1.742732	-0.919516
63.	1.	0.	-3.965316	-1.014738	-3.138527
64.	1.	0.	-4.393847	-1.074649	-1.424069
65.	1.	0.	-3.484506	-2.404998	-2.150190
66.	1.	0.	-0.274495	-0.590466	-2.749396
67.	1.	0.	-1.183734	-2.082068	-3.061691
68.	1.	0.	-1.616322	-0.581705	-3.902356
69.	1.	0.	-1.088745	-0.627341	3.811602

Table S1.3.1.4.a. Gibbs free energies[a] and equilibrium populations[b] of low-energy conformers of

isomer 2'.

Conformers	In MeOH	
	ΔG	P (%)/100
**isomer 2'.-1**	0.00	0.228
**isomer 2'.-2**	0.08	0.199
**isomer 2'.-3**	0.21	0.160
**isomer 2'.-4**	0.56	0.088
**isomer 2'.-5**	0.63	0.079

[a]B3LYP/6-31+G (d,p), in kcal/mol. [b]From ΔG values at 298.15K.

Table S1.3.1.4.b. Cartesian coordinates for the low-energy reoptimized MMFF conformers of **isomer 2'** at B3LYP/6-311+G (d,p) level of theory in CH_3OH.

**Isomer 2'.-1**		Standard Orientation (Ångstroms)			
Center number	Atomic number	Atomic Type	X	Y	Z
1.	6.0	0.000000	1.910340	-2.818699	0.739749
2.	6.0	0.000000	2.410138	-2.947574	2.041422
3.	6.0	0.000000	3.458540	-2.127012	2.454879
4.	6.0	0.000000	4.004366	-1.179593	1.586997
5.	6.0	0.000000	3.496856	-1.042470	0.289252
6.	6.0	0.000000	2.455544	-1.874813	-0.132163
7.	6.0	0.000000	4.023402	0.024654	-0.663983
8.	8.0	0.000000	0.882255	-3.587187	0.263789
9.	6.0	0.000000	3.013088	1.165837	-0.864466
10.	8.0	0.000000	4.274382	-0.497800	-1.966213
11.	6.0	0.000000	5.359749	-1.405943	-2.026090
12.	6.0	0.000000	2.806272	2.029385	0.388994
13.	6.0	0.000000	1.609444	3.010562	0.355575
14.	6.0	0.000000	1.623911	3.922115	-0.881591
15.	6.0	0.000000	0.222752	2.371429	0.562780
16.	6.0	0.000000	0.045397	1.472412	1.792473
17.	6.0	0.000000	-1.428942	1.020423	1.860058
18.	6.0	0.000000	0.474938	2.138882	3.104319
19.	6.0	0.000000	-3.129073	-0.225066	0.403961
20.	6.0	0.000000	-4.235774	-0.292950	1.168317
21.	6.0	0.000000	-1.249300	0.877928	-0.635727
22.	6.0	0.000000	-1.683040	0.498342	-2.054325
23.	6.0	0.000000	-3.053498	-0.249495	-2.055948
24.	6.0	0.000000	-3.318365	-1.103740	-0.810688
25.	6.0	0.000000	-5.252090	-1.088909	0.456067
26.	8.0	0.000000	-6.386654	-1.360463	0.765887
27.	8.0	0.000000	-0.128819	1.639131	-0.644431

28.	6.0	0.000000	-2.496048	-2.398188	-0.726749
29.	6.0	0.000000	-0.576439	-0.348828	-2.730185
30.	6.0	0.000000	-1.910273	0.550064	0.510446
31.	6.0	0.000000	-1.856387	1.799049	-2.877453
32.	8.0	0.000000	-4.708003	-1.490457	-0.756483
33.	8.0	0.000000	-2.293761	2.028253	2.410795
34.	1.0	0.000000	1.986216	-3.685216	2.719353
35.	1.0	0.000000	3.852822	-2.232310	3.461406
36.	1.0	0.000000	4.823934	-0.547444	1.917066
37.	1.0	0.000000	2.068502	-1.801105	-1.142834
38.	1.0	0.000000	4.960697	0.434606	-0.247905
39.	1.0	0.000000	0.587740	-4.192703	0.956834
40.	1.0	0.000000	3.379458	1.777566	-1.695561
41.	1.0	0.000000	2.065191	0.727339	-1.184781
42.	1.0	0.000000	5.474195	-1.693816	-3.073687
43.	1.0	0.000000	5.180403	-2.308043	-1.425898
44.	1.0	0.000000	6.295486	-0.938717	-1.681852
45.	1.0	0.000000	3.714501	2.623591	0.558893
46.	1.0	0.000000	2.711211	1.379736	1.265719
47.	1.0	0.000000	1.728327	3.662832	1.230814
48.	1.0	0.000000	2.584537	4.440787	-0.964964
49.	1.0	0.000000	1.464096	3.356934	-1.802614
50.	1.0	0.000000	0.838742	4.683305	-0.817098
51.	1.0	0.000000	-0.505946	3.196202	0.640270
52.	1.0	0.000000	0.643470	0.565032	1.634894
53.	1.0	0.000000	-1.497237	0.190566	2.573260
54.	1.0	0.000000	-0.064907	3.075801	3.265183
55.	1.0	0.000000	0.242350	1.483888	3.949777
56.	1.0	0.000000	1.548608	2.341536	3.127289
57.	1.0	0.000000	-4.437135	0.218135	2.099144
58.	1.0	0.000000	-3.861772	0.488396	-2.097316
59.	1.0	0.000000	-3.133071	-0.857808	-2.963335
60.	1.0	0.000000	-2.848854	-2.989507	0.122425
61.	1.0	0.000000	-2.634231	-2.988762	-1.637020
62.	1.0	0.000000	-1.430360	-2.207320	-0.587597
63.	1.0	0.000000	-0.403445	-1.296820	-2.216499
64.	1.0	0.000000	-0.865852	-0.572870	-3.762019
65.	1.0	0.000000	0.367552	0.200575	-2.758135
66.	1.0	0.000000	-2.221817	1.553999	-3.880122
67.	1.0	0.000000	-0.910355	2.335204	-2.977129
68.	1.0	0.000000	-2.584827	2.468703	-2.408569
69.	1.0	0.000000	-2.586510	2.589076	1.680317

**Isomer 2'.-2**		Standard Orientation (Ångstroms)			
Center number	Atom number	Type	X	Y	Z
1.	6.	0.	2.005060	2.587236	-0.576060
2.	6.	0.	1.850072	2.942759	0.770653
3.	6.	0.	2.352890	2.101224	1.765818
4.	6.	0.	2.994787	0.906050	1.442517
5.	6.	0.	3.139597	0.538440	0.095936
6.	6.	0.	2.658406	1.388633	-0.900725
7.	6.	0.	3.772179	-0.796834	-0.278172
8.	8.	0.	1.551498	3.354009	-1.602944
9.	6.	0.	2.922310	-2.014434	0.130785
10.	8.	0.	5.035371	-0.999064	0.351849
11.	6.	0.	6.051810	-0.122825	-0.101082
12.	6.	0.	1.468558	-1.941567	-0.354976
13.	6.	0.	0.653677	-3.246618	-0.206005
14.	6.	0.	0.861350	-3.927022	1.158380
15.	6.	0.	-0.865376	-3.100293	-0.485572
16.	6.	0.	-1.322671	-2.554604	-1.851903
17.	6.	0.	-1.215745	-1.009606	-1.919987
18.	6.	0.	-2.751771	-3.035251	-2.156930
19.	6.	0.	-1.878285	1.061042	-0.584651
20.	6.	0.	-1.276386	2.053165	-1.274825
21.	6.	0.	-1.820007	-1.080028	0.510639
22.	6.	0.	-2.249029	-0.516601	1.867773
23.	6.	0.	-2.273772	1.040758	1.856447
24.	6.	0.	-2.696416	1.674339	0.526811
25.	6.	0.	-1.445697	3.293575	-0.522878
26.	8.	0.	-0.892394	4.373571	-0.672440
27.	8.	0.	-1.515073	-2.393121	0.610605
28.	6.	0.	-4.199469	1.637566	0.226548
29.	6.	0.	-1.234567	-0.947186	2.954647
30.	6.	0.	-1.724120	-0.372848	-0.650514
31.	6.	0.	-3.626611	-1.119664	2.242540
32.	8.	0.	-2.300574	3.072196	0.525416
33.	8.	0.	-1.966495	-0.467454	-3.011316
34.	1.	0.	1.342425	3.867813	1.026445
35.	1.	0.	2.243527	2.390418	2.807751
36.	1.	0.	3.395607	0.265008	2.221509
37.	1.	0.	2.769752	1.132524	-1.950990
38.	1.	0.	3.905481	-0.813092	-1.374458
39.	1.	0.	0.832888	3.946248	-1.292876

40.	1.	0.	3.422269	-2.908580	-0.262137
41.	1.	0.	2.959263	-2.088796	1.222653
42.	1.	0.	6.964870	-0.393330	0.434553
43.	1.	0.	6.228488	-0.233778	-1.182434
44.	1.	0.	5.813667	0.928879	0.105021
45.	1.	0.	1.477792	-1.654436	-1.413020
46.	1.	0.	0.966083	-1.129693	0.180281
47.	1.	0.	1.005249	-3.950028	-0.977056
48.	1.	0.	1.900793	-4.237116	1.287985
49.	1.	0.	0.603304	-3.255068	1.981071
50.	1.	0.	0.235329	-4.821226	1.249768
51.	1.	0.	-1.278501	-4.110485	-0.390776
52.	1.	0.	-0.648060	-2.976647	-2.609832
53.	1.	0.	-0.164329	-0.722468	-2.050830
54.	1.	0.	-3.449073	-2.719304	-1.375111
55.	1.	0.	-3.108677	-2.624608	-3.102091
56.	1.	0.	-2.781663	-4.127970	-2.214680
57.	1.	0.	-0.617138	1.983113	-2.125799
58.	1.	0.	-2.909718	1.399913	2.673105
59.	1.	0.	-1.260734	1.405558	2.056164
60.	1.	0.	-4.547142	0.616941	0.055833
61.	1.	0.	-4.761408	2.071399	1.058598
62.	1.	0.	-4.402947	2.219449	-0.675535
63.	1.	0.	-0.219874	-0.624708	2.700627
64.	1.	0.	-1.505772	-0.481945	3.908271
65.	1.	0.	-1.232553	-2.030065	3.090807
66.	1.	0.	-3.570164	-2.211339	2.251180
67.	1.	0.	-4.415041	-0.828597	1.545834
68.	1.	0.	-3.918754	-0.785278	3.243370
69.	1.	0.	-1.478552	-0.656223	-3.823846

**Isomer 2'.-3**		Standard Orientation (Ångstroms)			
Center number	Atom number	Type	X	Y	Z
1.	6.	0.	-7.646926	-0.372959	-0.054795
2.	6.	0.	-8.152552	0.932276	-0.055917
3.	6.	0.	-7.299181	1.992133	0.235974
4.	6.	0.	-5.951839	1.766137	0.529010
5.	6.	0.	-5.444351	0.463756	0.521151
6.	6.	0.	-6.298974	-0.605084	0.229959
7.	6.	0.	-3.964651	0.203262	0.773046
8.	8.	0.	-8.521085	-1.384564	-0.339807

9.	6.	0.	-3.172038	0.125872	-0.541497
10.	8.	0.	-3.739634	-1.024724	1.461137
11.	6.	0.	-4.099570	-0.991767	2.832057
12.	6.	0.	-1.660103	-0.011936	-0.319506
13.	6.	0.	-0.854824	-0.273770	-1.608143
14.	6.	0.	-0.977751	0.870376	-2.628832
15.	6.	0.	0.625534	-0.581539	-1.323403
16.	6.	0.	0.948422	-1.778097	-0.420496
17.	6.	0.	2.483869	-1.934651	-0.368227
18.	6.	0.	0.268055	-3.078673	-0.858712
19.	6.	0.	4.528410	-0.538633	0.307736
20.	6.	0.	5.328781	-1.419035	0.940771
21.	6.	0.	2.468623	0.570621	-0.287594
22.	6.	0.	2.969793	1.956699	0.122549
23.	6.	0.	4.406737	1.889273	0.724871
24.	6.	0.	5.278266	0.766766	0.150277
25.	6.	0.	6.556350	-0.725052	1.365984
26.	8.	0.	7.502282	-1.130238	1.996202
27.	8.	0.	1.200050	0.622587	-0.746066
28.	6.	0.	5.748089	0.992680	-1.294406
29.	6.	0.	2.017825	2.503076	1.217440
30.	6.	0.	3.156892	-0.601111	-0.146675
31.	6.	0.	2.904089	2.922222	-1.086358
32.	8.	0.	6.465700	0.596905	0.949378
33.	8.	0.	3.001824	-2.611509	-1.522153
34.	1.	0.	-9.202325	1.090497	-0.278380
35.	1.	0.	-7.690904	3.005251	0.243578
36.	1.	0.	-5.297381	2.601117	0.764725
37.	1.	0.	-5.902970	-1.618065	0.242918
38.	1.	0.	-3.562683	1.035365	1.376848
39.	1.	0.	-8.048515	-2.226788	-0.307406
40.	1.	0.	-3.551461	-0.731304	-1.113309
41.	1.	0.	-3.405626	1.021697	-1.126186
42.	1.	0.	-5.171954	-0.802257	2.972666
43.	1.	0.	-3.852994	-1.970805	3.249476
44.	1.	0.	-3.534516	-0.220402	3.376796
45.	1.	0.	-1.504004	-0.832443	0.387030
46.	1.	0.	-1.275204	0.895362	0.163580
47.	1.	0.	-1.256050	-1.180721	-2.080428
48.	1.	0.	-2.011479	0.998401	-2.960376
49.	1.	0.	-0.639291	1.818449	-2.200217
50.	1.	0.	-0.371373	0.669819	-3.518995
51.	1.	0.	1.132683	-0.740814	-2.289625

52.	1.	0.	0.621039	-1.527025	0.597194
53.	1.	0.	2.725820	-2.598712	0.470815
54.	1.	0.	0.518770	-3.326048	-1.893447
55.	1.	0.	0.611258	-3.910617	-0.235936
56.	1.	0.	-0.818864	-3.017124	-0.761593
57.	1.	0.	5.134580	-2.450283	1.200446
58.	1.	0.	4.899522	2.859655	0.601027
59.	1.	0.	4.337712	1.703789	1.801843
60.	1.	0.	4.910542	0.995796	-1.995321
61.	1.	0.	6.278315	1.946083	-1.372438
62.	1.	0.	6.433445	0.191316	-1.581519
63.	1.	0.	2.383569	3.472714	1.571043
64.	1.	0.	1.005376	2.637152	0.830141
65.	1.	0.	1.972872	1.824647	2.075106
66.	1.	0.	3.180256	3.931596	-0.765239
67.	1.	0.	1.890976	2.958247	-1.493088
68.	1.	0.	3.581264	2.631893	-1.892475
69.	1.	0.	3.204640	-1.940161	-2.186355

**Isomer 2'.-4**		Standard Orientation (Ångstroms)			
Center number	Atom number	Type	X	Y	Z
1.	6.	0.	-5.231067	-2.457850	0.245670
2.	6.	0.	-4.776650	-3.301851	-0.774133
3.	6.	0.	-3.985906	-2.774143	-1.790756
4.	6.	0.	-3.645726	-1.419105	-1.802452
5.	6.	0.	-4.091325	-0.577551	-0.778771
6.	6.	0.	-4.887549	-1.103447	0.244250
7.	6.	0.	-3.672702	0.886956	-0.737768
8.	8.	0.	-6.010843	-3.014858	1.220950
9.	6.	0.	-2.409122	1.084471	0.114635
10.	8.	0.	-4.683405	1.724386	-0.181758
11.	6.	0.	-5.792155	1.936624	-1.038757
12.	6.	0.	-1.889907	2.528343	0.076966
13.	6.	0.	-0.699857	2.876603	1.002894
14.	6.	0.	-1.017226	2.636684	2.488377
15.	6.	0.	0.656883	2.259425	0.625679
16.	6.	0.	1.152465	2.466114	-0.812797
17.	6.	0.	2.571840	1.884781	-0.904216
18.	6.	0.	1.120024	3.928469	-1.275449
19.	6.	0.	3.777829	-0.337818	-0.503690
20.	6.	0.	4.670599	-0.412438	-1.514283

21.	6.	0.	1.657301	0.071588	0.572287
22.	6.	0.	1.548696	-1.346368	1.137006
23.	6.	0.	2.748665	-2.234206	0.682265
24.	6.	0.	4.057135	-1.465858	0.466112
25.	6.	0.	5.461792	-1.645356	-1.359779
26.	8.	0.	6.325020	-2.108673	-2.064553
27.	8.	0.	0.592886	0.829239	0.908222
28.	6.	0.	4.733184	-0.969063	1.752615
29.	6.	0.	0.249204	-1.992870	0.594871
30.	6.	0.	2.654022	0.528899	-0.240174
31.	6.	0.	1.439302	-1.287282	2.680893
32.	8.	0.	5.020763	-2.294786	-0.215406
33.	8.	0.	3.467108	2.828176	-0.299376
34.	1.	0.	-5.056065	-4.349759	-0.754986
35.	1.	0.	-3.638631	-3.425042	-2.588063
36.	1.	0.	-3.036471	-1.017068	-2.607669
37.	1.	0.	-5.246140	-0.440885	1.028844
38.	1.	0.	-3.458808	1.215187	-1.770189
39.	1.	0.	-6.256745	-2.328964	1.855609
40.	1.	0.	-2.651772	0.786676	1.140952
41.	1.	0.	-1.645155	0.387856	-0.239828
42.	1.	0.	-6.476157	2.608189	-0.514472
43.	1.	0.	-5.484453	2.409651	-1.983594
44.	1.	0.	-6.318375	1.001646	-1.273156
45.	1.	0.	-2.720052	3.189942	0.346524
46.	1.	0.	-1.630087	2.781528	-0.959424
47.	1.	0.	-0.540396	3.957264	0.887864
48.	1.	0.	-1.948767	3.139127	2.767621
49.	1.	0.	-1.125395	1.573456	2.714664
50.	1.	0.	-0.218995	3.031245	3.126232
51.	1.	0.	1.421563	2.671625	1.299557
52.	1.	0.	0.518980	1.864595	-1.478261
53.	1.	0.	2.831044	1.769537	-1.969084
54.	1.	0.	1.682236	4.570212	-0.593246
55.	1.	0.	1.581720	4.019333	-2.263654
56.	1.	0.	0.097180	4.305810	-1.351697
57.	1.	0.	4.773898	0.223893	-2.382782
58.	1.	0.	2.894892	-3.044750	1.404276
59.	1.	0.	2.505457	-2.703816	-0.276679
60.	1.	0.	4.133263	-0.207167	2.254369
61.	1.	0.	4.894754	-1.804577	2.439389
62.	1.	0.	5.705165	-0.533982	1.505857
63.	1.	0.	0.234578	-1.995031	-0.499584

64.	1.	0.	0.188312	-3.032147	0.934312
65.	1.	0.	-0.638086	-1.465745	0.950554
66.	1.	0.	0.596138	-0.660703	2.980088
67.	1.	0.	2.340456	-0.887750	3.150928
68.	1.	0.	1.273509	-2.294707	3.075650
69.	1.	0.	4.326675	2.394961	-0.210545

**Isomer 2'.-5**		Standard Orientation (Ångstroms)			
Center number	Atom number	Type	X	Y	Z
1.	6.	0.	-3.325234	2.894862	-0.192937
2.	6.	0.	-4.166009	3.124375	0.902960
3.	6.	0.	-4.932236	2.075903	1.409454
4.	6.	0.	-4.865476	0.806113	0.834969
5.	6.	0.	-4.016460	0.572152	-0.254212
6.	6.	0.	-3.251344	1.623413	-0.767634
7.	6.	0.	-3.894510	-0.815768	-0.872555
8.	8.	0.	-2.552471	3.877319	-0.746076
9.	6.	0.	-2.590734	-1.530593	-0.481470
10.	8.	0.	-3.887314	-0.769067	-2.297489
11.	6.	0.	-5.127752	-0.397087	-2.871220
12.	6.	0.	-2.480680	-1.835603	1.017789
13.	6.	0.	-1.203398	-2.573422	1.488417
14.	6.	0.	-1.023325	-3.941338	0.810500
15.	6.	0.	0.099312	-1.756309	1.440673
16.	6.	0.	0.083507	-0.369624	2.100933
17.	6.	0.	1.511746	0.194839	2.039068
18.	6.	0.	-0.446718	-0.379156	3.540910
19.	6.	0.	3.308821	0.725012	0.295548
20.	6.	0.	3.854331	1.882399	0.727938
21.	6.	0.	1.568660	-0.857807	-0.239577
22.	6.	0.	2.000604	-1.002938	-1.700050
23.	6.	0.	3.198270	-0.061123	-2.035889
24.	6.	0.	4.141477	0.205477	-0.857117
25.	6.	0.	4.945239	2.264563	-0.185078
26.	8.	0.	5.653477	3.241960	-0.184285
27.	8.	0.	0.492009	-1.619491	0.043012
28.	6.	0.	5.013909	-0.989652	-0.446207
29.	6.	0.	0.810641	-0.597536	-2.605889
30.	6.	0.	2.129327	-0.014659	0.675328
31.	6.	0.	2.341603	-2.483983	-1.998598
32.	8.	0.	5.041610	1.288722	-1.167117

33.	8.	0.	2.277647	-0.443864	3.070731
34.	1.	0.	-4.224069	4.115280	1.348330
35.	1.	0.	-5.591126	2.256095	2.253962
36.	1.	0.	-5.474806	-0.002331	1.229249
37.	1.	0.	-2.610019	1.467522	-1.628612
38.	1.	0.	-4.752352	-1.422286	-0.532275
39.	1.	0.	-2.700714	4.704438	-0.269140
40.	1.	0.	-2.550034	-2.454937	-1.067271
41.	1.	0.	-1.751759	-0.911840	-0.809519
42.	1.	0.	-5.001498	-0.442493	-3.955466
43.	1.	0.	-5.931216	-1.090505	-2.577908
44.	1.	0.	-5.427551	0.621259	-2.589892
45.	1.	0.	-3.337129	-2.457878	1.313486
46.	1.	0.	-2.586157	-0.902845	1.581860
47.	1.	0.	-1.351071	-2.769260	2.558961
48.	1.	0.	-1.926501	-4.549106	0.927582
49.	1.	0.	-0.818802	-3.842096	-0.257987
50.	1.	0.	-0.189601	-4.492667	1.258487
51.	1.	0.	0.891319	-2.344997	1.925524
52.	1.	0.	-0.547204	0.289235	1.489735
53.	1.	0.	1.463774	1.277334	2.239596
54.	1.	0.	0.118105	-1.076368	4.164164
55.	1.	0.	-0.341684	0.615449	3.985505
56.	1.	0.	-1.505054	-0.648766	3.579404
57.	1.	0.	3.530802	2.531734	1.530010
58.	1.	0.	3.753771	-0.469820	-2.886677
59.	1.	0.	2.811799	0.914750	-2.347806
60.	1.	0.	4.412861	-1.806366	-0.041432
61.	1.	0.	5.579811	-1.356559	-1.307081
62.	1.	0.	5.723685	-0.671736	0.321951
63.	1.	0.	0.477786	0.421934	-2.386710
64.	1.	0.	1.120945	-0.632722	-3.655536
65.	1.	0.	-0.037850	-1.272615	-2.477942
66.	1.	0.	1.490712	-3.126841	-1.762390
67.	1.	0.	3.202053	-2.840122	-1.427867
68.	1.	0.	2.573035	-2.600555	-3.062205
69.	1.	0.	3.204637	-0.209035	2.929389

1.3.2 Methods for NMR calculation of Neo-debromoaplysiatoxin E (1) and Neo-debromoaplysiatoxin F (2)

Monte Carlo conformational searches were carried out by means of the Spartan's 10 software (Spartan Software, San Francisco, CA, USA) using Merck Molecular Force Field (MMFF). The

conformers with Boltzmann-population of over 1% were chosen for NMR calculations, and then the conformers were initially optimized at B3LYP/6-31g (d, p) level in gas. Meanwhile, gauge-independent atomic orbital (GIAO) calculations of ^{1}H and ^{13}C NMR chemical shifts were accomplished by density functional theory (DFT) at the mPWLPW91-SCRF (methanol)/6-311+g (d,p) level with the PCM solvent continuum model in Gaussian 09 software (Gaussian, Wallingford, CT, USA). The calculated NMR data of the lowest energy conformers for **isomer 1**, **isomer 1'**, **isomer 2**, **isomer 2'** were averaged according to the Boltzmann distribution theory and their relative Gibbs free energy. The ^{1}H and ^{13}C NMR chemical shifts for TMS were calculated by the same protocol and used as reference. The experimental and calculated data were analyzed by the improved probability DP4+ method for isomeric compounds. A significant higher DP4+ probability score suggested the correctness of its configuration.

Table S1.3.2.1.a. Gibbs free energies[a] and equilibrium populations[b] of low-energy conformers of **isomer 1**.

Conformers	In MeOH	
	G	*P* (%)
**isomer 1.-1**	-942154.77090189	91.34
**isomer 1.-2**	-942153.02893413	4.82
**isomer 1.-3**	-942152.60222733	2.34
**isomer 1.-4**	-942152.33616309	1.49

[a]B3LYP/6-31G (d,p), in kcal/mol. [b]From G values at 298.15K.

Table S1.3.2.1.b. Cartesian coordinates for the low-energy reoptimized MMFF conformers of **isomer 1** at B3LYP/6-31G (d,p) level of theory in CH_3OH.

**Isomer 1.-1**		Standard Orientation (Ångstroms)			
Center number	Atomic number	Atomic Type	X	Y	Z
1.	6.	0.	-7.446100	1.320266	0.173006
2.	6.	0.	-8.209157	0.193408	-0.147624
3.	6.	0.	-7.602717	-1.063483	-0.161605
4.	6.	0.	-6.248314	-1.204345	0.133340
5.	6.	0.	-5.483376	-0.074077	0.453583
6.	6.	0.	-6.087881	1.183383	0.477726
7.	6.	0.	-3.989392	-0.207338	0.723475
8.	8.	0.	-7.971973	2.582053	0.213431
9.	6.	0.	-3.179645	-0.210657	-0.582632
10.	8.	0.	-3.668962	-1.414787	1.408673
11.	6.	0.	-4.066103	-1.429099	2.768978

12.	6.	0.	-1.663641	-0.236499	-0.347170
13.	6.	0.	-0.830860	-0.420855	-1.631844
14.	6.	0.	-1.014615	0.735479	-2.629322
15.	6.	0.	0.662116	-0.647558	-1.343368
16.	6.	0.	1.040243	-1.838247	-0.450798
17.	6.	0.	2.573502	-1.908787	-0.396156
18.	6.	0.	0.426735	-3.167437	-0.906569
19.	6.	0.	4.532225	-0.419077	0.323817
20.	6.	0.	5.380628	-1.266897	0.944935
21.	6.	0.	2.425447	0.592520	-0.270663
22.	6.	0.	2.853404	1.995859	0.162777
23.	6.	0.	4.292741	1.993349	0.764269
24.	6.	0.	5.219014	0.922268	0.176652
25.	6.	0.	6.574175	-0.517545	1.372358
26.	8.	0.	7.542216	-0.883997	1.993126
27.	8.	0.	1.161545	0.580977	-0.736208
28.	6.	0.	5.672202	1.184771	-1.267017
29.	6.	0.	1.874303	2.476573	1.264036
30.	6.	0.	3.170820	-0.543520	-0.137231
31.	6.	0.	2.739388	2.973863	-1.032492
32.	8.	0.	6.415978	0.802000	0.971716
33.	8.	0.	3.028599	-2.468257	-1.635902
34.	1.	0.	-9.267259	0.298420	-0.378070
35.	1.	0.	-8.198421	-1.938476	-0.405694
36.	1.	0.	-5.777100	-2.181302	0.132250
37.	1.	0.	-5.520770	2.073740	0.734342
38.	1.	0.	-3.667051	0.652988	1.335990
39.	1.	0.	-8.911861	2.538729	-0.006196
40.	1.	0.	-3.487131	-1.088296	-1.166381
41.	1.	0.	-3.474421	0.670948	-1.161508
42.	1.	0.	-3.735815	-2.382992	3.186779
43.	1.	0.	-3.594339	-0.610625	3.333792
44.	1.	0.	-5.155276	-1.347651	2.881338
45.	1.	0.	-1.452934	-1.051769	0.351026
46.	1.	0.	-1.352668	0.690827	0.151329
47.	1.	0.	-1.172864	-1.339550	-2.127717
48.	1.	0.	-0.392409	0.588656	-3.518751
49.	1.	0.	-2.052471	0.810628	-2.964483
50.	1.	0.	-0.733878	1.692081	-2.178380
51.	1.	0.	1.194979	-0.764818	-2.297389
52.	1.	0.	0.699634	-1.618732	0.569148
53.	1.	0.	2.859320	-2.577812	0.431507
54.	1.	0.	-0.660582	-3.164049	-0.795192

55.	1.	0.	0.675088	-3.381495	-1.948968
56.	1.	0.	0.819923	-3.989943	-0.300914
57.	1.	0.	5.233261	-2.303618	1.215492
58.	1.	0.	4.737032	2.987920	0.650379
59.	1.	0.	4.234182	1.793198	1.839217
60.	1.	0.	4.834731	1.143202	-1.966422
61.	1.	0.	6.147976	2.166877	-1.338849
62.	1.	0.	6.402805	0.426411	-1.560248
63.	1.	0.	0.856437	2.561603	0.877124
64.	1.	0.	1.864962	1.785079	2.112390
65.	1.	0.	2.187664	3.459559	1.631104
66.	1.	0.	2.964306	3.991295	-0.696832
67.	1.	0.	1.725857	2.963873	-1.439349
68.	1.	0.	3.429767	2.728674	-1.842336
69.	1.	0.	3.989249	-2.365404	-1.664985

**Isomer 1.-2**		Standard Orientation (Ångstroms)			
Center number	Atom number	Type	X	Y	Z
1.	6.	0.	-4.105261	-3.008058	-0.993004
2.	6.	0.	-4.804414	-3.383692	0.158163
3.	6.	0.	-5.192562	-2.404430	1.073762
4.	6.	0.	-4.885815	-1.062900	0.855635
5.	6.	0.	-4.183783	-0.686727	-0.298154
6.	6.	0.	-3.801727	-1.661692	-1.220527
7.	6.	0.	-3.792293	0.769525	-0.519820
8.	8.	0.	-3.697242	-3.908665	-1.937714
9.	6.	0.	-2.490760	1.117309	0.219395
10.	8.	0.	-4.789155	1.674008	-0.053222
11.	6.	0.	-5.955080	1.711943	-0.857355
12.	6.	0.	-2.006650	2.542915	-0.080347
13.	6.	0.	-0.765343	3.048183	0.693186
14.	6.	0.	-0.982943	3.059307	2.215531
15.	6.	0.	0.573935	2.383335	0.335497
16.	6.	0.	0.964945	2.333407	-1.148629
17.	6.	0.	2.384373	1.750482	-1.237966
18.	6.	0.	0.875528	3.690733	-1.858101
19.	6.	0.	3.661912	-0.351538	-0.525429
20.	6.	0.	4.488806	-0.600614	-1.563864
21.	6.	0.	1.609959	0.229070	0.603343
22.	6.	0.	1.569558	-1.062483	1.422844
23.	6.	0.	2.753575	-2.008644	1.052976

24.	6.	0.	4.028535	-1.281021	0.611737
25.	6.	0.	5.315154	-1.776912	-1.242174
26.	8.	0.	6.141536	-2.352220	-1.907583
27.	8.	0.	0.556132	1.026323	0.870984
28.	6.	0.	4.777136	-0.551578	1.737026
29.	6.	0.	0.248951	-1.806776	1.102846
30.	6.	0.	2.540260	0.538918	-0.346150
31.	6.	0.	1.566139	-0.722008	2.933892
32.	8.	0.	4.963007	-2.211488	0.027962
33.	8.	0.	3.304445	2.792724	-0.884446
34.	1.	0.	-5.047262	-4.429770	0.332460
35.	1.	0.	-5.740704	-2.697321	1.964767
36.	1.	0.	-5.195159	-0.299422	1.561330
37.	1.	0.	-3.267788	-1.396353	-2.128712
38.	1.	0.	-3.638279	0.928778	-1.601809
39.	1.	0.	-3.975425	-4.794554	-1.670595
40.	1.	0.	-2.673223	0.986639	1.292045
41.	1.	0.	-1.731748	0.382205	-0.060642
42.	1.	0.	-6.621569	2.457093	-0.416676
43.	1.	0.	-5.721274	2.010700	-1.890672
44.	1.	0.	-6.470448	0.742577	-0.883496
45.	1.	0.	-2.831451	3.229694	0.137590
46.	1.	0.	-1.822186	2.631219	-1.159166
47.	1.	0.	-0.631124	4.095211	0.389557
48.	1.	0.	-0.147274	3.550663	2.725586
49.	1.	0.	-1.897606	3.605017	2.467950
50.	1.	0.	-1.069847	2.048756	2.621439
51.	1.	0.	1.375278	2.913751	0.869637
52.	1.	0.	0.296493	1.621112	-1.650265
53.	1.	0.	2.567271	1.442815	-2.280255
54.	1.	0.	-0.155883	4.046671	-1.919601
55.	1.	0.	1.478191	4.443700	-1.345113
56.	1.	0.	1.259638	3.606185	-2.879569
57.	1.	0.	4.521665	-0.132322	-2.538258
58.	1.	0.	2.964774	-2.672998	1.897798
59.	1.	0.	2.457104	-2.646359	0.213554
60.	1.	0.	4.195815	0.284183	2.131399
61.	1.	0.	5.000819	-1.246021	2.551715
62.	1.	0.	5.721337	-0.160799	1.348887
63.	1.	0.	-0.621663	-1.230111	1.421216
64.	1.	0.	0.156891	-2.008880	0.031108
65.	1.	0.	0.234554	-2.766907	1.629285
66.	1.	0.	0.730724	-0.060597	3.173702

67.	1.	0.	2.487338	-0.232098	3.256414
68.	1.	0.	1.453289	-1.641600	3.516974
69.	1.	0.	4.176155	2.387362	-0.783310

**Isomer 1.-3**		Standard Orientation (Ångstroms)			
Center number	Atom number	Type	X	Y	Z
1.	6.	0.	-5.738577	-1.881399	1.366392
2.	6.	0.	-5.500134	-2.844569	0.377246
3.	6.	0.	-4.900419	-2.458437	-0.816282
4.	6.	0.	-4.531529	-1.126320	-1.032954
5.	6.	0.	-4.752300	-0.166884	-0.040997
6.	6.	0.	-5.367143	-0.551814	1.157125
7.	6.	0.	-4.338967	1.285837	-0.238192
8.	8.	0.	-6.342680	-2.303200	2.516943
9.	6.	0.	-3.149953	1.706398	0.642106
10.	8.	0.	-5.390244	2.187664	0.108544
11.	6.	0.	-6.492350	2.160204	-0.780977
12.	6.	0.	-1.824391	0.964333	0.411164
13.	6.	0.	-1.174801	1.127655	-0.982304
14.	6.	0.	-1.077210	2.595870	-1.431885
15.	6.	0.	0.212033	0.461981	-1.053155
16.	6.	0.	0.303152	-1.034788	-0.725715
17.	6.	0.	1.760963	-1.473008	-0.933936
18.	6.	0.	-0.664399	-1.900576	-1.541340
19.	6.	0.	4.082809	-0.890957	-0.014941
20.	6.	0.	4.688254	-2.088692	0.136719
21.	6.	0.	2.311647	0.742073	0.102431
22.	6.	0.	3.130964	1.731023	0.933985
23.	6.	0.	4.536010	1.152414	1.286230
24.	6.	0.	5.106615	0.196839	0.231955
25.	6.	0.	6.057791	-1.870327	0.632617
26.	8.	0.	6.910303	-2.667500	0.939685
27.	8.	0.	1.071229	1.208153	-0.140764
28.	6.	0.	5.559541	0.871242	-1.071558
29.	6.	0.	2.373611	1.989079	2.261421
30.	6.	0.	2.721361	-0.499281	-0.289698
31.	6.	0.	3.244312	3.080423	0.182891
32.	8.	0.	6.254049	-0.501790	0.756065
33.	8.	0.	1.983439	-1.588787	-2.346322
34.	1.	0.	-5.797878	-3.871467	0.559922
35.	1.	0.	-4.726649	-3.199708	-1.590906

36.	1.	0.	-4.084355	-0.833044	-1.978758
37.	1.	0.	-5.573335	0.200728	1.915312
38.	1.	0.	-4.079702	1.427432	-1.301056
39.	1.	0.	-6.452457	-1.548548	3.110472
40.	1.	0.	-3.018399	2.784804	0.507332
41.	1.	0.	-3.448688	1.566080	1.687625
42.	1.	0.	-7.200265	2.915479	-0.431652
43.	1.	0.	-6.186738	2.409173	-1.809024
44.	1.	0.	-6.991540	1.181997	-0.795054
45.	1.	0.	-1.978690	-0.101316	0.615874
46.	1.	0.	-1.110776	1.321577	1.162414
47.	1.	0.	-1.787326	0.597124	-1.722629
48.	1.	0.	-0.549996	2.674466	-2.388813
49.	1.	0.	-2.066699	3.040945	-1.565262
50.	1.	0.	-0.532142	3.198845	-0.699779
51.	1.	0.	0.624506	0.607844	-2.061111
52.	1.	0.	0.086172	-1.163377	0.342378
53.	1.	0.	1.893729	-2.459883	-0.462176
54.	1.	0.	-1.704824	-1.704092	-1.271951
55.	1.	0.	-0.535389	-1.732619	-2.613547
56.	1.	0.	-0.468218	-2.960619	-1.351749
57.	1.	0.	4.271584	-3.081720	0.037196
58.	1.	0.	5.232758	1.976574	1.472823
59.	1.	0.	4.466300	0.578449	2.216169
60.	1.	0.	4.717316	1.298854	-1.619484
61.	1.	0.	6.281217	1.663506	-0.853442
62.	1.	0.	6.045237	0.129888	-1.711343
63.	1.	0.	1.408002	2.465923	2.079293
64.	1.	0.	2.201549	1.054994	2.805418
65.	1.	0.	2.968433	2.648977	2.901575
66.	1.	0.	2.251825	3.471695	-0.051408
67.	1.	0.	3.802189	2.993951	-0.751869
68.	1.	0.	3.760185	3.810598	0.814685
69.	1.	0.	2.932953	-1.709729	-2.480763

**Isomer 1.-4**		Standard Orientation (Ångstroms)			
Center number	Atom number	Type	X	Y	Z
1.	6.	0.	-3.555156	2.863935	-0.244384
2.	6.	0.	-4.420117	3.045189	0.842380
3.	6.	0.	-5.113986	1.951262	1.348572
4.	6.	0.	-4.955473	0.680751	0.786416

5.	6.	0.	-4.082958	0.494957	-0.289474
6.	6.	0.	-3.386737	1.595866	-0.804904
7.	6.	0.	-3.853722	-0.885983	-0.891299
8.	8.	0.	-2.902987	3.968767	-0.715185
9.	6.	0.	-2.523827	-1.520167	-0.451031
10.	8.	0.	-3.801691	-0.845719	-2.316497
11.	6.	0.	-5.048736	-0.572457	-2.931359
12.	6.	0.	-2.441089	-1.790595	1.056702
13.	6.	0.	-1.163742	-2.496234	1.573500
14.	6.	0.	-0.966525	-3.891936	0.959625
15.	6.	0.	0.133867	-1.673043	1.503306
16.	6.	0.	0.101818	-0.257000	2.096699
17.	6.	0.	1.525685	0.316677	2.020356
18.	6.	0.	-0.440591	-0.203302	3.531180
19.	6.	0.	3.336105	0.777394	0.269745
20.	6.	0.	3.863624	1.963006	0.643763
21.	6.	0.	1.616939	-0.847117	-0.202667
22.	6.	0.	2.071548	-1.067294	-1.647141
23.	6.	0.	3.269231	-0.137748	-2.016438
24.	6.	0.	4.190861	0.201978	-0.839411
25.	6.	0.	4.965552	2.303742	-0.272731
26.	8.	0.	5.663901	3.286959	-0.317559
27.	8.	0.	0.541989	-1.601661	0.104592
28.	6.	0.	5.065210	-0.961385	-0.349765
29.	6.	0.	0.892485	-0.720114	-2.590386
30.	6.	0.	2.158167	0.047637	0.673789
31.	6.	0.	2.425462	-2.560146	-1.860147
32.	8.	0.	5.086854	1.274223	-1.195648
33.	8.	0.	2.287556	-0.267176	3.086467
34.	1.	0.	-4.537155	4.038691	1.261745
35.	1.	0.	-5.792639	2.090804	2.185148
36.	1.	0.	-5.511873	-0.164054	1.182777
37.	1.	0.	-2.731484	1.454609	-1.661876
38.	1.	0.	-4.683199	-1.542558	-0.575440
39.	1.	0.	-2.350675	3.713771	-1.465994
40.	1.	0.	-2.416007	-2.451246	-1.017433
41.	1.	0.	-1.709738	-0.863158	-0.768569
42.	1.	0.	-4.883926	-0.604035	-4.010971
43.	1.	0.	-5.802718	-1.329145	-2.665332
44.	1.	0.	-5.438700	0.417186	-2.658162
45.	1.	0.	-3.293137	-2.421135	1.347475
46.	1.	0.	-2.576639	-0.847467	1.596486
47.	1.	0.	-1.322930	-2.645629	2.649946

48.	1.	0.	-0.129128	-4.413410	1.435590
49.	1.	0.	-1.863972	-4.502836	1.101353
50.	1.	0.	-0.758234	-3.840417	-0.111495
51.	1.	0.	0.923831	-2.232900	2.024161
52.	1.	0.	-0.529520	0.367572	1.451161
53.	1.	0.	1.466724	1.406805	2.169929
54.	1.	0.	-1.497081	-0.479165	3.573711
55.	1.	0.	0.124411	-0.865846	4.190892
56.	1.	0.	-0.347323	0.811936	3.928922
57.	1.	0.	3.522320	2.652977	1.403520
58.	1.	0.	3.841740	-0.590551	-2.832879
59.	1.	0.	2.882667	0.816159	-2.390135
60.	1.	0.	4.462873	-1.761356	0.085361
61.	1.	0.	5.651149	-1.367136	-1.179232
62.	1.	0.	5.757131	-0.597552	0.414325
63.	1.	0.	0.042515	-1.386617	-2.431355
64.	1.	0.	0.556894	0.310630	-2.436715
65.	1.	0.	1.215059	-0.817238	-3.632378
66.	1.	0.	1.574572	-3.194931	-1.603307
67.	1.	0.	3.278436	-2.879471	-1.257285
68.	1.	0.	2.675042	-2.732203	-2.912033
69.	1.	0.	3.212369	-0.021474	2.949352

Table S1.3.2.2.a. Gibbs free energies[a] and equilibrium populations[b] of low-energy conformers of **isomer 2**.

Conformers	In MeOH	
	G	*P* (%)
**isomer 2.-1**	-942153.68781963	41.60
**isomer 2.-2**	-942153.51901944	31.28
**isomer 2.-3**	-942152.88523434	10.72
**isomer 2.-4**	-942152.7195717	8.32
**isomer 2.-5**	-942152.71768917	8.08

[a]B3LYP/6-31G (d,p), in kcal/mol. [b]From G values at 298.15K.

Table S1.3.2.2.b. Cartesian coordinates for the low-energy reoptimized MMFF conformers of **isomer 2** at B3LYP/6-31G(d,p) level of theory in CH_3OH.

**Isomer 2.-1**		Standard Orientation (Ångstroms)			
Center number	Atomic number	Atomic Type	X	Y	Z
1.	6.	0.	4.218242	2.664886	-1.023380
2.	6.	0.	5.094666	3.090010	-0.021576
3.	6.	0.	5.746064	2.139594	0.767494
4.	6.	0.	5.524976	0.778723	0.571862

5.	6.	0.	4.641845	0.350713	-0.431027
6.	6.	0.	3.999056	1.297594	-1.229257
7.	6.	0.	4.371110	-1.136845	-0.622503
8.	8.	0.	3.548802	3.533382	-1.840188
9.	6.	0.	3.440696	-1.720082	0.454579
10.	8.	0.	5.566204	-1.910115	-0.552834
11.	6.	0.	6.441462	-1.721647	-1.650796
12.	6.	0.	2.012706	-1.163174	0.408651
13.	6.	0.	1.118014	-1.630144	1.575447
14.	6.	0.	0.977525	-3.160246	1.645860
15.	6.	0.	-0.269730	-0.964240	1.551896
16.	6.	0.	-0.322963	0.567201	1.593209
17.	6.	0.	-1.804143	1.005310	1.606417
18.	6.	0.	0.445756	1.169036	2.774278
19.	6.	0.	-3.882203	0.754339	0.121405
20.	6.	0.	-4.828951	1.492476	0.731126
21.	6.	0.	-2.123600	-0.893659	0.003885
22.	6.	0.	-2.854932	-1.748126	-1.034608
23.	6.	0.	-4.267757	-1.172590	-1.365899
24.	6.	0.	-4.381154	0.351952	-1.247327
25.	6.	0.	-6.034170	1.507524	-0.117983
26.	8.	0.	-7.113156	2.014331	0.068749
27.	8.	0.	-0.947347	-1.455900	0.362801
28.	6.	0.	-3.685979	1.135681	-2.370056
29.	6.	0.	-3.050778	-3.168507	-0.447922
30.	6.	0.	-2.588830	0.265149	0.551150
31.	6.	0.	-1.978377	-1.875494	-2.305428
32.	8.	0.	-5.765937	0.754135	-1.254317
33.	8.	0.	-2.406848	0.884506	2.905211
34.	1.	0.	5.270838	4.151950	0.135877
35.	1.	0.	6.433045	2.471826	1.540588
36.	1.	0.	6.043214	0.040589	1.174974
37.	1.	0.	3.323928	0.994465	-2.024262
38.	1.	0.	3.900522	-1.280828	-1.610943
39.	1.	0.	3.796551	4.438588	-1.610198
40.	1.	0.	3.441164	-2.805373	0.312257
41.	1.	0.	3.890925	-1.528657	1.437178
42.	1.	0.	5.952433	-1.976562	-2.603668
43.	1.	0.	6.811450	-0.689681	-1.714974
44.	1.	0.	7.289098	-2.393896	-1.498808
45.	1.	0.	1.536509	-1.445352	-0.539141
46.	1.	0.	2.065082	-0.069074	0.412963
47.	1.	0.	1.584661	-1.299288	2.513008

48.	1.	0.	0.577488	-3.563457	0.710918
49.	1.	0.	0.299185	-3.454056	2.454428
50.	1.	0.	1.941120	-3.638907	1.836988
51.	1.	0.	-0.841285	-1.345203	2.414769
52.	1.	0.	0.104210	0.946758	0.655757
53.	1.	0.	-1.842478	2.079691	1.392128
54.	1.	0.	0.301794	2.253653	2.801582
55.	1.	0.	0.080856	0.771172	3.724878
56.	1.	0.	1.519439	0.978581	2.699215
57.	1.	0.	-4.801558	1.932057	1.718166
58.	1.	0.	-4.569290	-1.503142	-2.365858
59.	1.	0.	-4.996330	-1.588735	-0.662166
60.	1.	0.	-4.059356	0.807049	-3.344160
61.	1.	0.	-3.903904	2.200592	-2.257073
62.	1.	0.	-2.602377	1.003554	-2.342577
63.	1.	0.	-2.092009	-3.655310	-0.258129
64.	1.	0.	-3.611627	-3.132858	0.491695
65.	1.	0.	-3.617433	-3.783667	-1.154685
66.	1.	0.	-0.998833	-2.289207	-2.054308
67.	1.	0.	-2.461369	-2.550426	-3.019345
68.	1.	0.	-1.822115	-0.916207	-2.803554
69.	1.	0.	-2.748613	-0.015958	2.984117

**Isomer 2.-2**		Standard Orientation (Ångstroms)			
Center number	Atom number	Type	X	Y	Z
1.	6.	0.	-5.636059	-2.201021	-0.249991
2.	6.	0.	-4.936525	-2.858756	-1.269701
3.	6.	0.	-4.037559	-2.139272	-2.049921
4.	6.	0.	-3.827272	-0.775049	-1.825824
5.	6.	0.	-4.514570	-0.119966	-0.799997
6.	6.	0.	-5.424182	-0.840680	-0.016241
7.	6.	0.	-4.271515	1.354200	-0.500815
8.	8.	0.	-6.516727	-2.943849	0.485094
9.	6.	0.	-3.361983	1.578687	0.719539
10.	8.	0.	-5.482823	2.046465	-0.205909
11.	6.	0.	-6.333747	2.221392	-1.325623
12.	6.	0.	-1.927147	1.071841	0.529696
13.	6.	0.	-1.055760	1.179481	1.798098
14.	6.	0.	-0.888900	2.629128	2.283720
15.	6.	0.	0.319475	0.509576	1.629235
16.	6.	0.	0.347591	-0.978601	1.262069

17.	6.	0.	1.821185	-1.437968	1.209025
18.	6.	0.	-0.474863	-1.855338	2.212106
19.	6.	0.	3.968452	-0.841375	-0.061727
20.	6.	0.	4.879997	-1.731086	0.374413
21.	6.	0.	2.229412	0.812546	0.184376
22.	6.	0.	3.014467	1.896204	-0.558376
23.	6.	0.	4.438164	1.402492	-0.967086
24.	6.	0.	4.537208	-0.101159	-1.250992
25.	6.	0.	6.125840	-1.543888	-0.391733
26.	8.	0.	7.191549	-2.101252	-0.295697
27.	8.	0.	1.042134	1.282487	0.631910
28.	6.	0.	3.894433	-0.547964	-2.572339
29.	6.	0.	3.191929	3.108772	0.389509
30.	6.	0.	2.658487	-0.458863	0.423244
31.	6.	0.	2.199017	2.369480	-1.787325
32.	8.	0.	5.918135	-0.512903	-1.299965
33.	8.	0.	2.372710	-1.686871	2.512293
34.	1.	0.	-5.116649	-3.915527	-1.435078
35.	1.	0.	-3.500972	-2.644319	-2.848002
36.	1.	0.	-3.132652	-0.220953	-2.451164
37.	1.	0.	-5.979192	-0.322772	0.763183
38.	1.	0.	-3.795489	1.812999	-1.384798
39.	1.	0.	-6.932867	-2.375126	1.146275
40.	1.	0.	-3.372963	2.653988	0.923944
41.	1.	0.	-3.823923	1.086877	1.585475
42.	1.	0.	-5.834374	2.790535	-2.124663
43.	1.	0.	-6.674364	1.263929	-1.741851
44.	1.	0.	-7.201378	2.787599	-0.978999
45.	1.	0.	-1.442304	1.626995	-0.283680
46.	1.	0.	-1.966946	0.025954	0.207457
47.	1.	0.	-1.555806	0.619447	2.599829
48.	1.	0.	-0.443837	3.256315	1.505669
49.	1.	0.	-0.238271	2.675135	3.163863
50.	1.	0.	-1.849854	3.067530	2.564430
51.	1.	0.	0.870900	0.631969	2.576485
52.	1.	0.	-0.046799	-1.084434	0.242776
53.	1.	0.	1.854832	-2.415092	0.713221
54.	1.	0.	-0.338476	-2.911609	1.960522
55.	1.	0.	-0.149797	-1.725761	3.247803
56.	1.	0.	-1.543247	-1.634689	2.145421
57.	1.	0.	4.800883	-2.414677	1.207855
58.	1.	0.	4.789051	1.980562	-1.828848
59.	1.	0.	5.134204	1.604604	-0.146175

60.	1.	0.	4.318784	0.017935	-3.406564
61.	1.	0.	4.099653	-1.609068	-2.734341
62.	1.	0.	2.811600	-0.406521	-2.562443
63.	1.	0.	2.229226	3.550732	0.654530
64.	1.	0.	3.703031	2.815986	1.312421
65.	1.	0.	3.800205	3.874744	-0.102735
66.	1.	0.	1.211635	2.720552	-1.478084
67.	1.	0.	2.718186	3.199420	-2.277358
68.	1.	0.	2.059803	1.577563	-2.526335
69.	1.	0.	2.724903	-0.850318	2.844111

**Isomer 2.-3**		Standard Orientation (Ångstroms)			
Center number	Atom number	Type	X	Y	Z
1.	6.	0.	5.807984	-1.832571	0.380174
2.	6.	0.	5.200619	-2.942140	-0.215518
3.	6.	0.	4.047234	-2.763029	-0.980659
4.	6.	0.	3.494084	-1.495417	-1.150073
5.	6.	0.	4.102659	-0.383185	-0.551438
6.	6.	0.	5.260965	-0.556922	0.206848
7.	6.	0.	3.478547	1.000394	-0.683781
8.	8.	0.	6.942754	-1.928139	1.137061
9.	6.	0.	2.364551	1.219538	0.351394
10.	8.	0.	2.885733	1.204977	-1.964764
11.	6.	0.	3.822499	1.368774	-3.015748
12.	6.	0.	1.825066	2.656898	0.331620
13.	6.	0.	0.620953	2.969475	1.250314
14.	6.	0.	0.927046	2.723065	2.736533
15.	6.	0.	-0.712471	2.315702	0.841374
16.	6.	0.	-1.150927	2.479385	-0.618338
17.	6.	0.	-2.532089	1.812906	-0.798241
18.	6.	0.	-1.186585	3.941304	-1.079476
19.	6.	0.	-3.622183	-0.484200	-0.472001
20.	6.	0.	-4.886746	-0.321494	-0.904930
21.	6.	0.	-1.645351	0.087166	0.788463
22.	6.	0.	-1.624426	-1.241100	1.550123
23.	6.	0.	-2.851334	-2.135584	1.185024
24.	6.	0.	-3.367493	-1.957259	-0.247854
25.	6.	0.	-5.570489	-1.627102	-0.864834
26.	8.	0.	-6.711126	-1.918436	-1.129699
27.	8.	0.	-0.644133	0.904449	1.190962
28.	6.	0.	-2.465754	-2.559756	-1.334728

29.	6.	0.	-1.701941	-0.933731	3.066621
30.	6.	0.	-2.566916	0.453067	-0.146476
31.	6.	0.	-0.286908	-1.974878	1.282799
32.	8.	0.	-4.664460	-2.569923	-0.395314
33.	8.	0.	-3.613728	2.644140	-0.344502
34.	1.	0.	5.629294	-3.933794	-0.087275
35.	1.	0.	3.580665	-3.625324	-1.448507
36.	1.	0.	2.604838	-1.352454	-1.754582
37.	1.	0.	5.759142	0.289113	0.671843
38.	1.	0.	4.267418	1.756576	-0.525593
39.	1.	0.	7.222571	-2.852306	1.171150
40.	1.	0.	1.565772	0.502882	0.141712
41.	1.	0.	2.762358	0.966476	1.340284
42.	1.	0.	4.480060	2.232962	-2.836678
43.	1.	0.	4.448151	0.476999	-3.153239
44.	1.	0.	3.248937	1.544544	-3.928838
45.	1.	0.	1.574934	2.911845	-0.702211
46.	1.	0.	2.637923	3.338074	0.620221
47.	1.	0.	0.433329	4.046254	1.141200
48.	1.	0.	1.097456	1.664736	2.946330
49.	1.	0.	0.097906	3.057036	3.369923
50.	1.	0.	1.821356	3.277321	3.039481
51.	1.	0.	-1.499465	2.748086	1.482662
52.	1.	0.	-0.444268	1.921014	-1.246991
53.	1.	0.	-2.716307	1.699178	-1.872917
54.	1.	0.	-1.591969	4.004001	-2.094074
55.	1.	0.	-1.835895	4.540629	-0.435989
56.	1.	0.	-0.190125	4.389505	-1.092240
57.	1.	0.	-5.395031	0.599908	-1.151303
58.	1.	0.	-2.604755	-3.185445	1.377558
59.	1.	0.	-3.687461	-1.880855	1.844924
60.	1.	0.	-1.503715	-2.046146	-1.388675
61.	1.	0.	-2.291969	-3.620738	-1.133551
62.	1.	0.	-2.958525	-2.467778	-2.305850
63.	1.	0.	-0.828915	-0.370346	3.402384
64.	1.	0.	-2.599986	-0.354974	3.306014
65.	1.	0.	-1.746032	-1.871787	3.629739
66.	1.	0.	-0.165217	-2.257068	0.234891
67.	1.	0.	0.561271	-1.346059	1.562091
68.	1.	0.	-0.244237	-2.889004	1.883676
69.	1.	0.	-3.733405	2.474541	0.599322

**Isomer 2.-4**	Standard Orientation

		(Ångstroms)			
Center number	Atom number	Type	X	Y	Z
1.	6.	0.	4.152738	-2.795123	-0.472385
2.	6.	0.	5.225439	-2.939353	0.415123
3.	6.	0.	5.794316	-1.803633	0.983773
4.	6.	0.	5.308339	-0.529899	0.677771
5.	6.	0.	4.230452	-0.385755	-0.200483
6.	6.	0.	3.655939	-1.524843	-0.775227
7.	6.	0.	3.643908	0.989219	-0.493266
8.	8.	0.	3.629508	-3.935893	-1.014689
9.	6.	0.	2.458774	1.307456	0.431087
10.	8.	0.	3.157038	1.096679	-1.830332
11.	6.	0.	4.178554	1.182697	-2.809740
12.	6.	0.	1.945503	2.743201	0.247654
13.	6.	0.	0.692601	3.154493	1.055021
14.	6.	0.	0.909774	3.066968	2.574480
15.	6.	0.	-0.620303	2.463748	0.640348
16.	6.	0.	-0.962730	2.455380	-0.854418
17.	6.	0.	-2.334789	1.772268	-1.045510
18.	6.	0.	-0.957598	3.853679	-1.483357
19.	6.	0.	-3.467843	-0.466750	-0.517322
20.	6.	0.	-4.697151	-0.356921	-1.055470
21.	6.	0.	-1.575000	0.250050	0.795488
22.	6.	0.	-1.625144	-0.969764	1.719440
23.	6.	0.	-2.831644	-1.902198	1.382230
24.	6.	0.	-3.245508	-1.900883	-0.094167
25.	6.	0.	-5.396096	-1.645980	-0.901776
26.	8.	0.	-6.518237	-1.965894	-1.208626
27.	8.	0.	-0.590791	1.104785	1.159058
28.	6.	0.	-2.276274	-2.635866	-1.031673
29.	6.	0.	-1.809731	-0.469205	3.174142
30.	6.	0.	-2.426526	0.500474	-0.238571
31.	6.	0.	-0.279543	-1.733283	1.647241
32.	8.	0.	-4.535233	-2.524620	-0.255325
33.	8.	0.	-3.436870	2.652446	-0.768320
34.	1.	0.	5.596822	-3.934330	0.635656
35.	1.	0.	6.632468	-1.912228	1.666145
36.	1.	0.	5.767982	0.349850	1.120321
37.	1.	0.	2.829769	-1.405964	-1.472767
38.	1.	0.	4.432467	1.745202	-0.334273
39.	1.	0.	2.911293	-3.697729	-1.615584
40.	1.	0.	1.666275	0.582565	0.224763

41.	1.	0.	2.778367	1.135894	1.464800
42.	1.	0.	4.820790	2.060585	-2.643195
43.	1.	0.	4.811810	0.285762	-2.824972
44.	1.	0.	3.683701	1.286037	-3.778285
45.	1.	0.	1.765112	2.905114	-0.818895
46.	1.	0.	2.750776	3.438558	0.523723
47.	1.	0.	0.521840	4.214304	0.822129
48.	1.	0.	1.059933	2.036675	2.905044
49.	1.	0.	0.047735	3.467438	3.119196
50.	1.	0.	1.788937	3.649554	2.868330
51.	1.	0.	-1.441391	2.972096	1.174032
52.	1.	0.	-0.220788	1.827774	-1.366339
53.	1.	0.	-2.448542	1.531090	-2.108779
54.	1.	0.	-1.286009	3.796542	-2.525788
55.	1.	0.	-1.650933	4.520280	-0.964083
56.	1.	0.	0.038851	4.301708	-1.473243
57.	1.	0.	-5.177324	0.527420	-1.449658
58.	1.	0.	-2.607958	-2.920633	1.717784
59.	1.	0.	-3.709042	-1.568407	1.946096
60.	1.	0.	-2.123905	-3.662180	-0.685492
61.	1.	0.	-2.701140	-2.669149	-2.038055
62.	1.	0.	-1.309149	-2.130994	-1.084489
63.	1.	0.	-0.960521	0.136909	3.496274
64.	1.	0.	-2.719832	0.131635	3.271774
65.	1.	0.	-1.899373	-1.326362	3.849569
66.	1.	0.	0.552790	-1.073114	1.900826
67.	1.	0.	-0.287194	-2.561059	2.363436
68.	1.	0.	-0.088489	-2.150622	0.656115
69.	1.	0.	-3.612192	2.608692	0.181155

**Isomer 2.-5**		Standard Orientation (Ångstroms)			
Center number	Atom number	Type	X	Y	Z
1.	6.	0.	4.002667	-2.809339	-0.428582
2.	6.	0.	5.132333	-2.965732	0.382309
3.	6.	0.	5.781836	-1.835193	0.877350
4.	6.	0.	5.315762	-0.557413	0.570356
5.	6.	0.	4.180232	-0.401264	-0.234554
6.	6.	0.	3.528494	-1.530755	-0.734357
7.	6.	0.	3.617686	0.984990	-0.523538
8.	8.	0.	3.318328	-3.871627	-0.951443
9.	6.	0.	2.472800	1.334420	0.439063

10.	8.	0.	3.083444	1.090000	-1.841535
11.	6.	0.	4.064840	1.106337	-2.863945
12.	6.	0.	1.965787	2.772175	0.253894
13.	6.	0.	0.712489	3.183328	1.060909
14.	6.	0.	0.929960	3.098816	2.580454
15.	6.	0.	-0.598615	2.487539	0.646889
16.	6.	0.	-0.937677	2.472534	-0.848663
17.	6.	0.	-2.306031	1.782512	-1.040587
18.	6.	0.	-0.938204	3.869195	-1.481300
19.	6.	0.	-3.423903	-0.465137	-0.515690
20.	6.	0.	-4.652921	-0.364249	-1.056383
21.	6.	0.	-1.541671	0.267406	0.803514
22.	6.	0.	-1.583599	-0.953644	1.726197
23.	6.	0.	-2.782183	-1.894956	1.385727
24.	6.	0.	-3.191724	-1.897393	-0.091911
25.	6.	0.	-5.343041	-1.657935	-0.902936
26.	8.	0.	-6.463048	-1.985335	-1.210817
27.	8.	0.	-0.566845	1.130950	1.171780
28.	6.	0.	-2.215248	-2.626063	-1.026442
29.	6.	0.	-1.775397	-0.455967	3.180944
30.	6.	0.	-2.391317	0.510450	-0.233734
31.	6.	0.	-0.232008	-1.706968	1.655759
32.	8.	0.	-4.477585	-2.530065	-0.254964
33.	8.	0.	-3.412720	2.657580	-0.763458
34.	1.	0.	5.501888	-3.961405	0.618229
35.	1.	0.	6.663134	-1.956769	1.500529
36.	1.	0.	5.833238	0.317922	0.953622
37.	1.	0.	2.659703	-1.424238	-1.374918
38.	1.	0.	4.429041	1.724039	-0.402252
39.	1.	0.	3.747993	-4.691565	-0.674629
40.	1.	0.	1.666110	0.616133	0.270774
41.	1.	0.	2.826333	1.173647	1.463609
42.	1.	0.	4.761149	1.949561	-2.738842
43.	1.	0.	4.645896	0.175208	-2.892449
44.	1.	0.	3.534757	1.223551	-3.811939
45.	1.	0.	1.783278	2.932271	-0.812438
46.	1.	0.	2.772175	3.466940	0.528066
47.	1.	0.	0.538857	4.242238	0.826039
48.	1.	0.	1.084042	2.069447	2.912134
49.	1.	0.	0.066441	3.496643	3.124827
50.	1.	0.	1.807028	3.685111	2.873359
51.	1.	0.	-1.421907	2.996241	1.176889
52.	1.	0.	-0.191803	1.847110	-1.357320

53.	1.	0.	-2.417749	1.540539	-2.103842
54.	1.	0.	-1.262412	3.807204	-2.524790
55.	1.	0.	-1.637263	4.533155	-0.966265
56.	1.	0.	0.055637	4.322987	-1.468988
57.	1.	0.	-5.138518	0.516548	-1.451897
58.	1.	0.	-2.551535	-2.911624	1.721949
59.	1.	0.	-3.663630	-1.567659	1.947224
60.	1.	0.	-2.057660	-3.651491	-0.680241
61.	1.	0.	-2.636766	-2.661893	-2.034109
62.	1.	0.	-1.250201	-2.117564	-1.077308
63.	1.	0.	-0.931633	0.156413	3.505622
64.	1.	0.	-2.690326	0.137797	3.277240
65.	1.	0.	-1.860004	-1.314467	3.855399
66.	1.	0.	0.594220	-1.041217	1.914895
67.	1.	0.	-0.236787	-2.536078	2.370689
68.	1.	0.	-0.032192	-2.120792	0.665075
69.	1.	0.	-3.591204	2.607869	0.185098

Figure. S1.3.2.3. DP4+ evaluation of theoretical and experimental data for **isomer 1** and **isomer 2**.

Functional		Solvent?		Basis Set		Type of Data	
mPW1PW91		PCM		6-311+G(d, p)		Unscaled Shifts	
		DP4+	0.00%	100.00%	–	–	–
Nuclei	sp2?	Experimental	Isomer 1	Isomer 2	Isomer 3	Isomer 4	Isomer 5
C	x	176.3	175.5995	175.3888			
C	x	103.1	106.7209	108.5368			
C	x	174.2	175.5314	175.7959			
C		84.9	84.16746	87.6209			
C		47.1	47.79313	47.91549			
C		37.5	41.46375	41.47142			
C	x	167.4	170.3223	171.145			
C	x	105.7	107.508	109.9424			
C		64.5	66.53523	69.58688			
C		36.1	38.90165	36.80816			
C		79	84.31414	84.41422			
C		34.3	36.9118	36.88451			
C		31.4	28.01359	26.07582			
C		36.7	36.07276	39.45743			
C		85.7	83.30806	87.17915			
C	x	144.9	150.9936	150.9717			
C	x	114.3	119.3343	117.6025			
C	x	158.8	162.3376	162.7552			
C	x	115.6	115.3618	117.0234			
C	x	130.4	133.8512	134.4184			
C	x	119.2	118.4148	122.4964			
C		13	17.1306	17.11839			
C		11.8	11.52774	11.40857			
C		32	30.77932	31.37726			
C		26.8	26.43702	25.48299			
C		28	26.67178	27.02591			
C		56.8	56.71631	56.17929			
H	x	5.57	5.831812	5.698948			
H		2.16	1.894551	1.916255			
H		1.71	1.550657	1.62769			
H		4.26	3.72456	4.253288			
H		1.75	1.540721	1.704199			
H		3.87	3.364486	4.011438			
H		1.79	1.453361	1.574914			
H		1.54	1.429407	2.05342			
H		1.46	0.9315673	1.188284			
H		1.86	1.249603	1.771061			
H		1.73	1.973534	1.56917			
H		4.08	3.73174	3.929781			
H	x	6.74	6.841669	6.738938			
H	x	6.71	6.71701	6.571435			
H	x	7.16	7.449932	7.159334			
H	x	6.76	7.012419	7.135683			
H		0.9	1.062949	1.042637			
H		1.03	0.659159	1.030254			
H		1.15	1.139953	1.312362			
H		1.23	0.991075	1.098727			
H		1.56	1.23039	1.265009			
H		3.21	3.078615	3.183893			

Functional	Solvent?	Basis Set	Type of Data		
mPW1PW91	PCM	6-311+G(d,p)	Unscaled Shifts		

	Isomer 1	Isomer 2	Isomer 3	Isomer 4	Isomer 5	Isomer 6
sDP4+ (H data)	0.19%	99.81%	-	-	-	-
sDP4+ (C data)	6.40%	93.60%	-	-	-	-
sDP4+ (all data)	0.01%	99.99%	-	-	-	-
uDP4+ (H data)	0.02%	99.98%	-	-	-	-
uDP4+ (C data)	0.00%	100.00%	-	-	-	-
uDP4+ (all data)	0.00%	100.00%	-	-	-	-
DP4+ (H data)	0.00%	100.00%	-	-	-	-
DP4+ (C data)	0.00%	100.00%	-	-	-	-
DP4+ (all data)	0.00%	100.00%	-	-	-	-

Table S1.3.2.4.a. Gibbs free energies[a] and equilibrium populations[b] of low-energy conformers of **isomer 2'**.

Conformers	In MeOH	
	G	*P* (%)
**isomer 2'.-1**	-942151.8222324	40.78
**isomer 2'.-2**	-942151.5900537	27.55
**isomer 2'.-3**	-942151.22923545	14.97
**isomer 2'.-4**	-942151.11753867	12.40
**isomer 2'.-5**	-942150.49128369	4.30

[a]B3LYP/6-31G (d,p), in kcal/mol. [b]From G values at 298.15K.

Table S1.3.2.4.b. Cartesian coordinates for the low-energy reoptimized MMFF conformers of **isomer 2'** at B3LYP/6-31G(d,p) level of theory in CH_3OH.

**Isomer 2'.-1**		Standard Orientation (Ångstroms)			
Center number	Atomic number	Atomic Type	X	Y	Z
1.	6.	0.	4.832006	-2.359336	-0.040218
2.	6.	0.	4.017703	-3.084766	-0.913812
3.	6.	0.	3.107019	-2.404577	-1.725184
4.	6.	0.	2.997812	-1.017493	-1.665790
5.	6.	0.	3.811773	-0.288102	-0.785091
6.	6.	0.	4.730796	-0.964085	0.017827
7.	6.	0.	3.667440	1.226862	-0.695742
8.	8.	0.	5.752069	-2.951865	0.779183
9.	6.	0.	2.399770	1.661058	0.058067
10.	8.	0.	3.568788	1.828578	-1.984566
11.	6.	0.	4.767325	1.779233	-2.738252
12.	6.	0.	2.401105	1.260612	1.539355
13.	6.	0.	1.169673	1.678548	2.378440
14.	6.	0.	0.961082	3.201295	2.410321
15.	6.	0.	-0.143728	0.946374	2.046590

16.	6.	0.	-0.100281	-0.585496	2.001515
17.	6.	0.	-1.529039	-1.109456	1.735310
18.	6.	0.	0.484534	-1.215034	3.271063
19.	6.	0.	-3.384268	-0.854019	-0.014391
20.	6.	0.	-4.361601	-1.678173	0.408157
21.	6.	0.	-1.724406	0.881456	0.223795
22.	6.	0.	-2.354636	1.772048	-0.850327
23.	6.	0.	-3.676326	1.155715	-1.407962
24.	6.	0.	-3.715739	-0.376984	-1.409687
25.	6.	0.	-5.437286	-1.690064	-0.600185
26.	8.	0.	-6.500484	-2.260728	-0.599135
27.	8.	0.	-0.643417	1.471872	0.786014
28.	6.	0.	-2.830399	-1.041422	-2.473958
29.	6.	0.	-1.326640	2.031338	-1.979571
30.	6.	0.	-2.192305	-0.334559	0.623925
31.	6.	0.	-2.706904	3.136725	-0.206810
32.	8.	0.	-5.061048	-0.844598	-1.636395
33.	8.	0.	-2.340577	-1.133039	2.921418
34.	1.	0.	4.099456	-4.168377	-0.964450
35.	1.	0.	2.481606	-2.969370	-2.410731
36.	1.	0.	2.303693	-0.488213	-2.310317
37.	1.	0.	5.384932	-0.424804	0.696769
38.	1.	0.	4.551350	1.630871	-0.171581
39.	1.	0.	5.733095	-3.907486	0.637425
40.	1.	0.	2.326742	2.748800	-0.046595
41.	1.	0.	1.532971	1.236621	-0.455545
42.	1.	0.	4.572881	2.302764	-3.677227
43.	1.	0.	5.075637	0.749021	-2.961124
44.	1.	0.	5.593585	2.284375	-2.214554
45.	1.	0.	3.282166	1.706340	2.022029
46.	1.	0.	2.540289	0.177195	1.619356
47.	1.	0.	1.393430	1.370037	3.408487
48.	1.	0.	0.689652	3.594919	1.428361
49.	1.	0.	0.162603	3.472966	3.109400
50.	1.	0.	1.875848	3.705992	2.737560
51.	1.	0.	-0.883049	1.239639	2.811538
52.	1.	0.	0.510485	-0.880628	1.137740
53.	1.	0.	-1.455350	-2.163281	1.442432
54.	1.	0.	1.539781	-0.963798	3.402468
55.	1.	0.	-0.068259	-0.895535	4.158254
56.	1.	0.	0.406792	-2.305373	3.218273
57.	1.	0.	-4.443027	-2.185480	1.358981
58.	1.	0.	-4.513710	1.481593	-0.781888

59.	1.	0.	-3.862616	1.546466	-2.414207
60.	1.	0.	-2.998120	-2.121297	-2.461048
61.	1.	0.	-3.087955	-0.662690	-3.467170
62.	1.	0.	-1.770359	-0.857108	-2.287874
63.	1.	0.	-0.415634	2.480059	-1.577391
64.	1.	0.	-1.046764	1.117607	-2.508244
65.	1.	0.	-1.752748	2.725129	-2.711419
66.	1.	0.	-1.810756	3.655200	0.140063
67.	1.	0.	-3.383158	3.008473	0.644676
68.	1.	0.	-3.210141	3.771553	-0.943498
69.	1.	0.	-2.744869	-0.260117	3.014583

**Isomer 2'.-2**		Standard Orientation (Ångstroms)			
Center number	Atom number	Type	X	Y	Z
1.	6.	0.	4.856647	-2.358867	-0.061765
2.	6.	0.	4.029528	-3.087409	-0.921563
3.	6.	0.	3.104589	-2.408886	-1.712479
4.	6.	0.	2.989103	-1.019371	-1.650617
5.	6.	0.	3.812468	-0.287311	-0.786009
6.	6.	0.	4.749822	-0.964643	-0.000056
7.	6.	0.	3.666000	1.227119	-0.692051
8.	8.	0.	5.757084	-3.059930	0.690772
9.	6.	0.	2.399475	1.658748	0.064818
10.	8.	0.	3.564743	1.833477	-1.978019
11.	6.	0.	4.757597	1.778014	-2.740023
12.	6.	0.	2.398918	1.248603	1.543346
13.	6.	0.	1.167393	1.663837	2.383790
14.	6.	0.	0.960110	3.186600	2.422056
15.	6.	0.	-0.146362	0.934084	2.047692
16.	6.	0.	-0.105195	-0.597714	1.998577
17.	6.	0.	-1.535184	-1.117926	1.731442
18.	6.	0.	0.478330	-1.232171	3.266247
19.	6.	0.	-3.391234	-0.853244	-0.016186
20.	6.	0.	-4.371084	-1.674407	0.406118
21.	6.	0.	-1.726168	0.876876	0.224427
22.	6.	0.	-2.354006	1.771321	-0.847825
23.	6.	0.	-3.678088	1.160222	-1.405528
24.	6.	0.	-3.722537	-0.372364	-1.410223
25.	6.	0.	-5.448218	-1.680224	-0.600927
26.	8.	0.	-6.513789	-2.246298	-0.599253
27.	8.	0.	-0.643287	1.463525	0.788086

28.	6.	0.	-2.840836	-1.037791	-2.476853
29.	6.	0.	-1.326004	2.028695	-1.977498
30.	6.	0.	-2.196935	-0.338687	0.622119
31.	6.	0.	-2.701382	3.136227	-0.202192
32.	8.	0.	-5.069821	-0.834626	-1.636161
33.	8.	0.	-2.346295	-1.141946	2.917819
34.	1.	0.	4.130495	-4.166646	-0.961020
35.	1.	0.	2.468365	-2.973662	-2.388067
36.	1.	0.	2.280998	-0.493349	-2.282567
37.	1.	0.	5.406321	-0.402921	0.663558
38.	1.	0.	4.550592	1.631388	-0.167919
39.	1.	0.	6.269271	-2.442149	1.228817
40.	1.	0.	2.327930	2.747202	-0.032846
41.	1.	0.	1.532226	1.238686	-0.451353
42.	1.	0.	4.560485	2.306360	-3.675663
43.	1.	0.	5.056510	0.746393	-2.968684
44.	1.	0.	5.591114	2.275763	-2.220382
45.	1.	0.	3.280438	1.689815	2.029886
46.	1.	0.	2.534728	0.164118	1.615910
47.	1.	0.	1.390135	1.350657	3.412641
48.	1.	0.	0.687712	3.584335	1.442054
49.	1.	0.	0.162524	3.455745	3.123065
50.	1.	0.	1.875425	3.689655	2.750456
51.	1.	0.	-0.886064	1.226163	2.812729
52.	1.	0.	0.504647	-0.891885	1.133822
53.	1.	0.	-1.463884	-2.171184	1.436184
54.	1.	0.	1.533704	-0.981934	3.399272
55.	1.	0.	-0.074763	-0.915394	4.154221
56.	1.	0.	0.400424	-2.322211	3.209358
57.	1.	0.	-4.453253	-2.183392	1.355991
58.	1.	0.	-4.513914	1.487603	-0.778165
59.	1.	0.	-3.863896	1.553508	-2.410872
60.	1.	0.	-3.012471	-2.117053	-2.466114
61.	1.	0.	-3.098054	-0.655938	-3.468954
62.	1.	0.	-1.779884	-0.857937	-2.291586
63.	1.	0.	-0.413329	2.474294	-1.575558
64.	1.	0.	-1.049462	1.114662	-2.507393
65.	1.	0.	-1.750490	2.724584	-2.708284
66.	1.	0.	-1.803301	3.651665	0.144257
67.	1.	0.	-3.377047	3.008943	0.649902
68.	1.	0.	-3.203570	3.773489	-0.937487
69.	1.	0.	-2.750233	-0.268966	3.011826

**Isomer 2'.-3**		Standard Orientation (Ångstroms)			
Center number	Atom number	Type	X	Y	Z
1.	6.	0.	1.910455	-2.818877	0.739526
2.	6.	0.	2.410027	-2.947725	2.041289
3.	6.	0.	3.458272	-2.127069	2.454956
4.	6.	0.	4.004160	-1.179574	1.587195
5.	6.	0.	3.496872	-1.042476	0.289360
6.	6.	0.	2.455722	-1.874913	-0.132265
7.	6.	0.	4.023501	0.024721	-0.663749
8.	8.	0.	0.882540	-3.587461	0.263360
9.	6.	0.	3.013143	1.165854	-0.864348
10.	8.	0.	4.274711	-0.497644	-1.965964
11.	6.	0.	5.360156	-1.405701	-2.025731
12.	6.	0.	2.806195	2.029456	0.389051
13.	6.	0.	1.609369	3.010630	0.355474
14.	6.	0.	1.623902	3.922054	-0.881783
15.	6.	0.	0.222670	2.371522	0.562684
16.	6.	0.	0.045293	1.472547	1.792417
17.	6.	0.	-1.429056	1.020592	1.860004
18.	6.	0.	0.474843	2.139025	3.104246
19.	6.	0.	-3.129144	-0.225010	0.403957
20.	6.	0.	-4.235854	-0.292912	1.168294
21.	6.	0.	-1.249393	0.877985	-0.635773
22.	6.	0.	-1.683090	0.498273	-2.054353
23.	6.	0.	-3.053475	-0.249697	-2.055943
24.	6.	0.	-3.318332	-1.103834	-0.810603
25.	6.	0.	-5.252144	-1.088884	0.456023
26.	8.	0.	-6.386630	-1.360661	0.765938
27.	8.	0.	-0.128898	1.639168	-0.644500
28.	6.	0.	-2.495944	-2.398222	-0.726483
29.	6.	0.	-0.576383	-0.348807	-2.730162
30.	6.	0.	-1.910374	0.550172	0.510408
31.	6.	0.	-1.856570	1.798906	-2.877563
32.	8.	0.	-4.707952	-1.490623	-0.756414
33.	8.	0.	-2.293853	2.028477	2.410682
34.	1.	0.	1.986050	-3.685420	2.719128
35.	1.	0.	3.852382	-2.232348	3.461552
36.	1.	0.	4.823621	-0.547366	1.917418
37.	1.	0.	2.068865	-1.801216	-1.143008
38.	1.	0.	4.960705	0.434717	-0.247508
39.	1.	0.	0.587986	-4.193056	0.956319

40.	1.	0.	3.379552	1.777565	-1.695441
41.	1.	0.	2.065291	0.727304	-1.184718
42.	1.	0.	5.474740	-1.693554	-3.073319
43.	1.	0.	5.180820	-2.307822	-1.425570
44.	1.	0.	6.295820	-0.938404	-1.681391
45.	1.	0.	3.714398	2.623685	0.559010
46.	1.	0.	2.711052	1.379848	1.265799
47.	1.	0.	1.728227	3.662977	1.230658
48.	1.	0.	1.464102	3.356769	-1.802744
49.	1.	0.	0.838750	4.683273	-0.817402
50.	1.	0.	2.584543	4.440694	-0.965190
51.	1.	0.	-0.506033	3.196291	0.640119
52.	1.	0.	0.643342	0.565150	1.634854
53.	1.	0.	-1.497373	0.190766	2.573243
54.	1.	0.	1.548509	2.341711	3.127199
55.	1.	0.	-0.065019	3.075934	3.265124
56.	1.	0.	0.242291	1.484021	3.949705
57.	1.	0.	-4.437254	0.218179	2.099108
58.	1.	0.	-3.861811	0.488120	-2.097417
59.	1.	0.	-3.132967	-0.858102	-2.963276
60.	1.	0.	-2.848785	-2.989478	0.122722
61.	1.	0.	-2.634017	-2.988904	-1.636700
62.	1.	0.	-1.430279	-2.207287	-0.587253
63.	1.	0.	0.367544	0.200706	-2.758123
64.	1.	0.	-0.403280	-1.296756	-2.216438
65.	1.	0.	-0.865752	-0.572925	-3.761993
66.	1.	0.	-0.910596	2.335158	-2.977262
67.	1.	0.	-2.585089	2.468511	-2.408732
68.	1.	0.	-2.221959	1.553752	-3.880221
69.	1.	0.	-2.586631	2.589239	1.680168

**Isomer 2'.-4**		Standard Orientation (Ångstroms)			
Center number	Atom number	Type	X	Y	Z
1.	6.	0.	1.985806	-2.893125	0.719330
2.	6.	0.	2.440338	-2.962996	2.042474
3.	6.	0.	3.426062	-2.079090	2.469194
4.	6.	0.	3.958783	-1.124108	1.596637
5.	6.	0.	3.493937	-1.041974	0.281307
6.	6.	0.	2.510114	-1.939372	-0.154332
7.	6.	0.	3.990474	0.034279	-0.676935
8.	8.	0.	1.017639	-3.782482	0.340102

9.	6.	0.	2.961374	1.162957	-0.859020
10.	8.	0.	4.228941	-0.483642	-1.984071
11.	6.	0.	5.343800	-1.354570	-2.064436
12.	6.	0.	2.784073	2.036820	0.391653
13.	6.	0.	1.593254	3.025691	0.378770
14.	6.	0.	1.602670	3.950560	-0.848535
15.	6.	0.	0.204677	2.392051	0.590753
16.	6.	0.	0.038716	1.468154	1.803582
17.	6.	0.	-1.432262	1.005884	1.874994
18.	6.	0.	0.474210	2.113898	3.123856
19.	6.	0.	-3.120091	-0.248241	0.409946
20.	6.	0.	-4.218555	-0.362159	1.180034
21.	6.	0.	-1.275538	0.916082	-0.624071
22.	6.	0.	-1.721902	0.565400	-2.046641
23.	6.	0.	-3.069221	-0.223143	-2.049732
24.	6.	0.	-3.292634	-1.110366	-0.818970
25.	6.	0.	-5.214460	-1.179547	0.462903
26.	8.	0.	-6.337482	-1.491343	0.774342
27.	8.	0.	-0.164356	1.690037	-0.629981
28.	6.	0.	-2.421628	-2.374870	-0.765441
29.	6.	0.	-0.602964	-0.228007	-2.765271
30.	6.	0.	-1.919157	0.553356	0.521206
31.	6.	0.	-1.944865	1.884680	-2.827241
32.	8.	0.	-4.665122	-1.546816	-0.760010
33.	8.	0.	-2.301108	1.998292	2.446133
34.	1.	0.	2.020591	-3.712259	2.705030
35.	1.	0.	3.788975	-2.138464	3.491241
36.	1.	0.	4.734054	-0.444735	1.939742
37.	1.	0.	2.169228	-1.890509	-1.186278
38.	1.	0.	4.927673	0.457824	-0.275361
39.	1.	0.	0.843567	-3.676767	-0.604461
40.	1.	0.	3.297712	1.769995	-1.706188
41.	1.	0.	2.008576	0.712586	-1.147058
42.	1.	0.	5.444023	-1.645559	-3.112700
43.	1.	0.	5.210175	-2.257641	-1.453785
44.	1.	0.	6.270068	-0.852637	-1.745300
45.	1.	0.	3.698928	2.627126	0.538295
46.	1.	0.	2.703863	1.393502	1.274330
47.	1.	0.	1.724860	3.667219	1.260075
48.	1.	0.	1.431582	3.396513	-1.774297
49.	1.	0.	0.822784	4.715816	-0.769396
50.	1.	0.	2.565714	4.464286	-0.934234
51.	1.	0.	-0.518237	3.218929	0.694525

52.	1.	0.	0.640131	0.566606	1.626861
53.	1.	0.	-1.489586	0.164567	2.575147
54.	1.	0.	1.546688	2.323184	3.142514
55.	1.	0.	-0.070309	3.044363	3.305370
56.	1.	0.	0.252455	1.442173	3.958879
57.	1.	0.	-4.428944	0.124973	2.121635
58.	1.	0.	-3.899865	0.490250	-2.065933
59.	1.	0.	-3.142222	-0.814377	-2.969046
60.	1.	0.	-2.740180	-2.997779	0.073787
61.	1.	0.	-2.538694	-2.950977	-1.688134
62.	1.	0.	-1.365882	-2.134140	-0.621805
63.	1.	0.	0.327283	0.344217	-2.785091
64.	1.	0.	-0.400753	-1.187519	-2.283599
65.	1.	0.	-0.899863	-0.427714	-3.799855
66.	1.	0.	-1.016438	2.451630	-2.922335
67.	1.	0.	-2.685795	2.516930	-2.327356
68.	1.	0.	-2.317708	1.660269	-3.832038
69.	1.	0.	-2.592610	2.576290	1.728633

**Isomer 2'.-5**		Standard Orientation (Ångstroms)			
Center number	Atom number	Type	X	Y	Z
1.	6.	0.	2.019580	-2.877291	0.733807
2.	6.	0.	2.529366	-2.960155	2.036099
3.	6.	0.	3.534917	-2.082928	2.428916
4.	6.	0.	4.033174	-1.121393	1.543312
5.	6.	0.	3.514254	-1.026599	0.249219
6.	6.	0.	2.510345	-1.917437	-0.152803
7.	6.	0.	3.976451	0.055116	-0.720121
8.	8.	0.	1.034568	-3.761227	0.388372
9.	6.	0.	2.937005	1.178594	-0.870192
10.	8.	0.	4.179852	-0.458471	-2.034888
11.	6.	0.	5.295433	-1.324230	-2.149834
12.	6.	0.	2.772739	2.034848	0.394164
13.	6.	0.	1.575662	3.016398	0.411819
14.	6.	0.	1.570187	3.967423	-0.795509
15.	6.	0.	0.191628	2.371990	0.619161
16.	6.	0.	0.041314	1.416044	1.809903
17.	6.	0.	-1.435384	0.973345	1.881266
18.	6.	0.	0.498401	2.031506	3.138205
19.	6.	0.	-3.133177	-0.250890	0.408454
20.	6.	0.	-4.234666	-0.370011	1.173421

21.	6.	0.	-1.289821	0.918204	-0.614416
22.	6.	0.	-1.737260	0.581646	-2.039612
23.	6.	0.	-3.082138	-0.210998	-2.049526
24.	6.	0.	-3.302510	-1.107521	-0.825212
25.	6.	0.	-5.225872	-1.187932	0.452828
26.	8.	0.	-6.347994	-1.506766	0.762259
27.	8.	0.	-0.177627	1.689794	-0.612825
28.	6.	0.	-2.426246	-2.368615	-0.779869
29.	6.	0.	-0.618640	-0.200716	-2.770541
30.	6.	0.	-1.931250	0.544391	0.526391
31.	6.	0.	-1.966222	1.910132	-2.802745
32.	8.	0.	-4.672930	-1.550934	-0.769644
33.	8.	0.	-2.296799	2.023318	2.352788
34.	1.	0.	2.135820	-3.714690	2.708740
35.	1.	0.	3.940787	-2.152981	3.434065
36.	1.	0.	4.824250	-0.446889	1.858991
37.	1.	0.	2.127236	-1.859120	-1.169277
38.	1.	0.	4.922783	0.481935	-0.344156
39.	1.	0.	0.813494	-3.640998	-0.544592
40.	1.	0.	3.254348	1.798780	-1.715108
41.	1.	0.	1.982574	0.725668	-1.148043
42.	1.	0.	5.366479	-1.612099	-3.201342
43.	1.	0.	5.183508	-2.229642	-1.538187
44.	1.	0.	6.228624	-0.819376	-1.856338
45.	1.	0.	3.686656	2.627704	0.537108
46.	1.	0.	2.708205	1.377563	1.268176
47.	1.	0.	1.711998	3.641097	1.304875
48.	1.	0.	1.394541	3.431484	-1.731003
49.	1.	0.	0.785483	4.724811	-0.694114
50.	1.	0.	2.529436	4.488955	-0.878225
51.	1.	0.	-0.544401	3.178824	0.753066
52.	1.	0.	0.634982	0.514208	1.611224
53.	1.	0.	-1.507208	0.113451	2.564213
54.	1.	0.	1.576648	2.205223	3.159702
55.	1.	0.	0.000331	2.988568	3.325796
56.	1.	0.	0.264486	1.358011	3.970839
57.	1.	0.	-4.454932	0.121924	2.109681
58.	1.	0.	-3.914611	0.500036	-2.059829
59.	1.	0.	-3.152979	-0.796030	-2.973099
60.	1.	0.	-2.741315	-2.998033	0.055948
61.	1.	0.	-2.540142	-2.940035	-1.705868
62.	1.	0.	-1.371929	-2.121335	-0.635495
63.	1.	0.	0.311266	0.372238	-2.784476

64.	1.	0.	-0.414954	-1.166975	-2.302624
65.	1.	0.	-0.916810	-0.386950	-3.807306
66.	1.	0.	-1.038612	2.478822	-2.896020
67.	1.	0.	-2.703685	2.534953	-2.289379
68.	1.	0.	-2.344070	1.697004	-3.808269
69.	1.	0.	-2.194456	2.080653	3.312324

Table S1.3.2.5.a. Gibbs free energies[a] and equilibrium populations[b] of low-energy conformers of **isomer 1'**.

Conformers	In MeOH	
	G	*P* (%)
**isomer 1'.-1**	-942153.6733869	59.17
**isomer 1'.-2**	-942153.45250338	30.74
**isomer 1'.-3**	-942151.63285001	5.06
**isomer 1'.-4**	-942151.28244849	4.02
**isomer 1'.-5**	-942151.00014424	1.01

[a]B3LYP/6-31G (d,p), in kcal/mol. [b]From G values at 298.15K.

Table S1.3.2.5.b. Cartesian coordinates for the low-energy reoptimized MMFF conformers of **isomer 1'** at B3LYP/6-31G (d,p) level of theory in CH_3OH.

**Isomer 1'.-1**		Standard Orientation (Ångstroms)			
Center number	Atomic number	Atomic Type	X	Y	Z
1.	6.	0.	4.307648	2.612827	-1.081699
2.	6.	0.	5.152561	3.057343	-0.061393
3.	6.	0.	5.770899	2.122896	0.772137
4.	6.	0.	5.547834	0.758891	0.602235
5.	6.	0.	4.696193	0.311532	-0.419292
6.	6.	0.	4.086715	1.242126	-1.261538
7.	6.	0.	4.422074	-1.178567	-0.583769
8.	8.	0.	3.671879	3.464585	-1.941584
9.	6.	0.	3.454352	-1.730732	0.476527
10.	8.	0.	5.609334	-1.957048	-0.458007
11.	6.	0.	6.521055	-1.797825	-1.530639
12.	6.	0.	2.031716	-1.167411	0.372924
13.	6.	0.	1.099204	-1.608912	1.519996
14.	6.	0.	0.921595	-3.135095	1.590609
15.	6.	0.	-0.270978	-0.911568	1.466327
16.	6.	0.	-0.296832	0.621173	1.499878
17.	6.	0.	-1.773109	1.074372	1.532363
18.	6.	0.	0.497941	1.223437	2.663107
19.	6.	0.	-3.891026	0.805813	0.109333
20.	6.	0.	-4.462438	2.026003	0.144979

21.	6.	0.	-2.109887	-0.812387	-0.083774
22.	6.	0.	-2.751599	-1.538335	-1.267639
23.	6.	0.	-4.071388	-0.840373	-1.718972
24.	6.	0.	-4.837883	-0.148757	-0.586064
25.	6.	0.	-5.709611	1.990105	-0.637421
26.	8.	0.	-6.492836	2.870959	-0.896190
27.	8.	0.	-0.931241	-1.374893	0.257364
28.	6.	0.	-5.525836	-1.101688	0.402655
29.	6.	0.	-2.989735	-3.025763	-0.908614
30.	6.	0.	-2.597466	0.309784	0.524079
31.	6.	0.	-1.756426	-1.489842	-2.455306
32.	8.	0.	-5.870143	0.700071	-1.126477
33.	8.	0.	-2.336451	1.002748	2.849784
34.	1.	0.	5.330176	4.121801	0.076121
35.	1.	0.	6.433631	2.470134	1.559678
36.	1.	0.	6.040841	0.032726	1.240036
37.	1.	0.	3.436741	0.923409	-2.071252
38.	1.	0.	3.982279	-1.343550	-1.583061
39.	1.	0.	3.915335	4.374036	-1.724107
40.	1.	0.	3.452748	-2.819144	0.360026
41.	1.	0.	3.873899	-1.518279	1.468257
42.	1.	0.	6.060300	-2.066663	-2.493725
43.	1.	0.	6.902956	-0.770728	-1.602645
44.	1.	0.	7.356806	-2.474736	-1.338687
45.	1.	0.	1.584935	-1.464765	-0.584398
46.	1.	0.	2.089372	-0.073757	0.358312
47.	1.	0.	1.552347	-1.285539	2.466830
48.	1.	0.	0.225951	-3.411904	2.390490
49.	1.	0.	1.870933	-3.636585	1.794282
50.	1.	0.	0.524439	-3.529408	0.650533
51.	1.	0.	-0.872190	-1.279164	2.314642
52.	1.	0.	0.122891	0.983373	0.552056
53.	1.	0.	-1.802278	2.141411	1.279137
54.	1.	0.	0.144720	0.838065	3.622989
55.	1.	0.	1.567889	1.019901	2.570609
56.	1.	0.	0.366993	2.309813	2.684060
57.	1.	0.	-4.086911	2.940682	0.581852
58.	1.	0.	-3.833752	-0.060806	-2.450237
59.	1.	0.	-4.712225	-1.568380	-2.227739
60.	1.	0.	-6.196337	-1.779648	-0.133129
61.	1.	0.	-4.800794	-1.693742	0.965330
62.	1.	0.	-6.118057	-0.518896	1.112556
63.	1.	0.	-3.365551	-3.559881	-1.787081

64.	1.	0.	-2.056232	-3.498674	-0.595395
65.	1.	0.	-3.718420	-3.151506	-0.104822
66.	1.	0.	-0.834020	-2.025710	-2.221120
67.	1.	0.	-2.212454	-1.956004	-3.334996
68.	1.	0.	-1.501067	-0.457396	-2.713587
69.	1.	0.	-2.723669	0.123883	2.952897

**Isomer 1'.-2**		Standard Orientation (Ångstroms)			
Center number	Atom number	Type	X	Y	Z
1.	6.	0.	5.686165	2.175162	-0.151730
2.	6.	0.	5.013621	2.863708	-1.169246
3.	6.	0.	4.129416	2.170203	-1.989023
4.	6.	0.	3.907202	0.801725	-1.806299
5.	6.	0.	4.567514	0.115763	-0.782833
6.	6.	0.	5.462213	0.810310	0.040697
7.	6.	0.	4.310049	-1.364412	-0.528097
8.	8.	0.	6.552927	2.893429	0.623672
9.	6.	0.	3.367330	-1.615112	0.661406
10.	8.	0.	5.510529	-2.069255	-0.218796
11.	6.	0.	6.387266	-2.225368	-1.321238
12.	6.	0.	1.939368	-1.098646	0.447036
13.	6.	0.	1.034259	-1.235236	1.688655
14.	6.	0.	0.833125	-2.697779	2.119760
15.	6.	0.	-0.326422	-0.540903	1.510069
16.	6.	0.	-0.328487	0.960161	1.191174
17.	6.	0.	-1.800601	1.424639	1.185767
18.	6.	0.	0.521710	1.790597	2.159221
19.	6.	0.	-3.971317	0.888529	-0.060615
20.	6.	0.	-4.532888	2.092645	-0.288282
21.	6.	0.	-2.214349	-0.760656	0.039792
22.	6.	0.	-2.915390	-1.725214	-0.917858
23.	6.	0.	-4.256945	-1.130560	-1.446211
24.	6.	0.	-4.958338	-0.185024	-0.463608
25.	6.	0.	-5.820869	1.896255	-0.973700
26.	8.	0.	-6.611556	2.706547	-1.393261
27.	8.	0.	-1.023329	-1.249822	0.450205
28.	6.	0.	-5.589031	-0.871182	0.756267
29.	6.	0.	-3.139891	-3.088590	-0.218389
30.	6.	0.	-2.663911	0.476395	0.395164
31.	6.	0.	-1.982796	-1.961013	-2.132738
32.	8.	0.	-6.015707	0.531384	-1.133548

33.	8.	0.	-2.344731	1.503168	2.511558
34.	1.	0.	5.203493	3.923310	-1.302758
35.	1.	0.	3.614433	2.699083	-2.785898
36.	1.	0.	3.224536	0.268284	-2.461929
37.	1.	0.	5.996245	0.268213	0.818267
38.	1.	0.	3.855796	-1.798361	-1.435640
39.	1.	0.	6.955772	2.303302	1.274273
40.	1.	0.	3.369053	-2.695171	0.839211
41.	1.	0.	3.807981	-1.147213	1.551484
42.	1.	0.	5.904852	-2.775991	-2.143291
43.	1.	0.	6.741689	-1.261264	-1.709819
44.	1.	0.	7.244114	-2.802361	-0.965662
45.	1.	0.	1.474486	-1.630870	-0.392647
46.	1.	0.	1.989722	-0.045211	0.151299
47.	1.	0.	1.523112	-0.711558	2.521881
48.	1.	0.	0.164098	-2.761867	2.984572
49.	1.	0.	1.780341	-3.164292	2.402365
50.	1.	0.	0.391680	-3.288053	1.311275
51.	1.	0.	-0.917049	-0.680329	2.427488
52.	1.	0.	0.054932	1.098585	0.171743
53.	1.	0.	-1.848278	2.417154	0.711195
54.	1.	0.	0.243974	1.596932	3.200441
55.	1.	0.	1.587503	1.576780	2.049170
56.	1.	0.	0.387469	2.861152	1.965641
57.	1.	0.	-4.129130	3.078960	-0.106600
58.	1.	0.	-4.061621	-0.544424	-2.350483
59.	1.	0.	-4.927098	-1.946904	-1.736397
60.	1.	0.	-6.281108	-1.653807	0.432238
61.	1.	0.	-4.830426	-1.309705	1.407250
62.	1.	0.	-6.146709	-0.131806	1.336035
63.	1.	0.	-3.560858	-3.802576	-0.933617
64.	1.	0.	-2.193746	-3.491143	0.150283
65.	1.	0.	-3.828081	-3.015181	0.626301
66.	1.	0.	-1.050405	-2.441725	-1.827836
67.	1.	0.	-2.482919	-2.609007	-2.860184
68.	1.	0.	-1.739110	-1.017394	-2.630985
69.	1.	0.	-2.011823	2.312463	2.921853

**Isomer 1'.-3**		Standard Orientation (Ångstroms)			
Center number	Atom number	Type	X	Y	Z
1.	6.	0.	1.689935	2.658312	-0.381371

2.	6.	0.	1.977992	3.078437	0.922888
3.	6.	0.	2.838252	2.314471	1.713764
4.	6.	0.	3.403300	1.135933	1.228695
5.	6.	0.	3.112204	0.710309	-0.076049
6.	6.	0.	2.271447	1.483754	-0.878117
7.	6.	0.	3.672910	-0.609274	-0.592472
8.	8.	0.	0.855658	3.344318	-1.211767
9.	6.	0.	2.975602	-1.837735	0.018588
10.	8.	0.	5.054959	-0.770392	-0.281800
11.	6.	0.	5.909020	0.121848	-0.975622
12.	6.	0.	1.473717	-1.923048	-0.284251
13.	6.	0.	0.815567	-3.211911	0.264870
14.	6.	0.	1.107853	-4.405287	-0.662207
15.	6.	0.	-0.714452	-3.119115	0.528957
16.	6.	0.	-1.158784	-2.544914	1.885038
17.	6.	0.	-1.000223	-1.009038	1.905161
18.	6.	0.	-2.593329	-2.980065	2.225294
19.	6.	0.	-1.890280	1.003116	0.582191
20.	6.	0.	-1.370702	2.054412	1.254147
21.	6.	0.	-1.734465	-1.149562	-0.492226
22.	6.	0.	-2.242895	-0.631706	-1.841214
23.	6.	0.	-2.374330	0.919941	-1.840880
24.	6.	0.	-2.804418	1.527597	-0.502154
25.	6.	0.	-1.674916	3.267243	0.494944
26.	8.	0.	-1.206507	4.387624	0.602160
27.	8.	0.	-1.384222	-2.451939	-0.581118
28.	6.	0.	-4.285806	1.352888	-0.144315
29.	6.	0.	-3.584142	-1.333751	-2.173979
30.	6.	0.	-1.612520	-0.412167	0.652234
31.	6.	0.	-1.232387	-1.005978	-2.952737
32.	8.	0.	-2.544333	2.952146	-0.522596
33.	8.	0.	-1.520775	-0.431213	3.100298
34.	1.	0.	1.517090	3.982713	1.309177
35.	1.	0.	3.065085	2.646942	2.723064
36.	1.	0.	4.078872	0.550164	1.843703
37.	1.	0.	2.043048	1.184643	-1.897543
38.	1.	0.	3.540749	-0.637507	-1.688626
39.	1.	0.	0.357146	4.009060	-0.703352
40.	1.	0.	3.503013	-2.722387	-0.355647
41.	1.	0.	3.134925	-1.812215	1.103802
42.	1.	0.	6.931828	-0.127193	-0.683065
43.	1.	0.	5.813195	0.005101	-2.066399
44.	1.	0.	5.708825	1.170570	-0.719655

45.	1.	0.	1.314008	-1.885653	-1.369096
46.	1.	0.	0.990590	-1.030468	0.116605
47.	1.	0.	1.263637	-3.429824	1.245033
48.	1.	0.	2.176675	-4.492789	-0.876079
49.	1.	0.	0.583946	-4.286055	-1.616988
50.	1.	0.	0.782117	-5.350492	-0.214818
51.	1.	0.	-1.107727	-4.139439	0.467307
52.	1.	0.	-0.492209	-2.960444	2.649568
53.	1.	0.	0.067348	-0.760786	1.947028
54.	1.	0.	-2.668163	-4.071596	2.249927
55.	1.	0.	-2.883297	-2.607657	3.211079
56.	1.	0.	-3.316497	-2.614339	1.489065
57.	1.	0.	-0.685734	2.043591	2.087929
58.	1.	0.	-1.397357	1.359337	-2.068280
59.	1.	0.	-3.058244	1.226936	-2.639795
60.	1.	0.	-4.918123	1.718968	-0.958061
61.	1.	0.	-4.529534	0.306194	0.048630
62.	1.	0.	-4.510943	1.929456	0.756593
63.	1.	0.	-3.926062	-1.028131	-3.168113
64.	1.	0.	-3.450371	-2.418530	-2.177740
65.	1.	0.	-4.373806	-1.094070	-1.459266
66.	1.	0.	-0.241444	-0.594523	-2.740405
67.	1.	0.	-1.143302	-2.088417	-3.062591
68.	1.	0.	-1.571899	-0.588664	-3.906442
69.	1.	0.	-2.480520	-0.365108	3.006957

**Isomer 1'.-4**		Standard Orientation (Ångstroms)			
Center number	Atom number	Type	X	Y	Z
1.	6.	0.	1.184230	-2.587460	-0.270237
2.	6.	0.	1.339703	-2.587613	-1.660417
3.	6.	0.	2.273331	-1.726074	-2.242611
4.	6.	0.	3.040599	-0.860848	-1.463964
5.	6.	0.	2.870707	-0.838841	-0.070192
6.	6.	0.	1.953945	-1.715060	0.513470
7.	6.	0.	3.713866	0.072743	0.813912
8.	8.	0.	0.315888	-3.404987	0.385907
9.	6.	0.	3.660725	1.579052	0.500158
10.	8.	0.	5.104115	-0.251878	0.712762
11.	6.	0.	5.449559	-1.523188	1.231172
12.	6.	0.	2.307115	2.302460	0.660683
13.	6.	0.	1.336458	2.251726	-0.543188

14.	6.	0.	1.945887	2.928579	-1.783813
15.	6.	0.	-0.013473	2.929506	-0.186971
16.	6.	0.	-1.053086	2.976489	-1.316194
17.	6.	0.	-1.414344	1.522190	-1.687972
18.	6.	0.	-2.277916	3.819153	-0.930869
19.	6.	0.	-2.261649	-0.605560	-0.536620
20.	6.	0.	-2.119147	-1.535520	-1.506313
21.	6.	0.	-1.337397	1.167580	0.802672
22.	6.	0.	-1.651690	0.479735	2.132251
23.	6.	0.	-2.098036	-0.996237	1.911246
24.	6.	0.	-2.938994	-1.246447	0.653468
25.	6.	0.	-2.380460	-2.843165	-0.907638
26.	8.	0.	-2.087616	-3.952227	-1.320179
27.	8.	0.	-0.609420	2.292665	0.984427
28.	6.	0.	-4.415543	-0.843230	0.759083
29.	6.	0.	-2.727915	1.319224	2.869116
30.	6.	0.	-1.731765	0.733493	-0.431287
31.	6.	0.	-0.392911	0.449104	3.029302
32.	8.	0.	-2.915111	-2.663430	0.346196
33.	8.	0.	-2.464188	1.444294	-2.650075
34.	1.	0.	0.738608	-3.256581	-2.269960
35.	1.	0.	2.403341	-1.739384	-3.321295
36.	1.	0.	3.776976	-0.214320	-1.930481
37.	1.	0.	1.828899	-1.748093	1.592006
38.	1.	0.	3.385844	-0.076759	1.857573
39.	1.	0.	-0.269819	-3.866607	-0.240710
40.	1.	0.	4.380113	2.037043	1.187495
41.	1.	0.	4.078766	1.733810	-0.499654
42.	1.	0.	5.170508	-1.616782	2.292548
43.	1.	0.	4.976706	-2.343689	0.675724
44.	1.	0.	6.534849	-1.614123	1.143356
45.	1.	0.	2.518099	3.360502	0.871224
46.	1.	0.	1.795479	1.919394	1.549352
47.	1.	0.	1.142253	1.201007	-0.782060
48.	1.	0.	1.343886	2.770138	-2.681966
49.	1.	0.	2.939439	2.529964	-1.997434
50.	1.	0.	2.051289	4.010311	-1.632952
51.	1.	0.	0.192085	3.947559	0.164205
52.	1.	0.	-0.596681	3.445748	-2.192000
53.	1.	0.	-0.560391	1.065431	-2.203056
54.	1.	0.	-2.802920	3.414488	-0.060691
55.	1.	0.	-1.977270	4.843636	-0.689702
56.	1.	0.	-2.980460	3.871403	-1.767216

57.	1.	0.	-1.681602	-1.420003	-2.486630
58.	1.	0.	-1.206317	-1.621668	1.803329
59.	1.	0.	-2.630081	-1.349837	2.801527
60.	1.	0.	-4.873887	-1.319675	1.630292
61.	1.	0.	-4.531628	0.238703	0.849495
62.	1.	0.	-4.946159	-1.172195	-0.137944
63.	1.	0.	-2.943537	0.873176	3.845531
64.	1.	0.	-2.362783	2.336821	3.033331
65.	1.	0.	-3.665500	1.385341	2.313594
66.	1.	0.	-0.077448	1.456103	3.308936
67.	1.	0.	-0.612485	-0.109455	3.945127
68.	1.	0.	0.438210	-0.047099	2.521739
69.	1.	0.	-3.296525	1.629436	-2.194432

**Isomer 1'.-5**		Standard Orientation (Ångstroms)			
Center number	Atom number	Type	X	Y	Z
1.	6.	0.	1.711655	2.654075	-0.383679
2.	6.	0.	2.006145	3.072019	0.920257
3.	6.	0.	2.862330	2.301536	1.709223
4.	6.	0.	3.417085	1.118158	1.223506
5.	6.	0.	3.119735	0.694688	-0.080711
6.	6.	0.	2.283233	1.474473	-0.880758
7.	6.	0.	3.670649	-0.628201	-0.599251
8.	8.	0.	0.883960	3.347001	-1.212704
9.	6.	0.	2.964845	-1.852985	0.009239
10.	8.	0.	5.051596	-0.799390	-0.288244
11.	6.	0.	5.912062	0.086328	-0.982768
12.	6.	0.	1.462831	-1.930638	-0.295016
13.	6.	0.	0.798315	-3.215551	0.256251
14.	6.	0.	1.081479	-4.410360	-0.671836
15.	6.	0.	-0.730544	-3.114623	0.524274
16.	6.	0.	-1.174122	-2.538166	1.880287
17.	6.	0.	-1.004812	-1.003902	1.899279
18.	6.	0.	-2.615025	-2.960470	2.209541
19.	6.	0.	-1.887681	1.014020	0.588210
20.	6.	0.	-1.344418	2.063792	1.241771
21.	6.	0.	-1.748958	-1.138836	-0.485736
22.	6.	0.	-2.267482	-0.620990	-1.830155
23.	6.	0.	-2.396093	0.930455	-1.829824
24.	6.	0.	-2.809436	1.541636	-0.487093
25.	6.	0.	-1.648615	3.274901	0.483014

26.	8.	0.	-1.165319	4.391580	0.577187
27.	8.	0.	-1.400846	-2.444246	-0.580090
28.	6.	0.	-4.286901	1.377165	-0.111057
29.	6.	0.	-3.614861	-1.318654	-2.146958
30.	6.	0.	-1.620371	-0.403162	0.656888
31.	6.	0.	-1.269803	-0.999138	-2.951676
32.	8.	0.	-2.538186	2.965133	-0.516798
33.	8.	0.	-1.627842	-0.408707	3.039537
34.	1.	0.	1.553544	3.980458	1.306412
35.	1.	0.	3.096422	2.634413	2.716947
36.	1.	0.	4.091943	0.528487	1.835733
37.	1.	0.	2.049397	1.176636	-1.899269
38.	1.	0.	3.538549	-0.653617	-1.695358
39.	1.	0.	0.377670	4.004826	-0.701396
40.	1.	0.	3.487765	-2.740408	-0.364766
41.	1.	0.	3.123552	-1.829411	1.094781
42.	1.	0.	5.816599	-0.031828	-2.073369
43.	1.	0.	5.718080	1.136709	-0.728873
44.	1.	0.	6.933002	-0.168554	-0.688700
45.	1.	0.	1.303887	-1.894472	-1.379965
46.	1.	0.	0.983141	-1.034885	0.103112
47.	1.	0.	1.248666	-3.436620	1.235028
48.	1.	0.	0.752376	-5.353945	-0.223531
49.	1.	0.	2.149054	-4.503301	-0.889922
50.	1.	0.	0.554382	-4.287937	-1.624376
51.	1.	0.	-1.129001	-4.133286	0.467123
52.	1.	0.	-0.509025	-2.966539	2.642360
53.	1.	0.	0.069157	-0.760003	1.922566
54.	1.	0.	-3.310848	-2.651320	1.424400
55.	1.	0.	-2.680211	-4.048790	2.308559
56.	1.	0.	-2.945440	-2.503420	3.143620
57.	1.	0.	-0.648817	2.054114	2.066147
58.	1.	0.	-1.420827	1.367258	-2.069633
59.	1.	0.	-3.088593	1.237899	-2.621195
60.	1.	0.	-4.926152	1.744660	-0.918914
61.	1.	0.	-4.532943	0.333079	0.091184
62.	1.	0.	-4.496018	1.955476	0.792121
63.	1.	0.	-3.966422	-1.013579	-3.137955
64.	1.	0.	-3.485827	-2.404041	-2.149803
65.	1.	0.	-4.394511	-1.073383	-1.423378
66.	1.	0.	-1.185279	-2.081956	-3.061858
67.	1.	0.	-1.617263	-0.581297	-3.902311
68.	1.	0.	-0.275241	-0.590867	-2.749580

69.	1.	0.	-1.089124	-0.626947	3.811611

Figure S1.3.2.6. DP4+ evaluation of theoretical and experimental data for **isomer 2'** and **isomer 1'**.

Functional		Solvent?		Basis Set		Type of Data	
mPW1PW91		PCM		6-311+G(d,p)		Unscaled Shifts	
		DP4+	100.00%	0.00%	-	-	-
Nuclei	sp2?	Experimental	Isomer 2'	Isomer 1'	Isomer 3	Isomer 4	Isomer 5
C	x	176.4	175.7305	175.4114			
C	x	104.7	106.112	106.1925			
C	x	174.5	175.6008	174.7955			
C		84.7	82.43749	83.03375			
C		47.5	48.31363	47.59012			
C		37.7	41.30493	36.86997			
C	x	167.1	171.3481	175.1431			
C	x	104.7	110.2291	110.195			
C		66.8	69.39453	66.75145			
C		35.6	36.833	37.3759			
C		79.4	84.23205	83.51801			
C		34.1	35.59547	41.46283			
C		31.4	25.78167	24.49975			
C		36.7	37.18098	39.75506			
C		85.7	87.7327	87.20642			
C	x	145	149.1559	149.9934			
C	x	114.3	118.4402	117.8847			
C	x	158.8	162.2176	162.7798			
C	x	115.6	116.1096	117.243			
C	x	130.4	134.544	134.4194			
C	x	119.1	122.6173	122.6188			
C		13.1	16.2469	17.27769			
C		12.3	11.59317	10.70276			
C		32.1	31.44547	30.55897			
C		26.4	26.8803	26.44244			
C		28.3	27.76035	27.22589			
C		56.8	56.05032	56.03812			
H	x	5.66	5.77223	5.37368			
H		2.14	1.955667	1.939274			
H		1.78	1.618301	1.619262			
H		4.05	3.576736	3.880524			
H		1.88	1.023925	1.248063			
H		3.95	3.35236	3.428665			
H		1.75	1.679183	1.247518			
H		1.56	1.146601	0.982267			
H		1.47	1.156324	0.787661			
H		1.83	2.135887	2.060618			
H		1.73	1.782307	1.094057			
H		4.07	3.998105	3.773833			
H	x	6.73	6.659736	6.798262			
H	x	6.7	6.701019	6.715927			
H	x	7.15	7.366441	7.496773			
H	x	6.75	7.153883	7.053561			
H		0.89	1.154227	1.043837			
H		0.99	0.615023	0.451832			
H		1.1	1.067737	1.089171			
H		1.29	1.341901	1.274466			
H		1.52	1.265691	1.284256			
H		3.21	3.003812	3.100964			

Functional	Solvent?	Basis Set	Type of Data		
mPW1PW91	PCM	6-311+G(d,p)	Unscaled Shifts		

	Isomer 2'	Isomer 1'	Isomer 3	Isomer 4	Isomer 5	Isomer 6
sDP4+ (H data)	95.99%	4.01%	-	-	-	-
sDP4+ (C data)	100.00%	0.00%	-	-	-	-
sDP4+ (all data)	100.00%	0.00%	-	-	-	-
uDP4+ (H data)	99.56%	0.44%	-	-	-	-
uDP4+ (C data)	99.26%	0.74%	-	-	-	-
uDP4+ (all data)	100.00%	0.00%	-	-	-	-
DP4+ (H data)	99.98%	0.02%	-	-	-	-
DP4+ (C data)	100.00%	0.00%	-	-	-	-
DP4+ (all data)	100.00%	0.00%	-	-	-	-

2. Figures

Figure S1. ^{1}H NMR spectrum of compound **1** in MeOH-d_4.

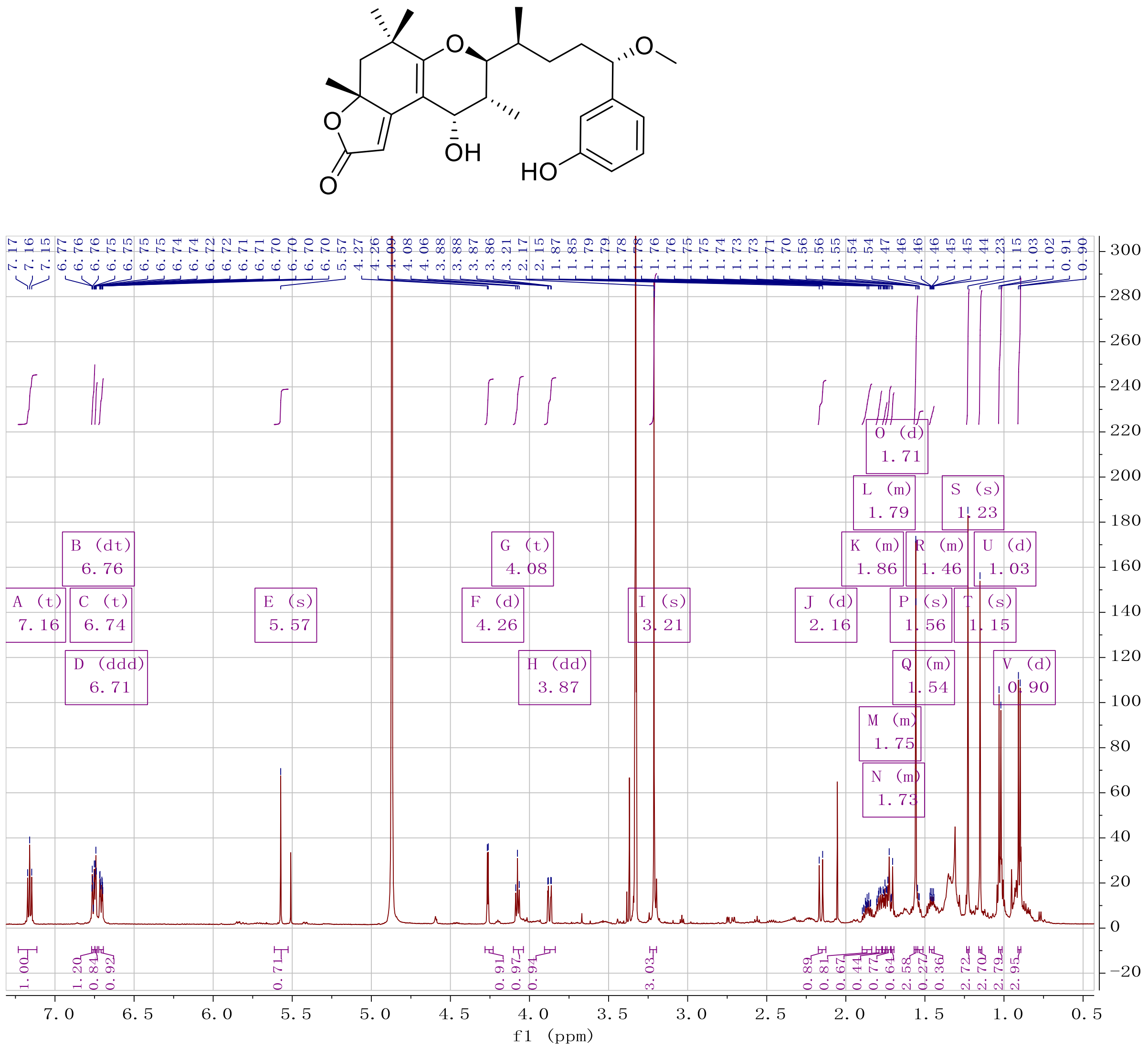

Figure S2. ^{13}C NMR spectrum of compound **1** in MeOH-d_4.

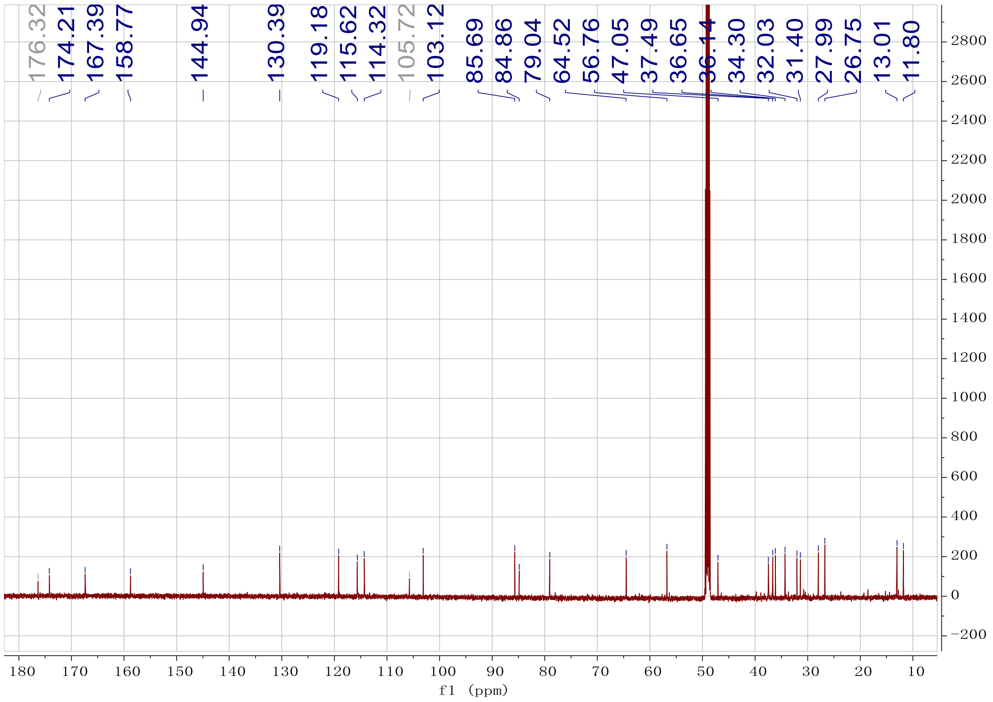

Figure S3. DEPT spectrum of compound **1** in MeOH- d_4.

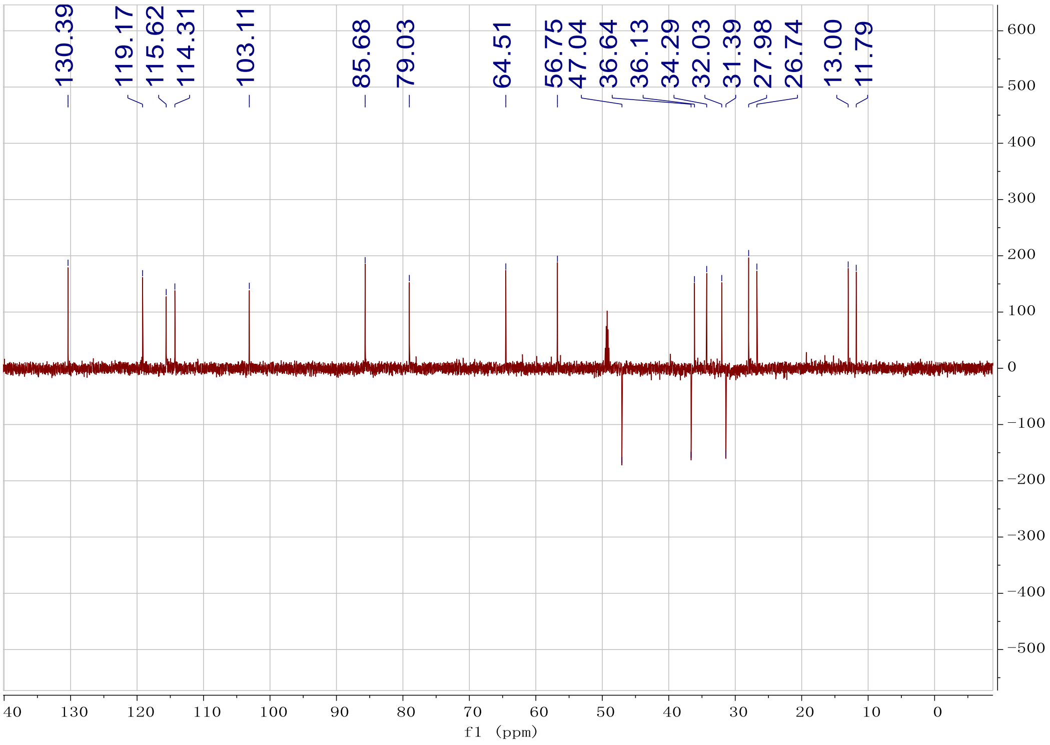

Figure S4. HSQC spectrum of compound **1** in MeOH- d_4.

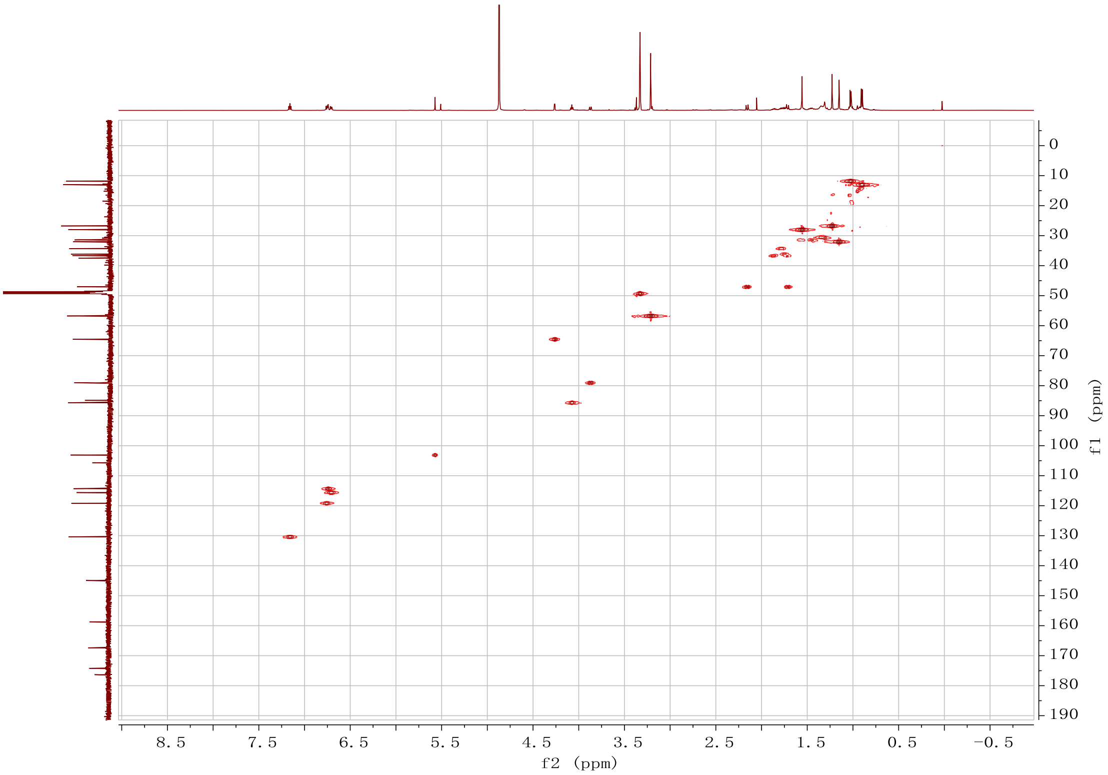

Figure S5. ^{1}H-^{1}H COSY spectrum of compound **1** in MeOH- d_4.

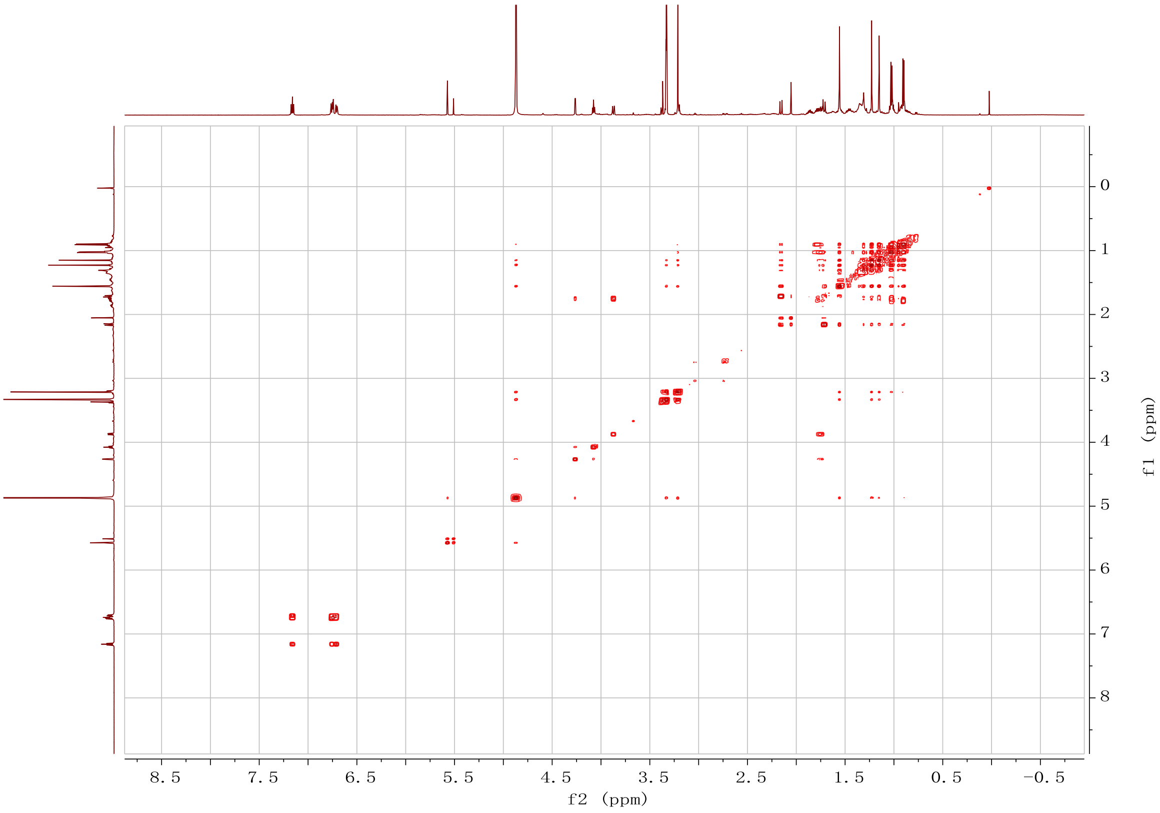

Figure S6. HMBC spectrum of compound **1** in MeOH- d_4.

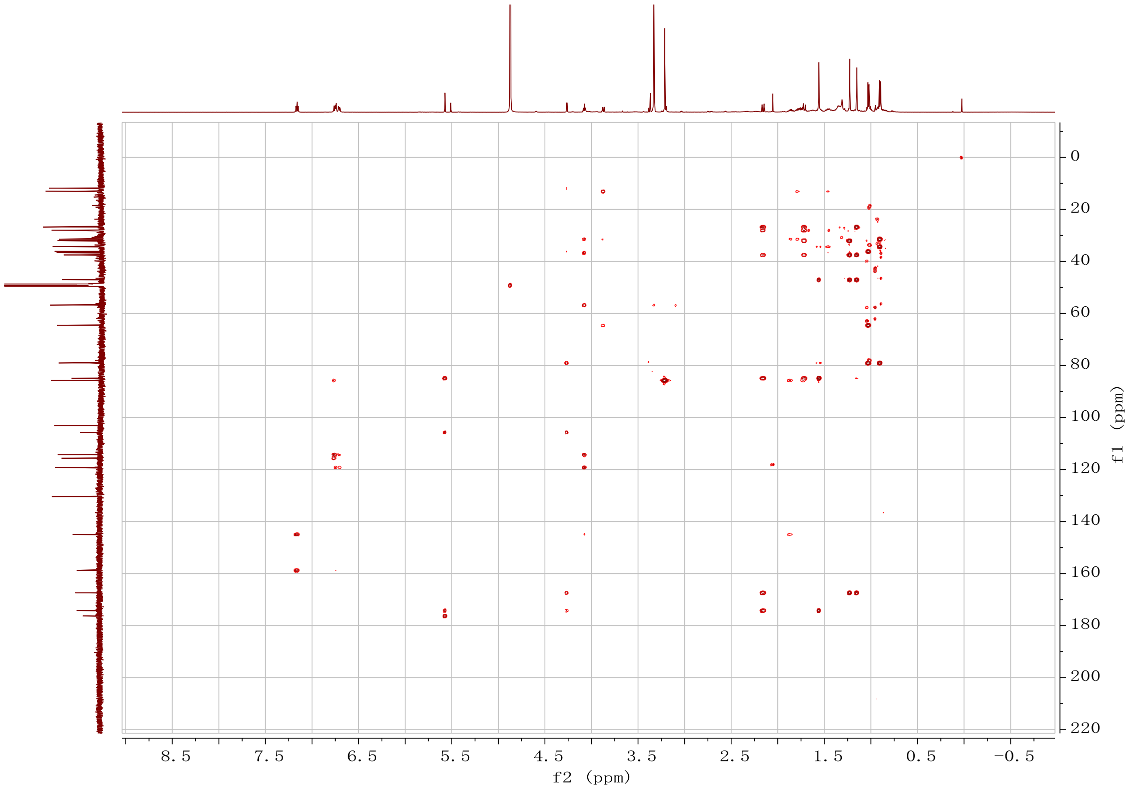

Figure S7. NOESY spectrum of compound **1** in MeOH- d_4.

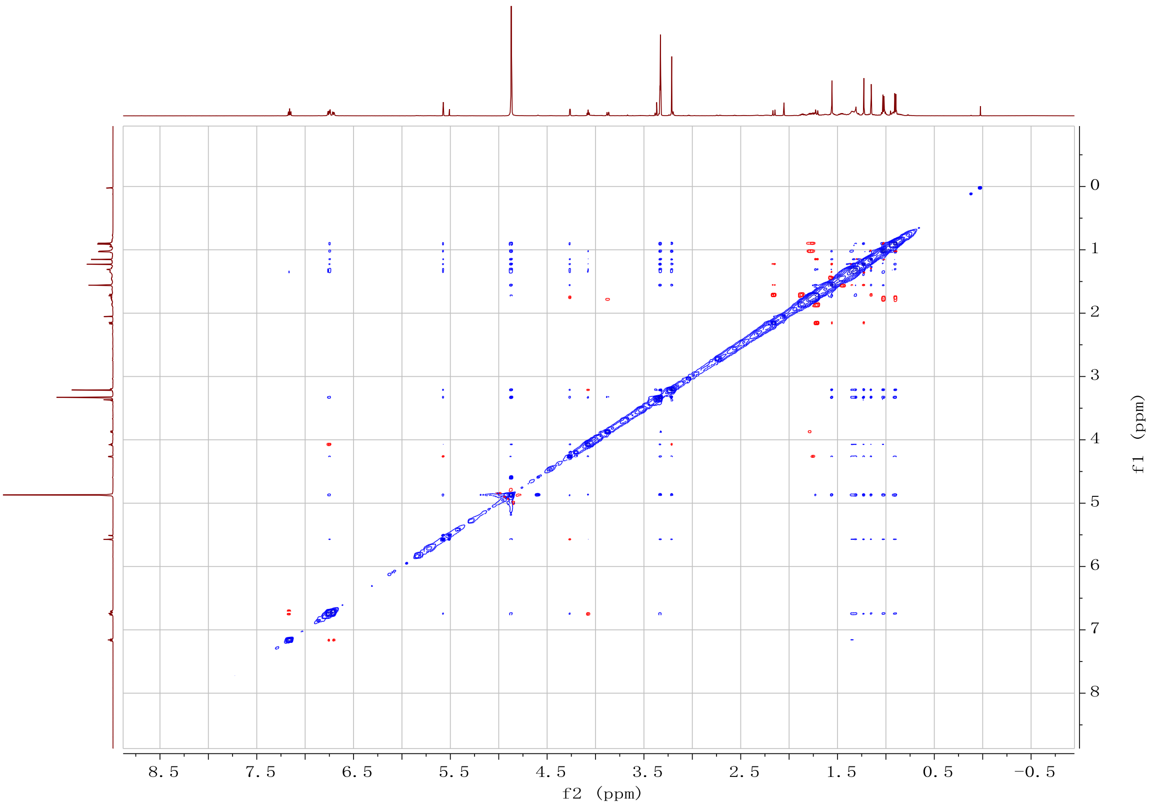

Figure S8. HRESIMS spectrum of compound **1** in MeOH- d_4.

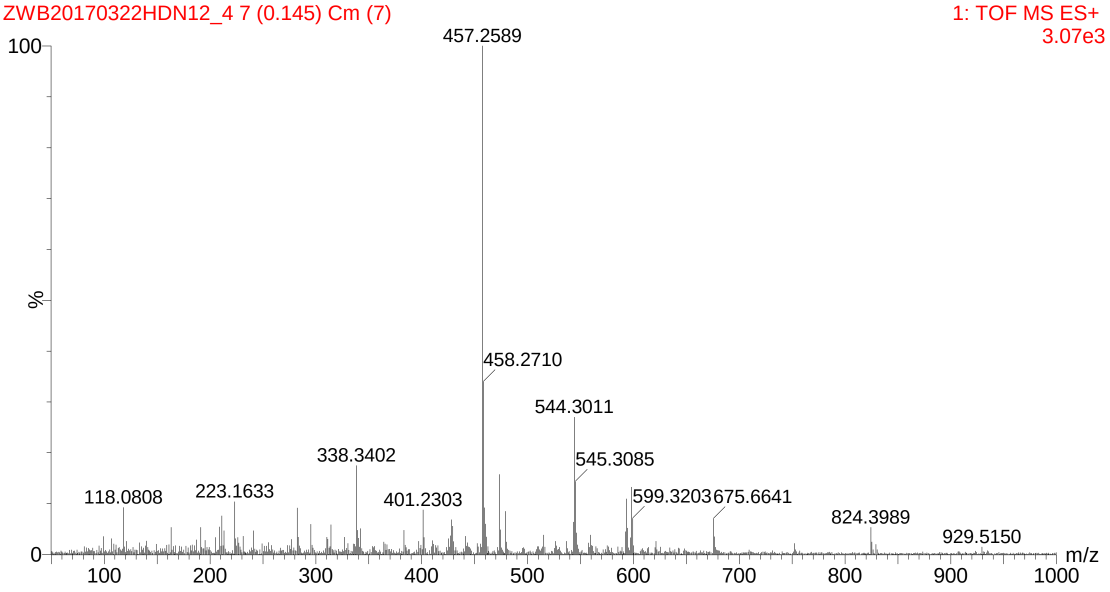

Figure S9. UV spectrum of Neo-debromoaplysiatoxin E (**1**) in MeOH.

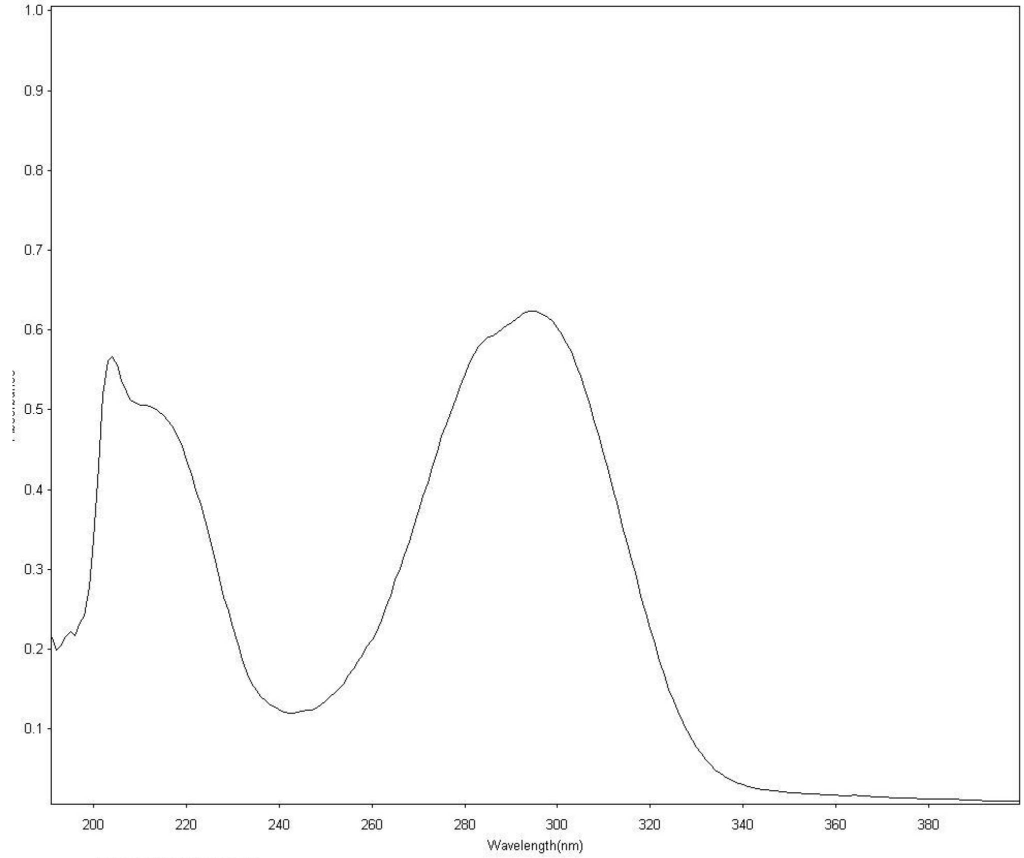

Figure S10. ^{1}H NMR spectrum of compound **2** in MeOH- d_4.

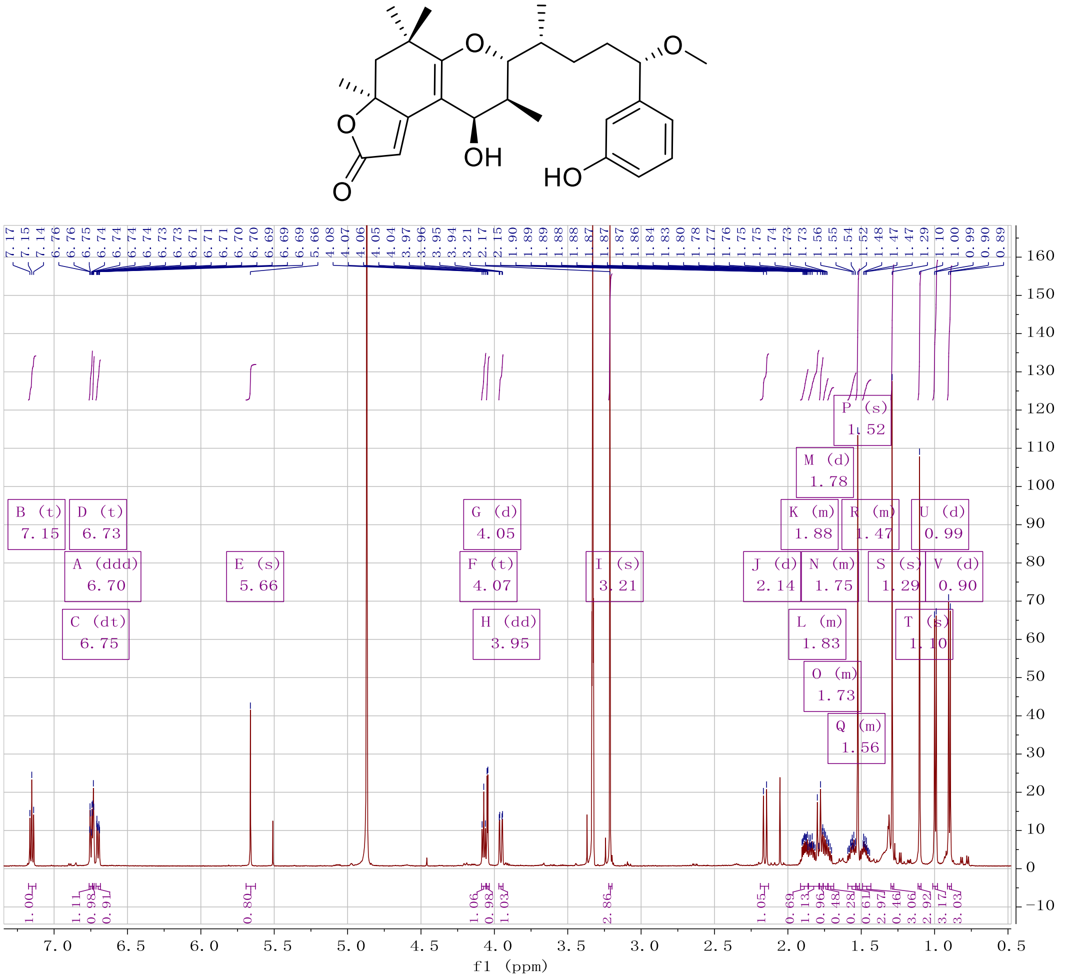

Figure S11. ^{13}C NMR spectrum of compound **2** in MeOH- d_4.

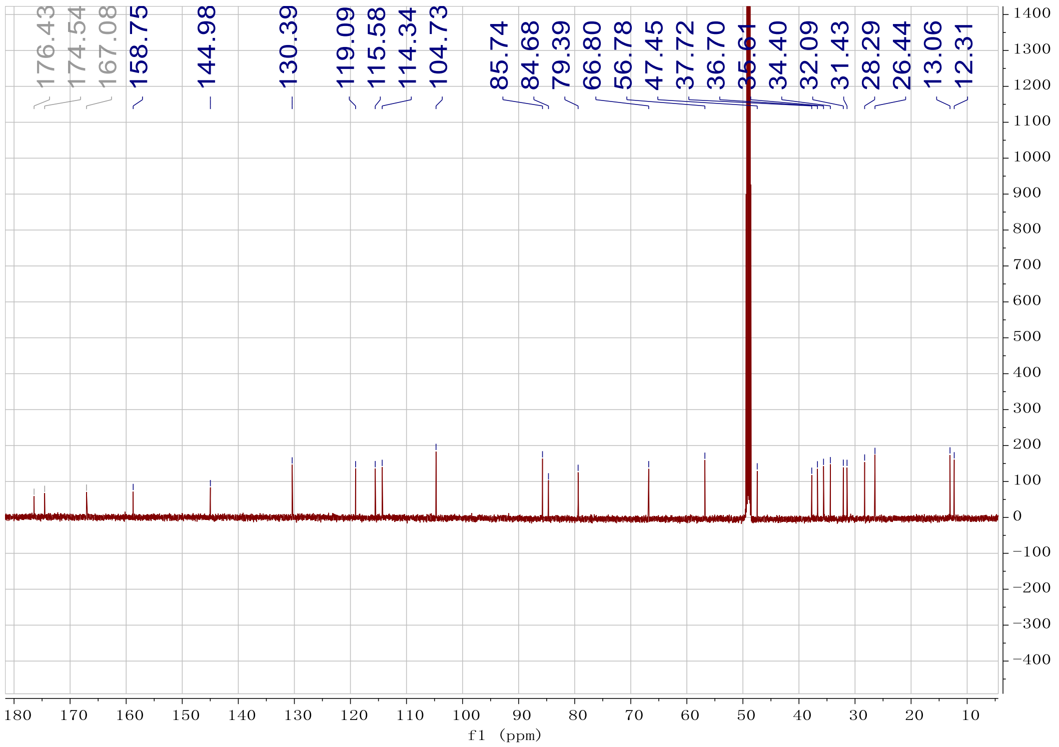

Figure S12. DEPT spectrum of compound **2** in MeOH- d_4.

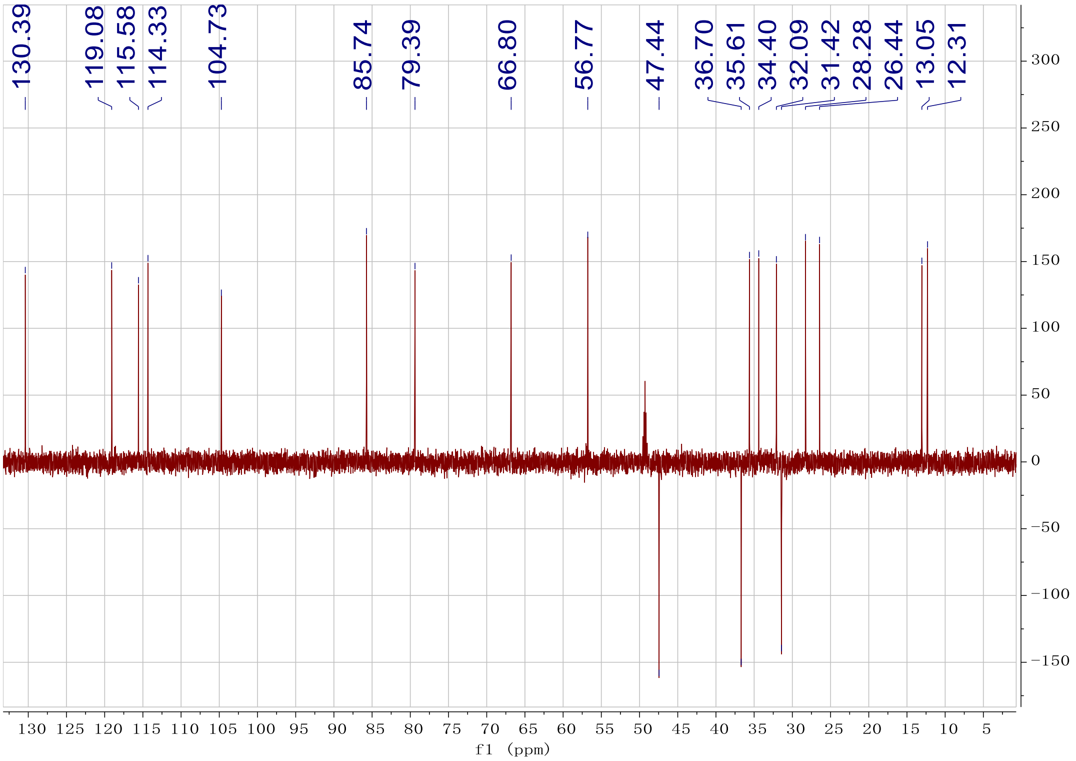

Figure S13. HSQC spectrum of compound **2** in MeOH- d_4.

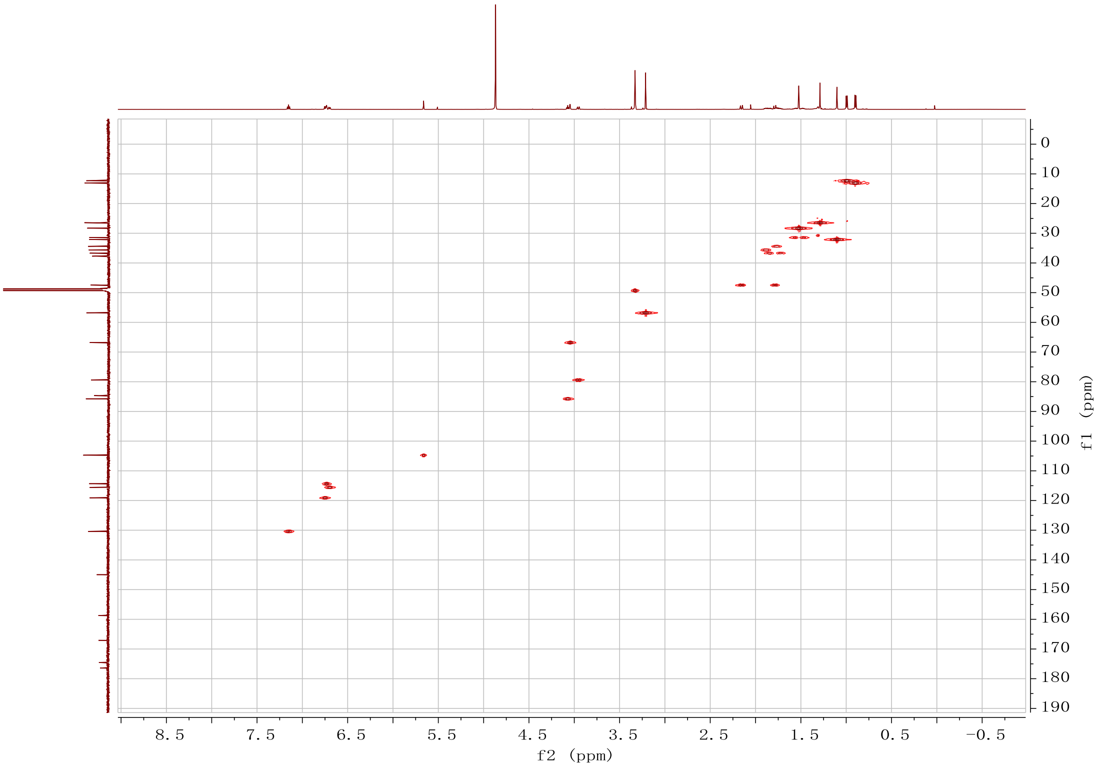

Figure S14. ^{1}H-^{1}H COSY spectrum of compound **2** in MeOH- d_4.

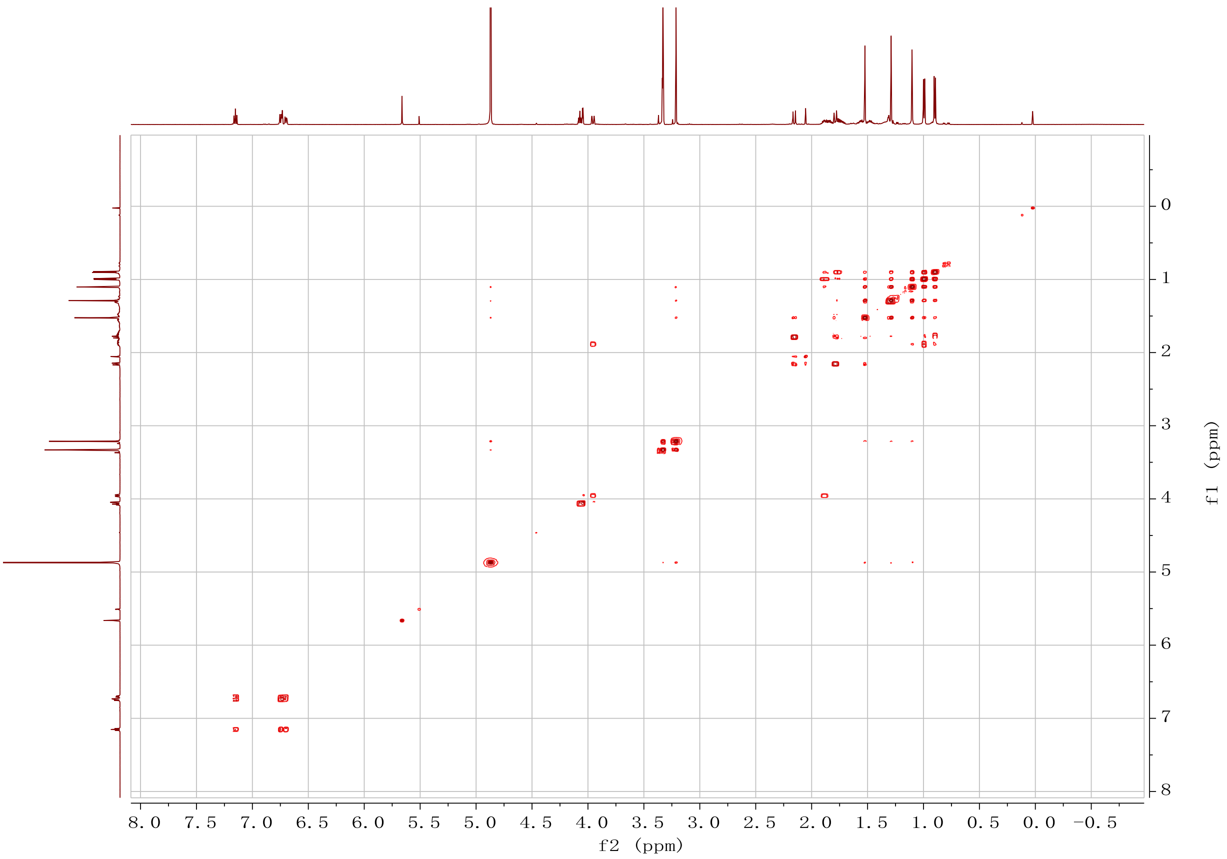

Figure S15. HMBC spectrum of compound **2** in MeOH- d_4.

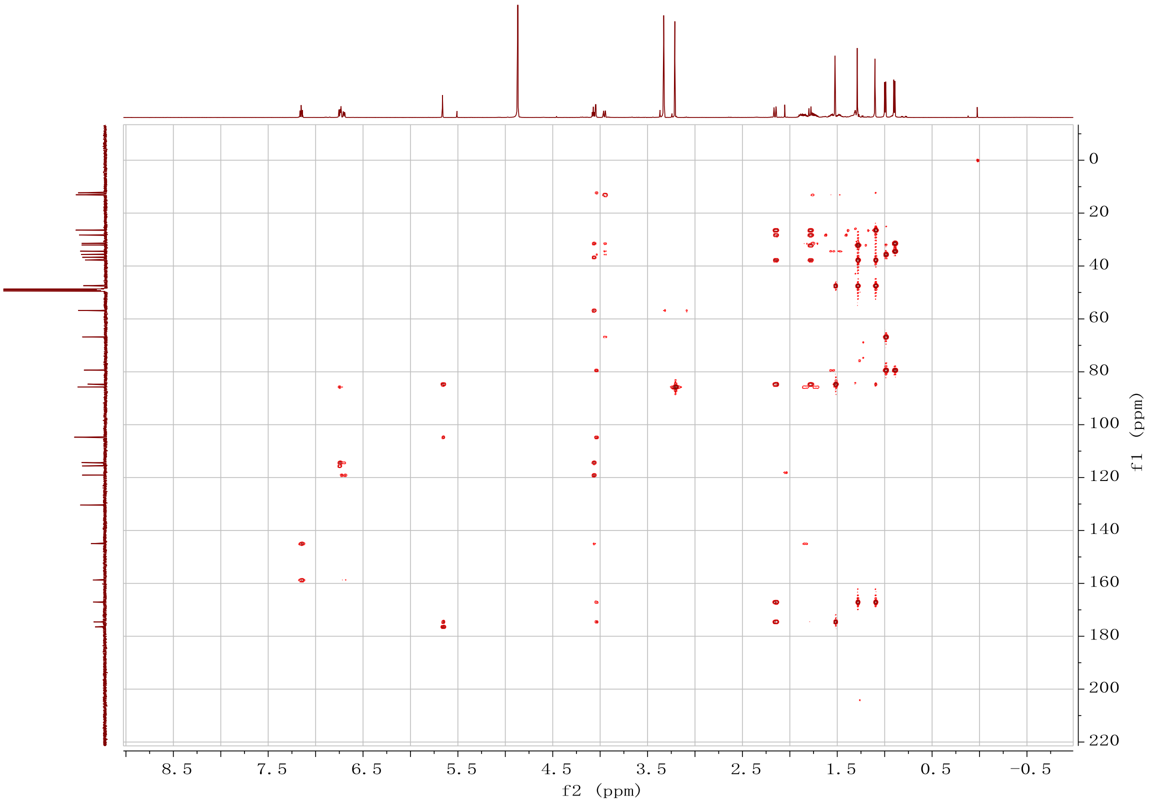

Figure S16. NOESY spectrum of compound **2** in MeOH- *d*4.

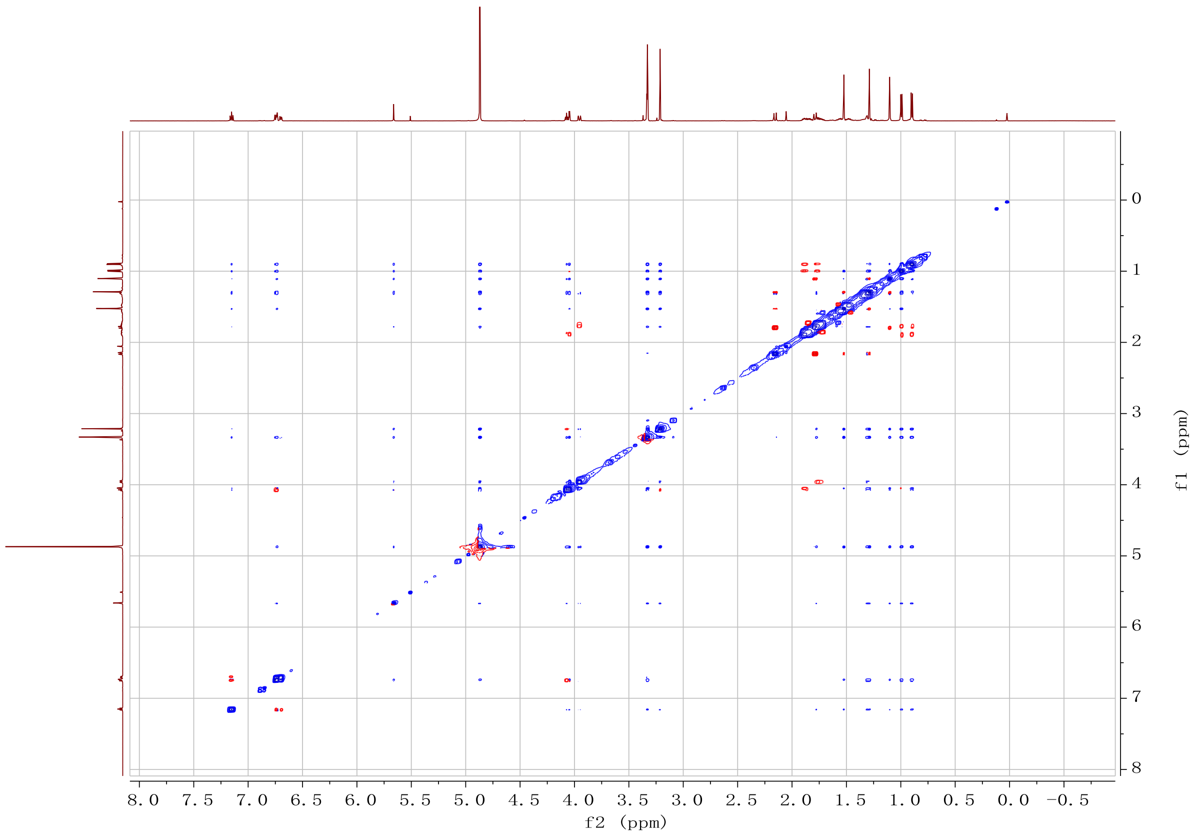

Figure S17. HRESIMS spectrum of compound **2** in MeOH- *d*4.

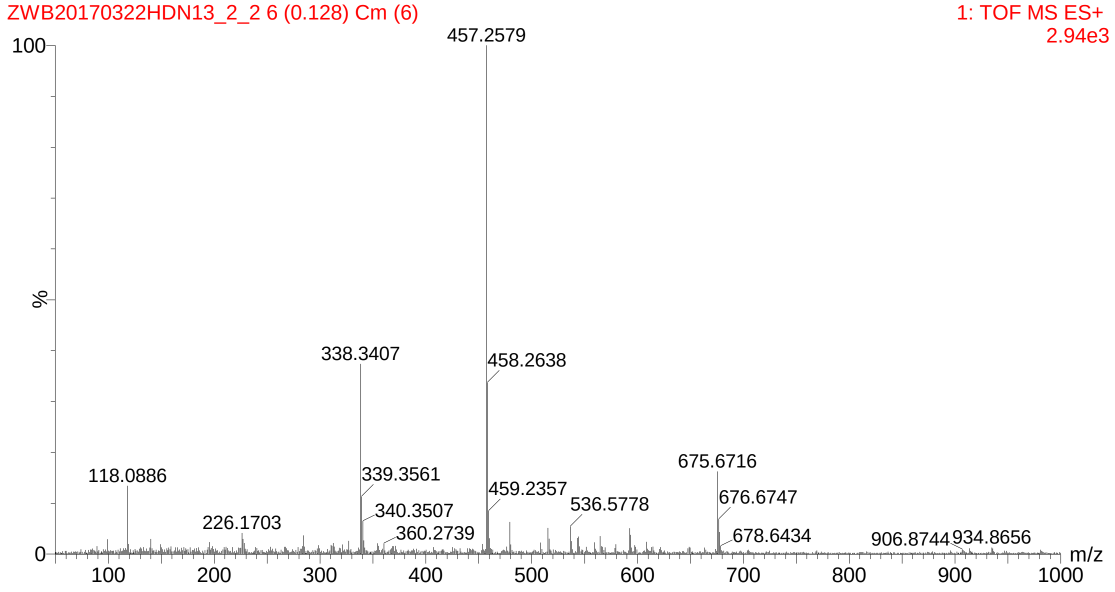

Figure S18. UV spectrum of Neo-debromoaplysiatoxin F (**2**) in MeOH.

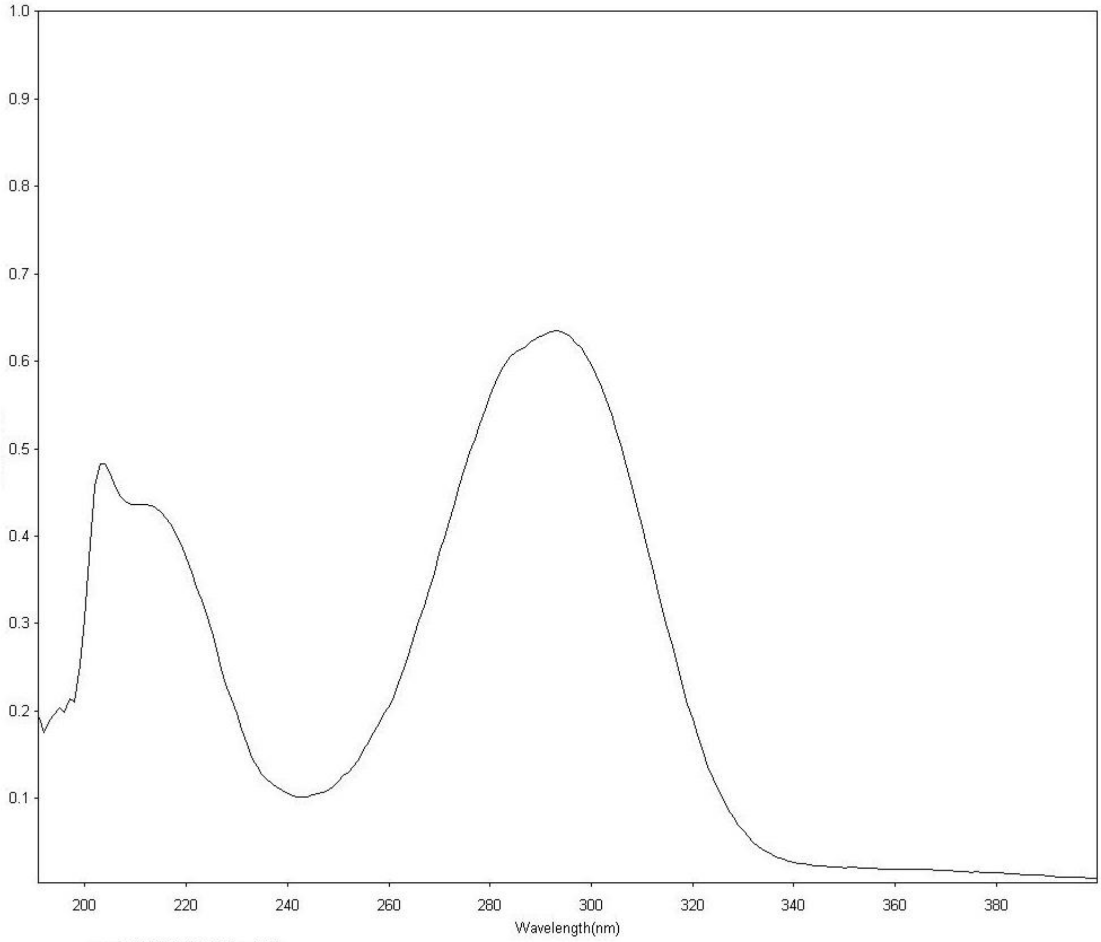

Figure S19. Plausible Biosynthetic Pathway of **1** and **2**.

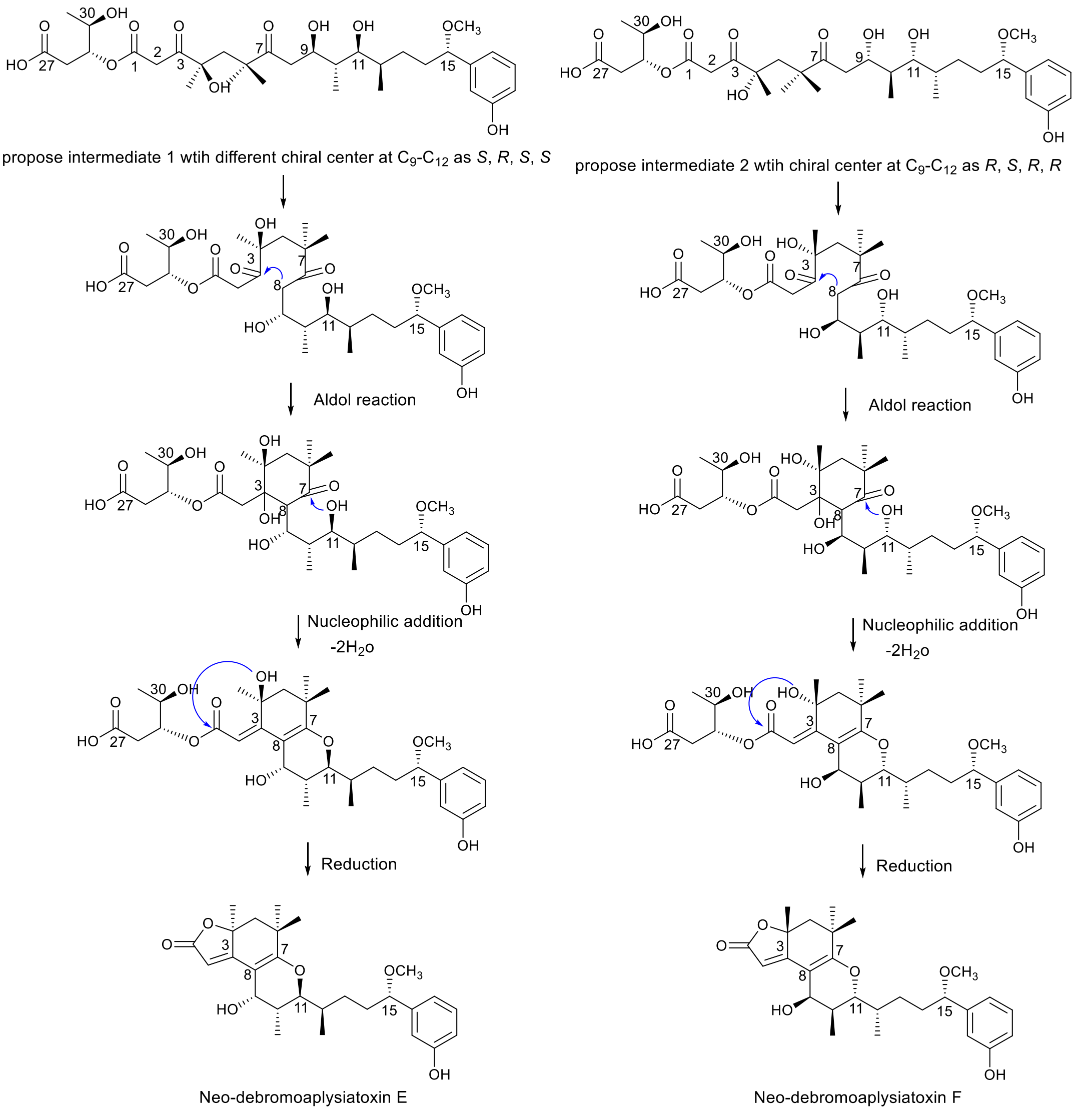

Supplement: Supplementary file 1 [file marinedrugs-17-00652-s001.pdf]
